# Dermoscopy for melanoma detection and triage in primary care: a systematic review

**DOI:** 10.1136/bmjopen-2018-027529

**Published:** 2019-08-20

**Authors:** OT Jones, LC Jurascheck, MA van Melle, S Hickman, NP Burrows, PN Hall, J Emery, FM Walter

**Affiliations:** 1 Department of Public Health and Primary Care, University of Cambridge, Cambridge, UK; 2 University of Cambridge School of Clinical Medicine, Cambridge, UK; 3 Norfolk and Norwich University Hospitals NHS Foundation Trust, Norwich, UK; 4 Addenbrooke's Hospital Department of Dermatology, Cambridge University Hospitals NHS Foundation Trust, Cambridge, UK; 5 Addenbrooke's Hospital, Cambridge University Hospitals NHS Foundation Trust, Cambridge, UK; 6 General Practice and Primary Care Academic Centre, University of Melbourne, Carlton, Victoria, Australia

**Keywords:** dermoscopy, melanoma, primary care, systematic review, skin cancer

## Abstract

**Objective:**

Most skin lesions first present in primary care, where distinguishing rare melanomas from benign lesions can be challenging. Dermoscopy improves diagnostic accuracy among specialists and is promoted for use by primary care physicians (PCPs). However, when used by untrained clinicians, accuracy may be no better than visual inspection. This study aimed to undertake a systematic review of literature reporting use of dermoscopy to triage suspicious skin lesions in primary care settings, and challenges for implementation.

**Design:**

A systematic literature review and narrative synthesis.

**Data sources:**

We searched MEDLINE, Cochrane Central, EMBASE, Cumulative Index to Nursing and Allied Health Literature, and SCOPUS bibliographic databases from 1 January 1990 to 31 December 2017, without language restrictions.

**Inclusion criteria:**

Studies including assessment of dermoscopy accuracy, acceptability to patients and PCPs, training requirements, and cost-effectiveness of dermoscopy modes in primary care, including trials, diagnostic accuracy and acceptability studies.

**Results:**

23 studies met the review criteria, representing 49 769 lesions and 3708 PCPs, all from high-income countries. There was a paucity of studies set truly in primary care and the outcomes measured were diverse. The heterogeneity therefore made meta-analysis unfeasible; the data were synthesised through narrative review. Dermoscopy, with appropriate training, was associated with improved diagnostic accuracy for melanoma and benign lesions, and reduced unnecessary excisions and referrals. Teledermoscopy-based referral systems improved triage accuracy. Only three studies examined cost-effectiveness; hence, there was insufficient evidence to draw conclusions. Costs, training and time requirements were considered important implementation barriers. Patient satisfaction was seldom assessed. Computer-aided dermoscopy and other technological advances have not yet been tested in primary care.

**Conclusions:**

Dermoscopy could help PCPs triage suspicious lesions for biopsy, urgent referral or reassurance. However, it will be important to establish further evidence on minimum training requirements to reach competence, as well as the cost-effectiveness and patient acceptability of implementing dermoscopy in primary care.

**Trial registration number:**

CRD42018091395.

**Strengths and limitations of this study**

- This study systematically reviews the published evidence for dermoscopy use by primary care physicians in primary care settings, including studies of acceptability and cost-effectiveness, as well as diagnostic accuracy studies.
- The use of a broad search strategy across multiple databases enabled us to identify 23 studies whose findings examine dermoscopy use in primary care clinical practice.
- The included studies were of varying quality.
- Due to the heterogeneity of the included papers, we were not able to undertake any meta-analysis; instead, we performed a narrative synthesis.

## Introduction

Worldwide malignant melanoma is the 15th most common cancer.^1^ Melanoma has one of the fastest rising incidence rates of any cancer, and among white populations incidence has quadrupled over the last 30 years. In the UK this is projected to rise by a further 7% between 2014 and 2035, reflecting increasing exposure to the main risk factor, ultraviolet radiation.^2^ There were nearly 300 000 new cases of melanoma worldwide in 2018.^1^

Primary care (the first point of contact for patients in the healthcare system, usually community-based) can play an important role in improving outcomes for patients with melanoma. More accurate triage of suspicious pigmented skin lesions could lead to more prompt diagnosis of melanoma at earlier stages and improved outcomes, and reduce unnecessary biopsies or referrals. Most people diagnosed with cancer first present in primary care,^3^ where primary care physicians (PCPs) need to distinguish rare melanomas from common benign lesions using clinical history taking and visual inspection, aided by checklists such as the 7-point checklist as recommended in the 2015 National Institute for Health and Care Excellence guidelines for suspected cancer.^4^ Various technologies may also have a role in assisting triage of suspicious skin lesions, including mobile phone applications,^5^ reflectance confocal microscopy,^6^ optical coherence tomography,^7^ computer-aided diagnosis,^8^ high-frequency ultrasound^9^ and dermoscopy.^10^

Dermoscopy (also referred to as dermatoscopy or epiluminescence microscopy) is a non-invasive technique using a hand-held magnifier and incident light, which may be polarised to reduce reflection, to reveal subsurface structures. Dermoscopy performed by trained specialists is more sensitive and specific in classifying skin lesions than clinical examination with the naked eye alone.^4 11^ Dermatologists and some international guidelines recommend PCPs use dermoscopy^12^; however, when used by untrained or less experienced clinicians, accuracy can be no better than inspection alone,^13^ and there is a danger of increased excisions, over-referral or false reassurance. It takes time to train clinicians to use dermoscopy, and PCP training dropout rates have been shown to be high.^14 15^ For these reasons dermoscopy is not currently recommended for use by PCPs in the UK,^4^ although it is used routinely by PCPs in Australia,^16^ which has the highest incidence of melanoma worldwide. Some digital dermoscopy devices exist, a few of which incorporate computer-aided diagnosis, but they are expensive, and while showing better sensitivity even in expert hands many have lower specificity than clinicians alone.^17^ However, recent research suggests computer-aided diagnostic tools have the potential to exceed the diagnostic performance of dermatologists.^18^

A Cochrane review of dermoscopy has recently been published and examines the diagnostic accuracy of dermoscopy, with and without visual inspection, for the detection of cutaneous invasive melanoma and intraepidermal melanocytic variants in adults.^19^ Our systematic review has a broader aim, focusing on the first presentation of suspicious skin lesions in primary care and whether dermoscopy and dermoscopy-related technologies, with suitable training, can be used accurately and effectively to triage suspicious skin lesions at this point in the healthcare pathway. We considered various types of dermoscopy technologies, including hand-held dermoscopy, computer-aided/digital dermoscopy devices and novel teledermoscopy approaches (ie, referral using electronic dermoscopy images or video). In addition to data on the diagnostic accuracy of dermoscopy, we looked for data on the practical challenges to implementing dermoscopy in primary care, including utility, acceptability to patients and PCPs, training requirements, and cost-effectiveness.

## Methods

This systematic review was conducted in accordance with the Preferred Reporting Items for Systematic Reviews and Meta-Analyses (PRISMA) guidelines,^20^ and the protocol was registered with PROSPERO prior to conducting the review.^21^ All aspects of the protocol were reviewed by senior faculty from the CanTest Collaborative (www.cantest.org).

We searched the MEDLINE, EMBASE, Cochrane Central, Cumulative Index to Nursing and Allied Health Literature, and SCOPUS databases using keywords related to dermoscopy, melanoma and primary care, without language restrictions, from 1 January 1990 to 31 December 2017. We also manually searched the reference lists of included studies. We included all types of study design as we anticipated that there would be few relevant randomised controlled trials (RCTs) or diagnostic accuracy studies performed in primary care, and we aimed to find additional qualitative evidence on barriers to the use of dermoscopy which may be found in non-RCT study designs. We chose to start the search from 1990 as this was when the earliest dermoscopy-related research emerged. We considered published evidence from any international healthcare system and whether it could be interpreted and applied to primary care settings, including the extent to which data collected from specialist clinic settings could be applied to the lower-prevalence primary care population.

We included all studies which provide evidence around test accuracy, utility, acceptability to patients and PCPs, training requirements, and cost-effectiveness of dermoscopy modes in primary care, including trials, diagnostic accuracy and acceptability studies. As our interest was in the use of dermoscopy by generalist clinicians, we included all studies reporting PCP use of dermoscopy; studies of secondary care physicians who were not trained in dermoscopy were assessed for the applicability of their study to answer the research question. We excluded studies that were based in any clinical setting other than at the first assessment of suspicious skin lesions, and any studies that were not considered primary studies.

Following duplicate removal, one author (OJ) screened titles and abstracts to identify studies which fitted the inclusion criteria. Of the titles and abstracts 10% were checked by two other authors (LJ and SH), and interassessor reliability was excellent, with disagreement for only 1 out of the 100 papers checked. Any disagreements were discussed by the core research team (OJ, LJ, SH, FMW) and a consensus reached. At least two reviewers (OJ, LJ, SH, MvM, FMW) independently assessed each full-text article for the possibility of inclusion in the review. Any disagreements were resolved by consensus-based discussion.

Data extraction was undertaken by two reviewers independently (OJ, LJ, FMW) and summarised using descriptive tables, discussion and consensus. We chose to extract only reported outcomes from the included papers, without calculating further quantitative measures of diagnostic accuracy from their data, unless already reported. Due to the heterogeneity of the included papers, we were not able to undertake any meta-analysis; instead, we chose to perform a narrative synthesis.

Risk-of-bias assessment was undertaken for each full-text paper by two independent researchers (OJ, LJ) using the Joanna Briggs Institute (JBI) critical appraisal tools.^22^ These tools incorporate various critical assessments for different study designs, including patient selection, randomisation, data collection and analysis. As assessments for different study designs had varying denominators, the score was converted to a percentage and classified as high, medium and low risk to aid clarity of presentation and interpretation. Although the studies demonstrated a wide range in quality, no studies were excluded based on their risk-of-bias assessment. Full details of our review question, search strategy, inclusion/exclusion criteria, methodology for data extraction, risk-of-bias assessment and outcomes extraction are described in online supplementary appendices 1 and 2, as well as a full list of excluded studies (online supplementary appendix 3).

10.1136/bmjopen-2018-027529.supp1 Supplementary data

### Patient and public involvement

Our long-standing collaborator, Mrs Margaret Johnson, is a patient advocate. She commented regularly on the study from its conception, including aspects of the research question, outcome measures and study design. There was no patient recruitment required for this study. The results will be disseminated to patient advocates, groups and relevant charities.

## Results

**Figure 1:**
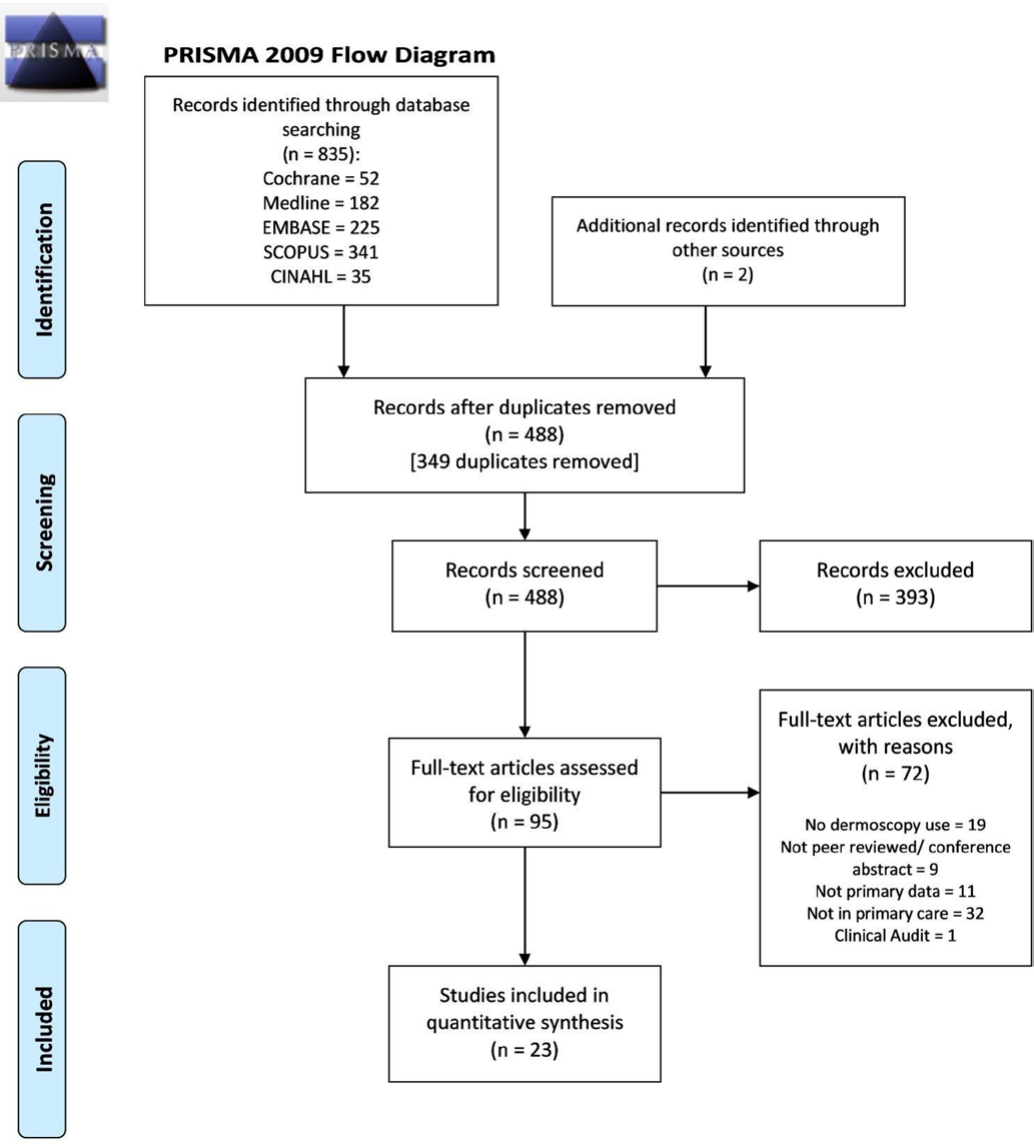
PRISMA flow diagram for the studies included in the review. CINAHL, Cumulative Index to Nursing and Allied Health Literature; PRISMA, Preferred Reporting Items for Systematic Reviews and Meta-Analyses.

Figure 1 shows the study PRISMA diagram. There were 837 studies identified, of which 349 were duplicates. Ninety-five articles underwent full-text review and 23 met the inclusion criteria.^14 23–44^ These 23 articles reported data relating to 49 769 lesions and 3708 PCPs.

Table 1 provides a summary of the study characteristics for included studies. We included three RCTs, two sequential intervention trials (SIT), nine diagnostic accuracy studies, two cohort studies, two case series, one case–control study and four PCP surveys. Table 1 also visually summarises the practitioner and patient populations reported in the studies and highlights the paucity of studies reporting PCPs using dermoscopy with primary care patients (5 out of 16). Studies of teledermoscopy-based referral systems were more frequently set in primary care, with six out of seven studies involving primary care clinicians and primary care patients. Overall, 16 of the 23 papers reported studies of PCPs, but only 11 papers reported studies involving primary care patients.

**Table 1:** Study demographics: patient and practitioner populations

Study details	Location	Study type	Practitioners				Patients						Control group	Intervention group	Gender of patients (except where indicated) (F/M) (%)	Age of patients (except where indicated) (years), mean (range)
			PCPs	SCPs	PCPs and SCPs	DPCSCCs	Studies with patients from:			Studies using images from:						
							Primary care	Secondary care	DPCSCCs	Primary care	Secondary care	DPCSCCs				
Dermoscopy papers																
Ahmadi *et al* 27	Maastricht/Limburg, The Netherlands	Case series											None	Patients from 3 primary care practices	F=57.8	54.7 (60–79)
Argenziano *et al* 23	Barcelona, Spain; Naples, Italy	RCT											Naked eye	Dermoscopy	C: F=62.4 I: F=62.3	C: 40 (2–90) I: 41 (3–94)
Bourne *et al* 28	Brisbane, Australia	DA study											Clinical assessment and algorithms	BLINCK algorithm	F=52.2	58 (30–60)
Chappuis *et al* 38	4 regions of France	Survey											None	PCPs in France	GPs: F=42.4	GPs: <30=8 30–50=169 >50=246
Koelink *et al* 24	Groningen, The Netherlands	RCT											Naked eye	Dermoscopy	C: F=61.6 I: F=68.2	C: 54.7 I: 53.2
Menzies *et al* 29	USA, Germany and Australia	DA study											Independent clinicians	SolarScan assessment	NR	NR
Menzies *et al* 14	Perth, Australia	SIT											PCP decision before intervention	Outcome after dermoscopy and SDDI	NR	NR
Morris *et al* 39	Florida, USA	Survey											None	PCPs	Clinicians: F=41.6	Clinicians: median 40–49 years
Morris *et al* 40	Florida, USA	Survey											None	Practising physicians	Clinicians: F=34.7	Clinicians: NR
Pagnanelli *et al* 30	Rome, Italy	DA study		*									Pretraining	Post-training	NR	NR
Rogers *et al* 31	New York, USA	DA study											Histology/expert opinion	Clinicians using 3 algorithms	F=53.3	Median 31–40 years
Rogers *et al* 32	New York, USA	DA study											Histology/expert opinion	Clinicians using 3 algorithms	F=53.3	Median 31–40 years
Rosendahl *et al* 43	Queensland, Australia	SIT	†										Naked eye	Dermoscopy images	F=32.6	57 SD: 17 years
Rosendahl *et al* 25	Australian SCARD database	Cohort study											Histology diagnosis	PCP decision	NR	NR
Secker *et al* 44	Leiden, The Netherlands	DA study											PCPs before education	After education	F=51.8	45.2 (28–63)
Westerhoff *et al* 33	Sydney, Australia	DA study											PCP diagnosis	PCPs ± dermoscopy ± education	NR	NR
Teledermoscopy papers																
Börve *et al* 34	Gothenburg, Sweden	Case–control study											Paper-based referrals	Teledermoscopy referrals	C: F=57.1 I: F=61.4	C: 61 (18–97) I: 54 (18–93)
Ferrándiz *et al* 26	Andalucia, Spain	RCT											Clinical images	Clinical and dermoscopy images	C: F=52.88 I: F=62.28	C: 57.33 I: 54.96
Grimaldi *et al* 35	Siena, Italy	DA study											Judgement before dermoscopy	Judgement after dermoscopy	NR	NR
Livingstone and Solomon^41^	Ruislip, UK	Case series											Expert diagnosis and standard costs	Teledermoscopy referrals	NR	NR
Moreno-Ramirez *et al* 36	Sevilla, Spain	DA study											Tele dermatology referrals	Same patients + dermoscopy images	F=70.5	38.8 (1–73)
Stratton and Loescher^42^	Arizona, USA	Survey											None	Nurse practitioners	Nurse practitioners: F=92	Nurse practitioners: 48
van der Heijden *et al* 37	Amsterdam, The Netherlands	Cohort study											Face-to-face consult ± histology	Teledermoscopy consult (same patients)	F=55	Median 47 years, 6–84 years

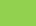
Coloured boxes denote that practitioners and patients from these populations were included in the corresponding study

*Minimal dermoscopy experience, although secondary care physicians.

†Practitioner population not specified.

BLINCK, Benign, Lonely, Irregular, Nervous, Change, Known clues; C, control group; DA, diagnostic accuracy; DPCSCC, dedicated primary care skin cancer clinic; F, female; GP, General Practitioner; I, intervention group; M, male; NR, not reported; PCP, primary care physician; RCT, randomised controlled trial; SCARD, Skin Cancer Audit Research Database; SCP, secondary care physician; SD, Standard Deviation; SDDI, short-term sequential digital dermoscopy imaging; SIT, sequential intervention trial.

Table 2 summarises the outcome measures of each included study, grouped into accuracy and reliability outcomes and implementation outcomes, and shows the heterogeneous nature of the reported outcomes. The accuracy and reliability outcomes were diverse; 12 papers reported sensitivity and specificity, 8 reported diagnostic accuracy or area under the curve, 5 reported positive and negative predictive values, 14 reported the proportion of correct decisions, 4 reported the number needed to excise, and 5 reported the biopsy rate. The implementation outcomes were less numerous but also quite diverse: 4 papers reported on PCP opinions, 3 performed cost-effective analyses, 2 looked at response times for teledermoscopy services, 2 looked at image quality for teledermoscopy, and 1 assessed patient satisfaction.

**Table 2:** Diversity of reported outcomes and critical appraisal results of the included studies

Study	Accuracy and reliability outcomes									Implementation outcomes					JBI critical appraisal checklists^22^					
	Sensitivity and specificity	DA/ AUC	PPV and NPV	Correctly diagnosed lesions	NNE	Biopsy rate	Inter observer agreement	Inter instrument reliability	OR/ relative risk	Survey/ PCP opinion	Cost-effective analysis	Response time for TDS	Patient satisfaction	Image quality for TDS	Diagnostic test accuracy studies	RCTs	Analytical cross- sectional studies	Quasi- experimental studies	Case series	Cohort studies
RCTs and sequential intervention trials																				
Argenziano *et al* 23																				
Koelink *et al* 24																				
Menzies *et al* 14																				
Rosendahl *et al* 43																				
Ferrándiz *et al* 26																				
Non-RCT diagnostic accuracy studies																				
Ahmadi *et al* 27																				
Bourne *et al* 28																				
Menzies *et al* 29																				
Pagnanelli *et al* 30																				
Rogers *et al* 31																				
Rogers *et al* 32																				
Rosendahl *et al* 25																				
Secker *et al* 44																				
Westerhoff *et al* 33																				
Börve *et al* 34																				
Grimaldi *et al* 35																				
Livingstone and Solomon^41^																				
Moreno-Ramirez *et al* 36																				
van der Heijden *et al* 37																				
Survey-based studies																				
Chappuis *et al* 38																				
Morris *et al* 39																				
Morris *et al* 40																				
Stratton and Loescher^42^																				

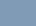
denotes that these accuracy and reliability outcomes were reported in this study; 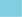
denotes that these implementation outcomes were reported in this study

Key to JBI score: 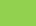; >60%: 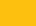; <30%: 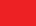.

AUC, area under the curve; DA, diagnostic accuracy; JBI, Joanna Briggs Institute; NNE, number needed to excise; NPV, negative predictive value;OR, Odds Ratio; PCP, primary care physician; PPV, positive predictive value; RCTs, randomised controlled trials; TDS, teledermoscopy.

Risk-of-bias outcomes from the JBI critical appraisal tools are included in table 2, demonstrating a wide range in quality across the studies. No studies were excluded based on the risk-of-bias assessment.

Tables 3 summarises the diagnostic accuracy results, with the studies grouped into RCTs and SITs, non-RCT diagnostic accuracy studies, and survey studies. Among the RCTs and SITs, Argenziano *et al*,^23^ Koelink *et al*,^24^ Rosendahl *et al* 43 and Menzies *et al* 14 found that dermoscopy reduced the number needed to excise to diagnose a melanoma. Ferrándiz *et al* 26 evaluated the impact of adding dermoscopic images to the standard teledermatology referral system and found that it improved accuracy and confidence in diagnosing skin lesions.

**Table 3A:** Summarised results of the RCTs and SITs

Study	Summary	Control (C)/Intervention (I)(number of lesions)	Outcome measures					
			Healthcare professional diagnosis	Expert/Histopathology diagnosis		Sens/Spec	PPV/NPV	Others
RCTs and SITs								
Argenziano *et al* 23	RCT in primary care comparing PCPs using naked-eye observation (ABCD) with PCPs using dermoscopy (3-point checklist).	C (1325)	Non-susp=925 Susp=408	39 susp 46 susp	23 malig 30 malig	Sens 54.1% Spec 71.3%	PPV 11.3% NPV 95.8%	2/6 MMs missed.
		I (1203)	Non-susp=824 Susp=379	16 susp 61 susp	6 malig 33 malig	Sens 79.2% Spec 71.8%	PPV 16.1% NPV 98.1%	1/6 MMs missed.
Koelink *et al* 24	Cluster RCT in primary care comparing PCP diagnosis with naked-eye examination and dermoscopy examination.	C (230)	Non-susp=67 Referred=20 Biopsy/excision=135	Correctly diagnosed: MMs 22.2% (2/9) Lesions 40.5% (90/222)				All lesions OR=1.51 Relative risk=1.25 MM OR=5.52
		I (207)	Non-susp=84 Referred=18 Biopsy/excision=92	Correctly diagnosed: MMs 61.5% (8/13) Lesions 50.5% (98/194) 3 skin cancers incorrectly treated				
Menzies *et al* 14	SIT using within-lesion controls in primary care assessing effect of dermoscopy and SDDI on management of suspicious PSLs by PCPs.	C (374)	374 PSLs suspicious for referral ± excision	42 malignant lesions 33 MM, 1 MM in situ		For MM: Naked-eye: sens 37.5%, spec 84.6%, PPV 20.7, NPV 92.7		56.4% reduction in suspicious PSLs excised/referred. 63.5% reduction in benign excised PSLs. 1 MM in situ incorrectly managed in intervention group. Benign:MM ratio of excised/referred lesions 9.5:1 vs 3.7:1 (after dermoscopy) vs 3.5:1 (dermoscopy + SDDI) (p<0.0005).
		IA (374)	After dermoscopy 110 referred, 192 SDDI, 72 observed for change			Dermoscopy: sens 53.1%, spec 89.0%, PPV 34, NPV 94.7		
		IB (192)	Of 192 SDDI, 46 referred/excised, 6 continued SDDI (2 subsequently referred, 4 observed), 140 observed (5 subsequently referred, 135 observed)			+SDDI: sens 71.9%, spec 86.6%, PPV 36.4, NPV 96.6		
						Overall: Increased sens for all malignancies (40%–67.5%, p=0.014) and MM (37.5%–71.9%, p=0.006) following intervention. Increased PPV for MM (20.7%–36.4%, p=0.055) and NPV for MM (92.7%–96.6%, p=0.041).		
Rosendahl *et al* 43	SIT using within-lesion controls. Comparison of PSL diagnosis of ‘blinded observers’ using macroscopic images, then dermoscopy images.	C (463)	Single best diagnosis matched HP diagnosis in 320 cases (69.1%)	29 MMs, 72 BCCs, 5 SCCs NB: all PSLs excised		To achieve 80% spec, 70.5% sens		AUC 0.83 (malignant neoplasms). AUC 0.71 (melanocytic lesions).
		I (463)	Single best diagnosis matched HP diagnosis in 375 cases (80.1%)(p<0.001)			To achieve 80% spec, 82.6% sens (NS)		AUC 0.89 (malignant neoplasms) (p<0.001). AUC 0.76 (melanocytic lesions) (NS).
Ferrándiz *et al* 26	RCT comparing DA and cost-effectiveness of clinical teleconsultations with clinical + dermoscopic teleconsultations from 5 primary care centres.	C (226)	70.36% non-susp 45.14% referred for face-to-face evaluation	2.77% MM, 11.54% non-MM skin cancer		Sens 86.57% Spec 72.33%	PPV 56.98 NPV 92.86 Accuracy index 79.20%	False negative rate 13.43%. False positive rate 22.16%. 69.71% decisions made with higher confidence.
		I (228)	73.24% non-susp, 20.18% referred for face-to-face evaluation (p<0.001)	2.19% MM, 7.89% non-MM skin cancer		Sens 92.86% Spec 96.24%	PPV 84.38 NPV 98.17 Accuracy index 94.30% (p<0.001)	False negative rate 7.14%. False positive rate 3.76%. 78.07% decisions made with higher confidence (p=0.001).

**Table 3B:** Summarised results of the included non-RCT DA studies

Study	Summary	Intervention or group (number of lesions)	Outcome measures				
			Healthcare practitioner diagnosis	Expert review	Sens/Spec	PPV/NPV	Others
Non-RCT DA studies							
Ahmadi *et al* 27	Retrospective cross-sectional study of medical files from 3 general practices.	(580)	67 malignant, 75 premalignant, 399 non-suspicious, 39 unknown. 16.7% of patients referred.	151 lesions confirmed by HP/dermatology: 37 BCC, 4 MMs, 1 lentigo maligna, 20 unknown.	PPV: benign lesions 85.7%, premalignant lesions 18.2%, malignant lesions 53.8%, BCCs 53.3%, melanoma 25%.		Tools used: dermoscopy 8.4%, experienced PCP advice 1.4%, biopsy 1.9%, excision 10.3%
Bourne *et al* 28	Sequential design DA study. 4 PCPs used new BLINCK dermoscopy algorithm on 50 lesion images, compared with same PCPs using 3-point checklist, Menzies and clinical assessment on same 50 images.	BLINCK (200)	Found 33/36 MMs. Biopsied 131/200 lesions.	Images of 50 lesions used: 1 invasive MM (0.52 mm), 8 in situ.	Sens 90.8% Spec 50%		DA 65.5% Number-needed-to-excise 6
		3PCL (200)	Found 19/36 MMs. Biopsied 105/200 lesions.		Sens 59.4% Spec 42.2%		DA 48.3% Number-needed-to-excise 11
		Menzies (200)	Found 16/36 MMs. Biopsied 71/200 lesions.		Sens 54.7% Spec 69%		DA 63.9% Number-needed-to-excise 13
		Clinical (200)	Found 9/36 MMs. Biopsied 74/200 lesions.		Sens 52.6% Spec 74.8%		DA 65% Number-needed-to-excise 22
Menzies *et al* 29	Sequential design DA study comparing the performance of SolarScan with that of clinicians with varying dermatology experience on 78 images of PSLs.	Dermatologist (78)	10.5		Diagnosing MM: Sens 81%, spec 60%, PPV 30, NPV 94 Decision to excise: Sens 79%, spec 60%, PPV 29, NPV 93		
		PCP (78)	8		Diagnosing MM: Sens 62%, spec 63%, PPV 26, NPV 89 Decision to excise: Sens 62%, spec 61%, PPV 25, NPV 89		
		SolarScan (78)	11.1		Diagnosing MM: sens 85%, spec 65%, PPV 32, NPV 96 Decision to excise: sens 85%, spec 65, PPV 32, NPV 96		SolarScan’s sensitivity was higher than PCPs but NS.
Pagnanelli *et al* 30	Sequential design DA study to assess if internet-based course suitable to teach dermoscopy to 16 clinicians with minimal dermoscopy experience looking at 20 images of PSLs.	Control: pretraining (20)		20 PSLs in test set: 6 MMs, 14 non-MMs.	Pattern analysis: Sens 67.7%, spec 76.3%		DA 49.8 k-intraobserver agreement 0.42
					ABCD: Sens 58%, spec 73.4%		DA 38.8 k-intraobserver agreement 0.31
					7-point checklist: Sens 100%, spec 67.5%		DA 60.2 k-intraobserver agreement 0.58
					Menzies method: Sens 80.4%, spec 72%		DA 53.5 k-intraobserver agreement 0.50
		Intervention: post-training (20)			Pattern analysis: Sens 82%, spec 78.5%		DA 60.1 k-intraobserver agreement 0.58
					ABCD: Sens 78.4%, spec 79.6%		DA 56.6 k-intraobserver agreement 0.55
					7-point checklist: Sens 100%, spec 69.8%		DA 64.5 k-intraobserver agreement 0.61
					Menzies method: Sens 93.4%, spec 76%		DA 62.8 k-intraobserver agreement 0.66
Rogers *et al* 31	Sequential design DA study examining performance of new TADA when used by 120 clinicians of various specialties looking at 50 PSL images.	(50)	5641 lesion evaluations performed: 3034 malignant, 2607 non-suspicious.	50 lesion images in test set, 23 benign, 27 malignant. Sens and spec calculated for malignant lesions.	Dermatologists: Sens 94.8%, spec 78.5%		
					Non-dermatologists: Sens 93.7%, spec 72.1%		
					>1 year dermoscopy experience: Sens 95.4%, spec 77.3%		
					<1 year dermoscopy experience: Sens 91.3%, spec 74.2%		
Rogers *et al* 32	Sequential design DA study comparing performance of new TADA with existing dermoscopy algorithms when used by 120 clinicians of various specialties.	(50)	No data for 3-point checklist and AC rule. TADA: 5646 lesions evaluated. 2056 deemed non-suspicious (1891 true negatives (92.0%), 165 false negatives (8.0%)). 3590 deemed malignant (2871 true positives (80.0%), 719 false positives (20.0%)).	50 lesion images in test set, 23 benign, 27 malignant. Sens and spec based on 40 non-PSLs.	TADA: Sens 94%, spec 75.5%		Sens for MM with TADA 94%. Spec for untrained clinicians for benign PSLs using TADA 76%–94% (beginners can be quickly trained to identify benign lesions).
					AC: Sens 88.6%, spec 78.7%		
					3-point checklist: Sens 71.9%, spec 81.4%		
					Untrained (using TADA): Sens 93.6%, spec 69%		
					Trained (using TADA): Sens 95.4%, spec 73.2%		
Rosendahl *et al* 25	Prospective cohort study using SCARD to assess impact of dermoscopy use and subspecialisation on MM diagnosis by PCPs.	Dedicated skin cancer practitioners	Number of lesions seen not recorded on SCARD. 11 992 lesions referred.	11.7% were MM.			MM number needed to treat: 8.5
		PCP with special interest in skin cancer	1942 lesions referred.	10.6% MM.			MM number needed to treat: 9.4
		PCPs	1942 lesions referred.	5.9% MM.			MM number needed to treat: 17.0
		High dermoscopy use	17 917 lesions referred.	11.2% MM.			MM number needed to treat: 8.9
		Medium use	2657 lesions referred.	9.1% MM.			MM number needed to treat: 10.9
		Low use	1093 lesions referred.	6.9% MM.			MM number needed to treat: 14.6 (p<0.0001, but NS when adjusted for subspecialisation)
Secker *et al* 44	Sequential design DA study comparing performance of 293 PCPs in diagnosing PSLs before and after a training intervention.	Pretest (clinical images, no education) (20)		20 PSL images in test set: 3 MM, 2 BCC, 15 benign.	For MM: Sens 0.49%, spec 0.75%		% correct treatment: (1) malignant 85.87, (2) naevi 92.83, (3) benign 9.56
		Post-test (clinical images with education) (20)			Sens 0.50%, spec 0.77%		(1) malignant 84.03, (2) naevi 95.61, (3) benign 7.81
		Integrated post-test (clinical and dermoscopic images with education) (20)			Sens 0.66%, spec 0.70%		(1) malignant 91.74, (2) naevi 92.35, (3) benign 29.35
		Overall			Training improved sens and spec for all except pigmented naevi. Training improved DA for all PSLs except naevi.		Increase in correct treatments for benign lesions, reduced unnecessary referrals and excisions.
Westerhoff *et al* 33	Intervention study assessing performance of 74 PCPs with no dermoscopy experience in diagnosing 100 PSLs using macroscopic images ± dermoscopy images before and after an educational intervention.	Control: no education (100)		100 images of lesions used: 50 invasive MMs and 50 non-melanomas.	Macroscopic images: Pretest: Sens 50.6%, spec 55.2% Post-test: Sens 53.7%, spec 51.5%		Significant improvement with training between pretest (54.6%) and post-test (62.7%) (p=0.007) on macro images. Diagnosis of MM with dermoscopy significantly better (75.9%) than macro images (62.7%)(p=0.000007). No significant difference adding dermoscopic images in diagnosing non-MM PSLs.
					+Dermoscopic images: Pretest: Sens 52.9%, spec 58.1% Post-test: Sens 54.8%, spec 55.8%		
		Intervention: with education (100)			Macroscopic images: Pretest: Sens 54.6%, spec 53.0% Post-test: Sens 62.7%, spec 53.6%		
					+Dermoscopic images: Pretest: Sens 57.8%, spec 55.5% Post-test: Sens 75.9%, spec 57.8%		
Börve *et al* 34	Case–control study. Smartphone TDS system in 20 primary healthcare centres compared with traditional, paper-based referral system from other primary healthcare centres.	Control: paper-based referrals (746)	746 suspicious lesions referred.	323 malignant (13 MM, 7 MM in situ, 22 SCCs, 115 BCCs, 164 AKs), 423 benign.			3/4 invasive MMs given medium/low priority, 3/5 MMs in situ given low priority. Mean response time 5 days (range 0–82 days). Patients received primary treatment on single face-to-face visit in 82.2% of cases.
		Intervention: smartphone TDS referrals (816)	816 suspicious lesions referred. 346 (42%) non-suspicious, final diagnosis benign for 343 (3 malignant lesions missed were AKs). 196 deemed malignant, 146 (74%) also malignant after dermatology/HP.	229 malignant (19 MM, 16 MM in situ, 24 SCCs, 109 BCCs, 61 AKs), 587 benign.			All invasive MMs prioritised correctly (high), all MM in situ at least medium priority. 22.6% more referrals given low priority. Mean response time 109 min (range 2 min to 46 hours). Waiting time for surgical treatment for MM significantly shorter (p<0.0001). Patients received primary treatment on single face-to-face visit in 93.4% of cases.
Grimaldi *et al* 35	Sequential design DA study assessing PCP diagnosis of suspicious PSLs before and after dermoscopic evaluation and accuracy of teledermatology triage system.	PCP clinical (235)	167 lesions non-suspicious, 68 suspicious.	16 malignant (5 MMs), 219 benign.	Dermoscopy by PCPs and then experts led to 76.5% reduction in number of surgical procedures (68 to 16).		PCP clinical vs PCP dermoscopy: p<0.001, OR 0.345731
		PCP dermoscopy (235)	206 non-suspicious, 29 suspicious (dermoscopy-corrected diagnosis in 57.3% of cases, only 1 false negative).				PCP clinical vs expert dermoscopy: p<0.001, OR 0.179425
		TDS (235)	219 non-suspicious, 16 suspicious (1 false negative from 206 benign lesions).				PCP dermoscopy vs expert dermoscopy: p<0.05, OR 0.518973
Livingstone and Solomon^41^	Prospective case series to assess cost-effectiveness, accuracy and patient satisfaction of a TDS system for non-malignant PSLs in a primary care practice.	(248)	248 patients that PCP would have been referred routinely to dermatology referred to TDS service. 102 needed face-to-face dermatology review. 146 advised on treatment. 3 lesions possibly malignant so referred 2-week-wait pathway.	0/3 possibly malignant lesions were malignant at face-to-face review. None of other 245 lesions were malignant after review or follow-up.			Waiting time for images to be taken (weeks): 0–1=27%, 1–2=45%, 2–3=7%, 3–4=0%, 4–5=7%, 5–6=7%. Waiting time for results (weeks): 0–1=14%, 1–2=61%, 2–3=14%, 3–4=7%. 129 patients returned patient satisfaction questionnaires. 100% said TDS service was explained, 100% would recommend TDS.
Moreno-Ramirez *et al* 36	Sequential design DA study to assess if teledermatology with dermoscopy images would improve the current teledermatology-based triage system in referrals from a primary care centre.	Control: teledermatology referrals (61)	4 BCCs, 1 MM, 0 dysplastic naevus, 56 benign. Referral rates 47.5% (29). Rate of referral of true positive results 17.2% (5 true positives/29 referrals). False positives 58.7% (17/29 referrals).	HP: 2 BCCs, 1 MM, 1 dysplastic naevus, 57 benign.	Sens 1 (as 0 false negative), spec 0.65, false positive rate 0.35		Clinical picture quality excellent 41%, poor 3.3%. Average diagnostic confidence 4.14/5. Agreement with histology 0.91.
		Intervention: TDS referrals (61)	2 BCCs, 1 MM, 1 dysplastic naevus, 54 benign. Referral rates 39.3% (24) (p<0.05). Rate of referral of true positive results 20.8% (5 true positives/24 referrals) (p<0.05). False positives 41.7% (10/24) (p<0.05).		Sens 1 (as 0 false negatives), spec 0.78 (p<0.05), false positive rate 0.22 (p<0.05)		Dermoscopic picture quality excellent 63.9%, poor 6.6%. Average diagnostic confidence 4.75/5 (p<0.05). Agreement with histology 0.94.
van der Heijden *et al* 37	Cohort study assessing accuracy and reliability of TDS diagnosis with images taken by PCPs compared with diagnosis at face-to-face consultations for same lesions.	Control: face-to-face assessment (76)	All 108 lesions also referred for face-to-face assessment. 76 lesions seen face-to-face by dermatology. 32 not seen as did not attend, moved away, GP did excision.				Agreement face-to-face vs HP diagnosis k=0.90 (almost perfect), diagnostic agreement k=0.56–0.78 (substantial), management agreement k=0.31–0.38 (fair).
							Agreement TDS vs face-to-face diagnosis k=0.55–0.73 (moderate-substantial), TDS vs face-to-face management k=0.19–0.29 (fair). Image quality: 36% bad, 36% good. TDS consultations with good image quality had better agreement, TDS vs face-to-face diagnosis (k=053–0.77, substantial), and TDS vs face-to-face management (k=0.34–0.47, fair-moderate).
		Intervention: TDS referrals (108)	108 lesions referred via TDS.	HP diagnosis for 36 lesions (33%). 2 MMs and 5 non-melanoma skin cancers.			Agreement TDS vs HP diagnosis k=0.41–0.63 (moderate). TDS consultations with good image quality had better agreement of TDS vs HP diagnosis (k=0.53–1.0, moderate-almost perfect).

**Table 3C:** Summarised results of the included survey-based studies

Study	Summary	Population studied (N)	Outcomes
Survey-based studies			
Chappuis *et al* 38	Survey of PCPs in 3 regions of France.	PCPs (425)	Among dermoscopy users, 21 (54%) had no training, 8 (21%) trained via books, 5 (13%) trained with dermatologist, 2 (5%) trained online. Lower referral rates in dermoscopy group. Male PCPs significantly more likely to use a dermatoscope (p=0.001). PCPs >50 significantly more likely to use a dermoscopy (p<0.001). 30 (8%) had dermatoscope available, 16 (52%) used it >1×/week.
Morris *et al* 39	Descriptive cross-sectional survey of US physicians (medical doctors and doctors of osteopathy) to examine dermoscopy use and barriers.	Family physicians (705)	Confidence recognising malignant lesions: not confident=2.1%, a little confident=18.8%, moderate=21.9%, confident=47.7%, very confident=9.4%. Currently using a dermatoscope associated with seeing >400 patients/month and >60 years. Number of patients per month with suspicious lesions: <1.5 lesions=12.2%, 1.5–4.99 lesions=19.6%, 5–9.99 lesions=19.2%, 10–19.99 lesions=23.1%, >20 lesions=26%. Used a dermatoscope=19.5%. Currently use a dermatoscope=8.3%. Intention to start using in 12 months=63.6%.
Morris *et al* 40	Same study but including all clinicians.	Physicians (1466)	211 (14.6%) had used dermoscopy, 87 (6.0% of 1445) currently using, 656 (51.8%) intended to use in next 12 months. Use of and intention to use dermoscopy were associated with graduating recently, being a family physician, seeing a higher number of patients with cancer and being more confident differentiating malignant and benign skin lesions.
Stratton and Loescher^42^	Online survey, acceptance of mobile teledermoscopy by nurse PCPs in Arizona, USA.	Nurse practitioners (62)	Practitioners 40–60 years and been in practice for 1–15 years scored higher for intention to use mobile teledermoscopy. Few nurse practitioners used mobile teledermoscopy. They scored highly for perceiving that mobile teledermoscopy would have a positive impact on their practice, they would find it interesting to use, they could easily learn mobile teledermoscopy, it would help with rapid diagnosis of skin cancer and would improve the diagnosis of their patients, and they would use mobile teledermoscopy if they received training.

ABCD, Area, Border, Colour, Diameter; AC Rule, Asymmetry, Colour variation; AK, actinic keratosis; AUC, area under the curve; BCC, basal cell carcinoma; BLINCK, Benign, Lonely, Irregular, Nervous, Change, Known clues; DA, diagnostic accuracy; GP, general practitioner; HP, histopathology; MM, malignant melanoma; NPV, negative predictive value; NS, not significant; OR, Odds Ratio; 3PCL, 3-point checklist;PCP, primary care physician; PPV, positive predictive value; PSL, pigmented skin lesion; RCT, randomised controlled trial; SCARD, Skin Cancer Audit Research Database; SCC, squamous cell carcinoma; SDDI, short-term sequential digital dermoscopy imaging; SIT, sequential intervention trial; TADA, triage amalgamated dermoscopic algorithm; TDS, teledermoscopy; malig, malignant; sens, sensitivity; spec, specificity; susp, suspicious.

Most of the studies were non-RCT diagnostic accuracy studies. These showed increased diagnostic accuracy with the use of dermoscopy in primary care^28 33 35 43 44^ or in teledermoscopy-based referral systems.^34–37^ Some studies suggested this was due to improved ability to identify benign lesions when using a dermatoscope.^27 31 32 44^ All studies that assessed the effect of training found that it improved diagnostic accuracy compared with minimal or no training.^25 29–33 44^ There was evidence that use of dermoscopy without training displayed similar diagnostic accuracy to naked-eye examination.^33^ Menzies *et al* 29 showed that a dermoscopy-related technology, SolarScan, had higher sensitivity than PCPs, although this was a non-significant finding.

Table 4 summarises findings from the studies which investigated barriers and facilitators to implementing dermoscopy in primary care. Training requirements, cost of equipment and the time taken to perform dermoscopy were the most important barriers identified from the studies. However, for each barrier there were some papers that described it as a facilitator instead. Three papers performed cost-effective analyses of dermoscopy^24^ and teledermoscopy,^26 41^ and none found a significant cost-effective advantage. The main facilitators identified to the use of dermoscopy in primary care were reduced referrals, early detection of melanoma, and reduced patient and physician anxiety.

**Table 4:** Barriers and facilitators to implementation of dermoscopy and teledermoscopy

Aspect	Quoted as barrier in:	Type of study	Quoted as facilitator in:	Type of study
Training requirements	Chappuis *et al* 38	Survey	Pagnanelli *et al* 30	DA study
	Morris *et al* 40	Survey		
	van der Heijden *et al* 37	Cohort study		
Cost*	Chappuis *et al* 38	Survey	Koelink *et al* 24*	RCT
	Morris *et al* 39	Survey	Rosendahl *et al* 25	Cohort study
	Moreno-Ramirez *et al* 36	DA study	Ferrándiz *et al* 26*	RCT
			Livingstone and Solomon^41^*	Case series
Time consumption	Chappuis *et al* 38	Survey	Börve *et al* 34	Case–control
	Moreno-Ramirez *et al* 36	DA study		
	van der Heijden *et al* 37	Cohort study		
Reimbursement for offering dermoscopy services (in USA)	Morris *et al* 39	Survey		
Equipment issues	van der Heijden *et al* 37	Cohort study	Börve *et al* 34	Case–control
			Moreno-Ramirez *et al* 36	DA study
Reduced referrals			Chappuis *et al* 38	Survey
			Koelink *et al* 24	RCT
			Börve *et al* 34	DA study
			Moreno-Ramirez *et al* 36	DA study
Early detection of melanoma			Chappuis *et al* 38	Survey
Reduced patient anxiety			Chappuis *et al* 38	Survey
Reduced physician anxiety			Chappuis *et al* 38	Survey
			Moreno-Ramirez *et al* 36	DA study
			Menzies *et al* 14	DA study

*Based on studies where a cost-effective analysis was undertaken.

DA, diagnostic accuracy; RCT, randomised controlled trial.

### Patient and PCP attitudes and acceptance of dermoscopy

Several papers assessed PCP attitudes to dermoscopy through questionnaires. Stratton and Loescher^42^ found that nurse practitioners in the USA did not widely use dermatoscopes; however, they thought that dermoscopy would have a positive impact and would be willing to use mobile teledermoscopy if they received training. Morris *et al* 39 40 found that dermoscopy use among US physicians and doctors of osteopathic medicine was associated with seeing higher numbers of patients and with higher confidence in diagnosing skin lesions. Chappuis *et al* 38 found that dermoscopy use among French general practitioners (GPs) was associated with being older and male, and that only 8% of respondents had access to a dermatoscope. Livingstone and Solomon’s^41^ survey was the only one that assessed patient acceptability; they reported that 97% of patients from one general practice in Greater London were satisfied with the teledermoscopy service and 100% would recommend it.

## Discussion

### Principal findings

Only a small number of studies have examined the use of dermoscopy or dermoscopy-related technologies in the primary care setting. These studies were all set in Europe, the USA and Australia, and due to their heterogeneous nature we were not able to synthesise the findings. Nevertheless, our review found that, with appropriate training, dermoscopy in primary care is more accurate than naked-eye examination, with improvements in sensitivity and specificity and number needed to excise. Furthermore, there was some evidence that teledermoscopy-based referral systems improve triage accuracy compared with paper-based or macroscopic image-based referral systems. The limited evidence did not show a significant cost-effectiveness benefit for either dermoscopy or teledermoscopy, although dermoscopy appears to lead to a reduction in unnecessary referrals and excisions. Importantly, the review also suggests that PCPs are receptive to incorporating dermoscopy into their routine practice, although they recognised ongoing implementation barriers, particularly around training, time requirements and technology costs.

### Comparison with other studies

Our review suggests that dermoscopy in primary care is more accurate than naked-eye examination, supporting the findings from a previous review of dermoscopy for melanoma detection specifically in primary care published in 2012.^45^ A recently published Cochrane review of dermoscopy for the diagnosis of melanoma has also concluded that, although data to support dermoscopy use in primary care are limited, ‘it may assist in triaging suspicious lesions for urgent referral when employed by suitably trained clinicians’.^19^ Our review also suggests that training PCPs in dermoscopy improves diagnostic accuracy. Again, this finding is supported by the recent Cochrane review which also suggests that ‘formal algorithms may be of most use for dermoscopy training purposes and for less expert observers, however reliable data comparing approaches using dermoscopy in-person are lacking’.^19^ Previous reviews have shown that using dermoscopy without training was no more accurate than naked-eye examination alone.^13 19^ However, we were not able to identify the optimal length of training needed to train PCPs to use dermoscopy accurately, although studies of the effect of training on dermatologist diagnostic performance have shown improvement after between 2 days (6 hours of training per day)^46^ and 10 weeks (comprising 6 workshops of 4–6 hours).^47^ PCPs are likely to need short training courses, preferably with regular updates, as one of the few RCTs examining the impact of dermoscopy on the management of pigmented lesions in primary care reported a high dropout rate of GPs from the 20 hours of online trainingrequired for that study.^14^

It is important to note that the performance of dermoscopy in specialist clinics is not directly translatable as evidence for the performance of dermoscopy in primary care settings. A spectrum effect or spectrum bias is often observed when tests developed in one population are then used on another population. For example, the secondary care population is a referred population and has a higher prevalence of the condition being tested than primary care populations. This means that a diagnostic test, such as dermoscopy, will perform differently in the primary care population with the lower prevalence of the condition, compared with the secondary care population.^48^ The direction of effect is not consistent across tests and conditions; hence, to establish the performance of tests among the non-referred population in primary care, they need to be evaluated in a primary care population. This review has therefore aimed to examine existing evidence for dermoscopy use in primary care settings.

Our review suggests that a range of PCPs, including nurse practitioners in the USA, and PCPs in the USA and France, hold positive views about incorporating dermoscopy into their routine practice. Evidence from Australia supports these views and demonstrates that a wide range of PCPs are able to incorporate dermoscopy into their routine clinical practice.^16^ Only a small number of cost-effectiveness studies met our review criteria. They all assessed dermoscopy and teledermoscopy from a healthcare perspective, and only reported on short-term costs resulting from dermoscopy or non-dermoscopy approaches. None reported a significant cost-effectiveness benefit for dermoscopy^24^ or teledermoscopy,^26^ in the primary care setting, although they recommended the technologies as potentially useful tools. An English RCT of another diagnostic aid (MoleMate, incorporating SIAscopy) in primary care^49^ also reported equivocal findings on cost-effectiveness, as the device, similar in accuracy to systematic application of the 7-point checklist, resulted in increased referrals from primary care.^49 50^

Interestingly, no papers reporting the use of dermoscopy smartphone applications (‘apps’) for automated diagnosis of melanoma or skin cancers in the primary care setting met the review inclusion criteria. Kassianos *et al* 51 reviewed 39 smartphone applications and found little evidence of clinical or research-based input into the design or evaluation of these apps. A recent editorial in *The* *Lancet Oncology* supported this finding,^52^ and urged caution with early adoption of new technologies that are often poorly designed and untested, stressing the need to ensure that these technologies are appropriate, cost-effective and do not compromise patient safety. Recent studies have tested the application of artificial intelligence, neural networks and machine learning to the diagnosis of skin lesions; however, they have not yet been assessed in primary care settings.

### Strengths, limitations and future research

Our review examines the evidence for dermoscopy use in the primary care setting. It builds on the recent Cochrane review which explicitly reviewed evidence only about diagnostic accuracy of dermoscopy, with and without naked-eye examination, and describes this in specialist and generalist settings.^19^ We therefore included studies with a range of methods, surveys and qualitative studies, as well as RCTs and diagnostic accuracy studies, but still only identified a relatively small number of publications. Unfortunately, we were unable to perform a meta-analysis due to the heterogeneity in study designs, settings, populations and outcomes. All the studies are from high-income countries and therefore may be less generalisable to other countries with different healthcare systems.

## Conclusions

Despite the limited evidence, this review provides moderate support for the use of dermoscopy in primary care, with the weight of the available evidence pointing to a benefit in diagnostic accuracy for managing suspicious skin lesions. Dermoscopy is acceptable to PCPs, so it could help them triage suspicious lesions for urgent referral or reassurance. However, it will be important to establish further evidence on minimum for training to reach competence, as well as the cost-effectiveness and patient acceptability of implementing dermoscopy in primary care.

## Supplementary Material

Reviewer comments

Author's manuscript
