# Supplementary material for: Dermoscopy for melanoma detection and triage in primary care: a systematic review
Source: BMJ Open. 2019 Aug 20;9(8):e027529. doi: 10.1136/bmjopen-2018-027529 (PMC6707687; doi:10.1136/bmjopen-2018-027529)
Supplement: Supplementary data [file bmjopen-2018-027529supp001.pdf]

Supporting Information

Appendix 1 – Protocol for methods

Databases

We searched the following bibliographic databases from January 1990 to December 2017 for relevant published studies:

- Medline
- The Cochrane Central Register of Controlled Trials (CENTRAL) in the Cochrane Library
- EMBASE
- CINAHL (Cumulative Index to Nursing and Allied Health Literature via EBSCO)
- SCOPUS

Inclusion and exclusion criteria

The following inclusion criteria were applied:

i. All published literature from January 1990 to December 2017
ii. Which provide evidence around test accuracy, utility, acceptability to patients and GPs, and cost-effectiveness of dermoscopy modes in primary care
iii. Including trials, diagnostic accuracy and acceptability studies

The following exclusion criteria were also applied:

i. Studies based in any clinical setting other than at the first assessment of suspicious skin lesions (i.e. primary care in the UK)
ii. Studies involving specialist clinicians (in line with the Cochrane review of dermoscopy which stated 'although primary care clinicians can in practice be specialists in skin cancer, we considered primary care physicians as generalist practitioners and dermatologists as specialists'[1].)
iii. Studies that are not primary studies

Search Strategy

An electronic literature search of Medline, EMBASE, CENTRAL, CINAHL, and SCOPUS was performed from January 1990 to December 2017 using a combination of subject headings as detailed in Appendix Two. The validity of the search strategy was assessed by cross-checking articles identified in the search against:

i. the reference lists of key review articles[1-5]
ii. papers identified by a first and last author search of six key papers in the field[6-11]

The search was then further extended by manually screening the reference lists of all included papers.

Study Selection

The study selection followed these stages:

1. The titles and abstracts of the complete list of identified papers were independently assessed by one researcher (OTJ) against the inclusion and exclusion criteria. Those studies clearly not relevant were rejected at this stage.
2. One or two other members of the research team (SH, LJ) independently assessed a random selection of 10% of the papers that have been excluded, to check agreement between the reviewers.
3. For studies where a definite decision to reject could not be made based on title and abstract alone, the full paper was obtained for detailed assessment.
4. At least two reviewers (OTJ, FW, SH, LJ, MvM) independently assessed the full-text of included articles for the possibility of inclusion in the review. Papers identified as not meeting the inclusion criteria by both researchers were excluded.

5. The researchers met regularly to discuss papers in which it is unclear whether they meet the inclusion criteria or not and any disagreements were resolved by discussion with other members of the research team.

Data Extraction

Included papers had relevant information extracted and entered into a spreadsheet, including:

1. The characteristics of the study including:
    a. Study details
    b. Location of study
    c. Study design
    d. Study population
        i. Primary care, referred, secondary care
        ii. Control comparator
        iii. Total number of benign/suspicious lesions and number of patients
        iv. Gender
        v. Age
    e. Study methods
        i. Selection of participants
        ii. Exclusions
        iii. Follow-up time
        iv. Definition of the outcome
2. The outcomes of the study in terms of:
    a. Number of lesions deemed banal and suspicious in control and intervention groups
    b. Number of excised lesions and excised benign lesions
    c. Outcome Measure: Sensitivity/specificity
    d. Outcome Measure: Positive Predictive Value/Negative Predictive Value
    e. Outcome Measure: Others (e.g. diagnostic rate of new test, accuracy index etc.)
    f. Outcome Measure: specific to melanoma
    g. Stage of melanoma at diagnosis
    h. 5-year survival rate
3. Challenges for implementation in primary care:
    a. Training
    b. Cost
    c. Availability
    d. Cost-effective analysis.

To minimise bias, data extraction was undertaken by the lead researcher (OTJ) and by at least one other researcher independently (FMW or LJ). The research team met regularly to ensure consensus of views by discussing and resolving any differences in data extraction.

Quality Assessment Framework

Quality of the studies was assessed using the Joanna Briggs Institute Critical Appraisal Tools (Joanna Briggs Institute, 2016). The aim of the quality assessment was to determine the validity of the results based on the design, methods, analysis and conclusions of each study, and to assess the relative contribution of each study to the review. Limitations were acknowledged but studies were not excluded based on the quality assessment alone.

References

1. Dinnes J, Matin RN, Moreau JF, Patel L, Chan SA, Chuchu N, et al. Tests to assist in the diagnosis of cutaneous melanoma in adults: a generic protocol. Cochrane Database of Systematic Reviews. 2015.
2. Chappuis P, Duru G, Marchal O, Girier P, Dalle S, Thomas L. Dermoscopy, a useful tool for general practitioners in melanoma screening: A nationwide survey. British Journal of Dermatology. 2016;175:744-50.
3. Chia A, Trevena L. A Systematic Review of Training to Improve Melanoma Diagnostic Skills in General Practitioners. Journal of Cancer Education. 2016;31(4):730-5.
4. Koelink CJ, Jonkman MF, Van Der Meer K, Van Der Heide WK. Examination of skin lesions for cancer: which clinical decision aids and tools are available in general practice? European Journal of Dermatology. 2014;24(3):297-304.
5. Herschorn A. Dermoscopy for melanoma detection in family practice. Canadian Family Physician. 2012;58(7):740-5.
6. Harrington E, Clyne B, Wesseling N, Sandhu H, Armstrong L, Bennett H, et al. Diagnosing malignant melanoma in ambulatory care: A systematic review of clinical prediction rules. BMJ Open. 2017;7 (3) (no pagination)(e014096).
7. Koelink C, Vermeulen K, Kollen B, Bock G, Dekker J, Jonkman M, et al. Diagnostic accuracy and cost-effectiveness of dermoscopy in primary care: a cluster randomized clinical trial. Journal of the european academy of dermatology and venereology : JEADV [Internet]. 2014; 28(11):[1442-9 pp.].
8. Walter FM, Morris HC, Humphrys E, Hall PN, Prevost AT, Burrows N, et al. Effect of adding a diagnostic aid to best practice to manage suspicious pigmented lesions in primary care: randomised controlled trial. BMJ. 2012;345:e4110.
9. Plüddemann A, Heneghan C, Thompson M, Wolstenholme J, Price CP. Dermoscopy for the diagnosis of melanoma: Primary care diagnostic technology update. British Journal of General Practice. 2011;61(587):416-7.
10. Argenziano G, Puig S, Zalaudek I, Sera F, Corona R, Alsina M, et al. Dermoscopy improves accuracy of primary care physicians to triage lesions suggestive of skin cancer. Journal of Clinical Oncology. 2006;24(12):1877-82.
11. Gerbert B, Bronstone A, Maurer T, Berger T, McPhee SJ, Caspers N. The effectiveness of an Internet-based tutorial in improving primary care physicians' skin cancer triage skills. Journal of Cancer Education. 2002;17(1):7-11.

Appendix 2 – Search Strategies

The specific strategies for Medline, CENTRAL, EMBASE, CINAHL and SCOPUS were as follows:

A. Medline

1. primary health care.mp. or exp Primary Health Care/

2. general practice.mp. or exp General Practice/

3. family practice.mp. or exp Family Practice/

4. ((family or general or community) adj2 (doctor* or medicine or medical pratitioner* or medical practice* or practice* or practitioner* or healthcare* or health care*)).mp. [mp=title, abstract, original title, name of substance word, subject heading word, keyword heading word, protocol supplementary concept word, rare disease supplementary concept word, unique identifier, synonyms]

5. ((primary or community) adj2 (care* or health care* or healthcare* or medical care* or patient care*)).mp. [mp=title, abstract, original title, name of substance word, subject heading word, keyword heading word, protocol supplementary concept word, rare disease supplementary concept word, unique identifier, synonyms]

6. exp physicians, family/ or family physician*.mp.

7. (nurse* adj2 practi*).mp. [mp=title, abstract, original title, name of substance word, subject heading word, keyword heading word, protocol supplementary concept word, rare disease supplementary concept word, unique identifier, synonyms]

8. clinician with minimal dermoscopy training.mp.

9. melanoma screening clinic*.mp.

10. non-expert*.mp.

11. generalist clinician*.mp.

12. 1 or 2 or 3 or 4 or 5 or 6 or 7 or 8 or 9 or 10 or 11

13. dermoscopy.mp. or exp Dermoscopy/

14. dermatoscopy.mp.

15. epiluminescence microscopy.mp.

16. confocal microscopy.mp. or exp Microscopy, Confocal/

17. exp Photomicrography/ or photomicrograph.mp.

18. incident light microscopy.mp.

19. surface microscopy.mp.

20. dermoscopic.mp.

21. novel technique*.mp.

22. (siascope or siascopy or siascopic).mp. [mp=title, abstract, original title, name of substance word, subject heading word, keyword heading word, protocol supplementary concept word, rare disease supplementary concept word, unique identifier, synonyms]
23. molemate.mp.
24. AI.mp.
25. artificial intelligence.mp. or exp Artificial Intelligence/
26. exp Image Processing, Computer-Assisted/ or computer assisted.mp.
27. exp Diagnosis, Computer-Assisted/ or computer aided.mp.
28. exp "Neural Networks (Computer)"/ or neural network*.mp.
29. image analysis.mp.
30. automatic classification.mp.
31. machine learning.mp. or exp Machine Learning/
32. computer vision.mp.
33. digital analysis.mp.
34. digitally analysed.mp.
35. (analysis adj5 digitally).mp. [mp=title, abstract, original title, name of substance word, subject heading word, keyword heading word, protocol supplementary concept word, rare disease supplementary concept word, unique identifier, synonyms]
36. computer diagnos*.mp.
37. exp Mobile Applications/ or mobile app*.mp.
38. smartphone app*.mp. or exp Smartphone/
39. recognition skill*.mp.
40. automatic detection.mp.
41. collective intelligence assessment*.mp.
42. analysis device*.mp.
43. aid to diagnosis.mp.
44. diagnostic aid*.mp.
45. (decision adj3 tool*).mp. [mp=title, abstract, original title, name of substance word, subject heading word, keyword heading word, protocol supplementary concept word, rare disease supplementary concept word, unique identifier, synonyms]
46. tele-dermatos*.mp. [mp=title, abstract, original title, name of substance word, subject heading word, keyword heading word, protocol supplementary concept word, rare disease supplementary concept word, unique identifier, synonyms]

47. tele-dermos*.mp. [mp=title, abstract, original title, name of substance word, subject heading word, keyword heading word, protocol supplementary concept word, rare disease supplementary concept word, unique identifier, synonyms]

48. ((dermosc* or dermatosc* or epiluminescence microscopy or surface microscopy) adj3 (image* or picture* or photo*)).mp. [mp=title, abstract, original title, name of substance word, subject heading word, keyword heading word, protocol supplementary concept word, rare disease supplementary concept word, unique identifier, synonyms]

49. 13 or 14 or 15 or 16 or 17 or 18 or 19 or 20 or 21 or 22 or 23 or 24 or 25 or 26 or 27 or 28 or 29 or 30 or 31 or 32 or 33 or 34 or 35 or 36 or 37 or 38 or 39 or 40 or 41 or 42 or 43 or 44 or 45 or 46 or 47 or 48

50. exp Melanoma/ or melanoma*.mp.

51. exp Skin Neoplasms/ or skin neoplasm*.mp.

52. pigmented nevus.mp. or exp Nevus, Pigmented/

53. (naevocarcinoma or nevocarcinoma).mp. [mp=title, abstract, original title, name of substance word, subject heading word, keyword heading word, protocol supplementary concept word, rare disease supplementary concept word, unique identifier, synonyms]

54. ((neoplasm* or cancer* or tumour*) adj3 (skin or pigment*)).mp. [mp=title, abstract, original title, name of substance word, subject heading word, keyword heading word, protocol supplementary concept word, rare disease supplementary concept word, unique identifier, synonyms]

55. ((suspicious or melanocytic or pigmented) adj3 (lesion* or mole* or naevus or nevus or naevi or nevi or skin)).mp. [mp=title, abstract, original title, name of substance word, subject heading word, keyword heading word, protocol supplementary concept word, rare disease supplementary concept word, unique identifier, synonyms]

56. skin lesion*.mp.

57. 50 or 51 or 52 or 53 or 54 or 55 or 56

58. 12 and 49 and 57

B. CENTRAL

1. "primary health care":ti,ab,kw (Word variations have been searched)

2. "general practice":ti,ab,kw (Word variations have been searched)

3. "family practice":ti,ab,kw (Word variations have been searched)

4. family physician*:ti,ab,kw (Word variations have been searched)

5. MeSH descriptor: [Primary Health Care] explode all trees

6. MeSH descriptor: [General Practice] explode all trees

7. MeSH descriptor: [Family Practice] explode all trees

8. MeSH descriptor: [Physicians, Family] explode all trees

9. ((family or general or community) near/2 (doctor* or medicine or medical practitioner* or medical practice* or practice* or practitioner* or healthcare* or health care*)):ti,ab,kw (Word variations have been searched)

10. ((primary or community) near/2 (care* or health care* of healthcare* or medical care* or patient care*)):ti,ab,kw (Word variations have been searched)

11. (nurse* near/2 practi*):ti,ab,kw (Word variations have been searched)

12. clinician with minimal dermoscopy training:ti,ab,kw (Word variations have been searched)

13. melanoma screening clinic*:ti,ab,kw (Word variations have been searched)

14. non-expert*:ti,ab,kw (Word variations have been searched)

15. generalist clinician*:ti,ab,kw (Word variations have been searched)

16. #1 or #2 or #3 or #4 or #5 or #6 or #7 or #8 or #9 or #10 or #11 or #12 or #13 or #14 or #15

17. "dermoscopy":ti,ab,kw (Word variations have been searched)

18. dermatoscopy:ti,ab,kw (Word variations have been searched)

19. epiluminescence microscopy:ti,ab,kw (Word variations have been searched)

20. confocal microscopy:ti,ab,kw (Word variations have been searched)

21. photomicrograph:ti,ab,kw (Word variations have been searched)

22. incident light microscopy:ti,ab,kw (Word variations have been searched)

23. "surface microscopy":ti,ab,kw (Word variations have been searched)

24. "dermoscopic":ti,ab,kw (Word variations have been searched)

25. novel technique*:ti,ab,kw (Word variations have been searched)

26. siascop*:ti,ab,kw (Word variations have been searched)

27. molemate:ti,ab,kw (Word variations have been searched)

28. AI:ti,ab,kw (Word variations have been searched)

29. "artificial intelligence":ti,ab,kw (Word variations have been searched)
30. "computer assisted":ti,ab,kw (Word variations have been searched)
31. "computer aided":ti,ab,kw (Word variations have been searched)
32. "neural network":ti,ab,kw (Word variations have been searched)
33. image analysis:ti,ab,kw (Word variations have been searched)
34. automatic classification:ti,ab,kw (Word variations have been searched)
35. "machine learning":ti,ab,kw (Word variations have been searched)
36. computer vision:ti,ab,kw (Word variations have been searched)
37. digital analysis:ti,ab,kw (Word variations have been searched)
38. digitally analysed:ti,ab,kw (Word variations have been searched)
39. digitally analysed:ti,ab,kw (Word variations have been searched)
40. analysis near/5 digitally:ti,ab,kw (Word variations have been searched)
41. computer diagnos*:ti,ab,kw (Word variations have been searched)
42. mobile app*:ti,ab,kw (Word variations have been searched)
43. smartphone app*:ti,ab,kw (Word variations have been searched)
44. recognition skill*:ti,ab,kw (Word variations have been searched)
45. automatic detection:ti,ab,kw (Word variations have been searched)
46. collective intelligence assessment*:ti,ab,kw (Word variations have been searched)
47. analysis device*:ti,ab,kw (Word variations have been searched)
48. aid to diagnosis:ti,ab,kw (Word variations have been searched)
49. diagnostic aid*:ti,ab,kw (Word variations have been searched)
50. decision near/3 tool*:ti,ab,kw (Word variations have been searched)
51. tele-dermatos*:ti,ab,kw (Word variations have been searched)
52. tele-dermos*:ti,ab,kw (Word variations have been searched)
53. ((dermoscopy or dermatoscopy or epiluminescence microscopy or surface microscopy or dermoscopic) near/3 (image* or picture* or photo*)):ti,ab,kw (Word variations have been searched)
54. MeSH descriptor: [Dermoscopy] explode all trees
55. MeSH descriptor: [Microscopy, Confocal] explode all trees
56. MeSH descriptor: [Photomicrography] explode all trees
57. MeSH descriptor: [Artificial Intelligence] explode all trees
58. MeSH descriptor: [Diagnosis, Computer-Assisted] explode all trees

59. MeSH descriptor: [Image Processing, Computer-Assisted] explode all trees
60. MeSH descriptor: [Neural Networks (Computer)] explode all trees
61. MeSH descriptor: [Machine Learning] explode all trees
62. MeSH descriptor: [Mobile Applications] explode all trees
63. MeSH descriptor: [Smartphone] explode all trees
64. #17 or #18 or #19 or #20 or #21 or #22 or #23 or #24 or #25 or #26 or #27 or #28 or #29 or #30 or #31 or #32 or #33 or #34 or #35 or #36 or #37 or #38 or #39 or #40 or #41 or #42 or #43 or #44 or #45 or #46 or #47 or #48 or #49 or #50 or #51 or #52 or #53 or #54 or #55 or #56 or #57 or #58 or #59 or #60 or #61 or #62 or #63
65. "skin neoplasm*":ti,ab,kw (Word variations have been searched)
66. melanoma*:ti,ab,kw (Word variations have been searched)
67. "pigmented nevus":ti,ab,kw (Word variations have been searched)
68. skin lesion*:ti,ab,kw (Word variations have been searched)
69. naevocarcinoma or nevocarcinoma:ti,ab,kw (Word variations have been searched)
70. ((neoplasm* or cancer* or tumour*) near/3 (skin or pigment*)):ti,ab,kw (Word variations have been searched)
71. ((suspicious or melanocytic or pigmented) near/3 (lesion* or mole* or naevus or nevus or naevi or nevi or skin)):ti,ab,kw (Word variations have been searched)
72. MeSH descriptor: [Melanoma] explode all trees
73. MeSH descriptor: [Skin Neoplasms] explode all trees
74. MeSH descriptor: [Nevus, Pigmented] explode all trees
75. #65 or #66 or #67 or #68 or #69 or #70 or #71 or #72 or #73 or #74
76. #16 and #64 and #75

C. EMBASE

1. exp *primary health care/
2. primary health care.ti,ab.
3. exp *general practice/
4. general practice.ti,ab.
5. family practice.ti,ab.
6. ((family or general or community) adj2 (doctor* or medicine or medical practitioner* or medical practice* or practice* or practitioner* or healthcare* or health care*)).ti,ab.
7. ((primary or community) adj2 (care* or health care* or healthcare* or medical care* or patient care*)).ti,ab.
8. family physician*.ti,ab.
9. (nurse* adj2 practi*).ti,ab.
10. clinician with minimal dermoscopy training.ti,ab.
11. melanoma screening clinic*.ti,ab.
12. non-expert*.ti,ab.
13. generalist clinician*.ti,ab.
14. 1 or 2 or 3 or 4 or 5 or 6 or 7 or 8 or 9 or 10 or 11 or 12 or 13
15. exp *epiluminescence microscopy/
16. epiluminescence microscopy.ti,ab.
17. dermoscopy.ti,ab.
18. dermatoscopy.ti,ab.
19. exp *confocal microscopy/
20. confocal microscopy.ti,ab.
21. photomicrograph*.ti,ab.
22. incident light microscopy.ti,ab.
23. surface microscopy.ti,ab.
24. dermoscopic.ti,ab.
25. novel technique*.ti,ab.
26. (siascope or siascopy or siascopic).ti,ab.
27. molemate.ti,ab.
28. AI.ti,ab.
29. exp *artificial intelligence/
30. artificial intelligence.ti,ab.
31. exp computer assisted diagnosis/
32. computer assisted.ti,ab.
33. exp computer analysis/
34. computer aided.ti,ab.
35. computer analysis.ti,ab.
36. exp *artificial neural network/
37. neural network*.ti,ab.
38. exp *image analysis/
39. image analysis.ti,ab.
40. automatic classification.ti,ab.
41. exp *machine learning/
42. machine learning.ti,ab.
43. computer vision.ti,ab.
44. digital analysis.ti,ab.
45. digitally analy*.ti,ab.
46. (analysis adj5 digitally).ti,ab.
47. computer diagnos*.ti,ab.

48. exp *mobile application/
49. mobile app*.ti,ab.
50. exp *smartphone/
51. smartphone app*.ti,ab.
52. recognition skill*.ti,ab.
53. automatic detection.ti,ab.
54. collective intelligence assessment*.ti,ab.
55. analysis device*.ti,ab.
56. aid to diagnosis.ti,ab.
57. diagnostic aid*.ti,ab.
58. (decision adj3 tool*).ti,ab.
59. tele-dermatos*.ti,ab.
60. tele-dermos*.ti,ab.
61. ((dermoscopy or dermatoscopy or epiluminescence microscopy or surface microscopy or dermoscopic) adj3 (image* or picture* or photo*)).ti,ab.
62. 15 or 16 or 17 or 18 or 19 or 20 or 21 or 22 or 23 or 24 or 25 or 26 or 27 or 28 or 29 or 30 or 31 or 32 or 33 or 34 or 35 or 36 or 37 or 38 or 39 or 40 or 41 or 42 or 43 or 44 or 45 or 46 or 47 or 48 or 49 or 50 or 51 or 52 or 53 or 54 or 55 or 56 or 57 or 58 or 59 or 60 or 61
63. exp *melanoma/ or exp *melanoma skin cancer/
64. melanoma*.ti,ab.
65. exp *skin tumor/ or exp *skin cancer/
66. skin neoplasm*.ti,ab.
67. exp *pigmented nevus/
68. pigmented naevus.ti,ab.
69. skin lesion*.ti,ab.
70. (naevocarcinoma or nevocarcinoma).ti,ab.
71. ((neoplasm* or cancer* or tumour*) adj3 (skin or pigment*)).ti,ab.
72. ((suspicious or melanocytic or pigmented) adj3 (lesion* or mole* or naevus or nevus or naevi or nevi or skin)).ti,ab.
73. 63 or 64 or 65 or 66 or 67 or 68 or 69 or 70 or 71 or 72
74. 14 and 62 and 73

D. CINAHL

66 S15 AND S56 AND S65
65 S57 OR S58 OR S59 OR S60 OR S61 OR S62 OR S63 OR S64
64 (MH "Skin Neoplasms+")
63 (MH "Melanoma+")
62 ((suspicious or melanocytic or pigment*) N3 (lesion* or mole* or naevus or nevus or naevi or nevi or skin))
61 ((tumour* or neoplasm* or cancer*) N3 (skin or pigment*))
60 naevocarcinoma or nevocarcinoma
59 skin lesion*
58 skin neoplasm*
57 melanoma*
56 S16 OR S17 OR S18 OR S19 OR S20 OR S21 OR S22 OR S23 OR S24 OR S25 OR S26 OR S27 OR S28 OR S29 OR S30 OR S31 OR S32 OR S33 OR S34 OR S35 OR S36 OR S37 OR S38 OR S39 OR S40 OR S41 OR S42 OR S43 OR S44 OR S45 OR S46 OR S47 OR S48 OR S49 OR S50 OR S51 OR S52 OR S53 OR S54 OR S55
55 (MH "Smartphone+")
54 (MH "Mobile Applications")
53 (MH "Neural Networks (Computer)")
52 (MH "Image Processing, Computer Assisted+") OR (MH "Image Interpretation, Computer Assisted+") OR (MH "Diagnosis, Computer Assisted+")
51 (MH "Artificial Intelligence+")
50 ((dermoscop* or dermatoscopy or epiluminescence microscopy or surface microscopy) N3 (image* or picture* or photo*))
49 tele-dermatos*
48 tele-dermos*
47 decision N3 tool*
46 diagnos* N4 aid*
45 analysis device*
44 collective intelligence assessment*
43 automatic detection
42 recognition skill*
41 smartphone app*
40 mobile app*
39 computer diagnos*
38 analysis N5 digital*
37 digitally analysed
36 digital analysis
35 computer vision
34 machine learning
33 automatic classification
32 image analysis
31 neural network*
30 computer aided
29 computer assisted
28 artificial intelligence
27 AI
26 molemate
25 siascop*
24 novel technique*
23 dermoscopic

22 surface microscopy
21 incident light microscopy
20 photomicrograph
19 confocal microscopy
18 epiluminescence microscopy
17 dermatoscopy
16 dermoscopy
15 S1 OR S2 OR S3 OR S4 OR S5 OR S6 OR S7 OR S8 OR S9 OR S10 OR S11 OR S12 OR S13 OR S14
14 (MH "Physicians, Family")
13 (MH "Family Practice")
12 (MH "Primary Health Care")
11 generalist clinician*
10 non-expert*
9 melanoma screening clinic*
8 clinician with minimal dermoscopy training
7 nurse* N2 practi*
6 family physician*
5 ((primary or community) N2 (care* or health care* or healthcare* or medical care* or patient care*))
4 ((family or general or community) N2 (doctor* or medicine or medical practitioner* or medical practice* or practice* or practitioner* or healthcare* or health care*))
3 family practice
2 general practice
1 primary health care

E. SCOPUS

(((TITLE-ABS-KEY(primary w/1 (health w/1 care))) OR (TITLE-ABS-KEY(general w/1 practice)) OR (TITLE-ABS-KEY(family w/1 practice)) OR (TITLE-ABS-KEY(family w/1 physician*)) OR (TITLE-ABS-KEY((family W/2 doctor*) OR (family W/2 medicine) OR (family W/2 (medical w/1 practitioner*)) OR (family W/2 (medical w/1 practice*)) OR (family W/2 practice*) OR (family W/2 practitioner*) OR (family W/2 healthcare*) OR (family W/2 (health w/1 care*)))) OR (TITLE-ABS-KEY((general W/2 doctor*) OR (general W/2 medicine) OR (general W/2 (medical w/1 practitioner*)) OR (general W/2 (medical w/1 practice*)) OR (general W/2 practice*) OR (general W/2 practitioner*) OR (general W/2 healthcare*) OR (general W/2 (health w/1 care*)))) OR (TITLE-ABS-KEY((community W/2 doctor*) OR (community W/2 medicine) OR (community W/2 (medical w/1 practitioner*)) OR (community W/2 (medical w/1 practice*)) OR (community W/2 practice*) OR (community W/2 practitioner*) OR (community W/2 healthcare*) OR (community W/2 (health w/1 care*))))) OR ((TITLE-ABS-KEY((community W/2 care*) OR (community W/2 (medical w/1 care*)) OR (community W/2 (patient w/1 care*)) OR (community W/2 healthcare*) OR (community W/2 (health w/1 care*)))) OR (TITLE-ABS-KEY((primary W/2 care*) OR (primary W/2 (medical w/1 care*)) OR (primary W/2 (patient w/1 care*)) OR (primary W/2 healthcare*) OR (primary W/2 (health w/1 care*)))) OR (TITLE-ABS-KEY(nurs* W/2 practi*)) OR (TITLE-ABS-KEY(clinician w/1 with w/1 minimal w/1 dermoscopy w/1 training)) OR (TITLE-ABS-KEY(melanoma w/1 screening w/1 clinic*)) OR (TITLE-ABS-KEY(generalist w/1 clinician*))))

AND

(((TITLE-ABS-KEY(surface w/1 microscopy)) OR (TITLE-ABS-KEY(dermoscopic)) OR (TITLE-ABS-KEY(novel w/1 technique)) OR (TITLE-ABS-KEY(siascop*)) OR (TITLE-ABS-KEY(molemate)) OR (TITLE-ABS-KEY(ai)) OR (TITLE-ABS-KEY(artificial w/1 intelligence))) OR ((TITLE-ABS-KEY(computer w/1 assisted)) OR (TITLE-ABS-KEY(computer w/1 aided)) OR (TITLE-ABS-KEY(neural w/1 network)) OR (TITLE-ABS-KEY(image w/1 analysis)) OR (TITLE-ABS-KEY(automatic w/1 classification)) OR (TITLE-ABS-KEY(machine w/1 learning)) OR (TITLE-ABS-KEY(computer w/1 vision))) OR ((TITLE-ABS-KEY(digital* W/5 analys*)) OR (TITLE-ABS-KEY(computer w/1 diagnos*)) OR (TITLE-ABS-KEY(mobile w/1 app*)) OR (TITLE-ABS-KEY(smartphone)) OR (TITLE-ABS-KEY(recognition w/1 skill*)) OR (TITLE-ABS-KEY(automatic w/1 detection)) OR (TITLE-ABS-KEY(collective w/1 intelligence AND assessment*))) OR ((TITLE-ABS-KEY(analysis w/1 device*)) OR (TITLE-ABS-KEY(diagnos* W/3 aid*)) OR (TITLE-ABS-KEY(decision W/3 aid*)) OR (TITLE-ABS-KEY(decision W/3 tool*)) OR (TITLE-ABS-KEY(tele-dermos*)) OR (TITLE-ABS-KEY(tele-dermatos*)) OR (TITLE-ABS-KEY((dermos* W/3 image*) OR (dermos* W/3 photo*) OR (dermos* W/3 picture*)))) OR ((TITLE-ABS-KEY((dermatos* W/3 image*) OR (dermatos* W/3 photo*) OR (dermatos* W/3 picture*))) OR (TITLE-ABS-KEY((epiluminescence W/4 image*) OR (epiluminescence W/4 photo*) OR (epiluminescence W/4 picture*))) OR (TITLE-ABS-KEY(((surface w/1 microscopy) W/4 image*) OR ((surface w/1 microscopy) W/4 photo*) OR ((surface w/1 microscopy) W/4 picture*)))) OR ((TITLE-ABS-KEY(dermoscopy)) OR (TITLE-ABS-KEY(dermatoscopy)) OR (TITLE-ABS-KEY(epiluminescence w/1 microscopy)) OR (TITLE-ABS-KEY(confocal w/1 microscopy)) OR (TITLE-ABS-KEY(photomicrograph)) OR (TITLE-ABS-KEY(incident w/1 light w/1 microscopy))))

AND

((TITLE-ABS-KEY(melanoma)) OR (TITLE-ABS-KEY(naevocarcinoma OR nevocarcinoma)) OR (TITLE-ABS-KEY((pigment* W/3 neoplasm*) OR (pigment* W/3 cancer*) OR (pigment* W/3 tumo*) OR (pigment* W/3 lesion*))) OR (TITLE-ABS-KEY((pigment* W/3 lesion*) OR (pigment* W/3 mole*) OR (pigment* W/3 naev*) OR (pigment* W/3 nev*) OR (pigment* W/3 skin))) OR (TITLE-ABS-KEY((melanocytic W/3 lesion*) OR (melanocytic W/3 mole*) OR (melanocytic W/3 naev*) OR (melanocytic W/3 nev*) OR (melanocytic W/3 skin))) OR (TITLE-ABS-KEY((suspicious W/3 lesion*) OR (suspicious W/3 mole*) OR (suspicious W/3 naev*) OR (suspicious W/3 nev*) OR (suspicious W/3 skin))) OR (TITLE-ABS-KEY((skin W/3 neoplasm*) OR (skin W/3 cancer*) OR (skin W/3 tumo*) OR (skin W/3 lesion*))))

Appendix 3 – Full list of studies that underwent full text review, their assessors and outcomes

Paper	Assessor					Reason for exclusion				
	OJ	LJ	SH	FW	MvM	No dermoscopy use	Not in 1ary care	Not peer reviewed / conference abstract	No primary data	Clinical Audit
Affifi *et al*. 2017 (A)	Exclude		Exclude			■				
Affifi *et al*. 2017 (B)	Exclude		Exclude			■				
Ahmadi *et al*. 2017	Include	Include								
Alonso *et al*. 2016	Exclude		Exclude					■		
Argenziano *et al*. 2006	Include		Include							
Baldi *et al*. 2010	Exclude	Exclude							■	
Bataille *et al*. 2011	Exclude	Exclude						■		
Bergman *et al*. 2008	Exclude				Exclude				■	
Bergstrom *et al*. 2012	Exclude								■	
Berryman *et al*. 2012	Exclude		Exclude			■				
Blackledge *et al*. 2009	Exclude	Exclude				■				
Borve *et al*. 2015	Include	Include								
Bourne *et al*. 2012	Include			Include						
Bowns *et al*. 2006	Exclude			Exclude			■			
Brinkhuijsen *et al*. 2008	Exclude				Exclude				■	
Brown *et al*. 2000	Exclude		Exclude						■	
Carli *et al*. 2007	Exclude		Exclude						■	
Carrara *et al*. 2007	Exclude	Exclude				■				
Chao Li *et al*. 2013	Exclude			Exclude				■		
Chappuis *et al*. 2016	Include		Include							
Chen *et al*. 2011	Exclude	Exclude		Exclude				■		
Congalton *et al*. 2015	Exclude			Exclude			■			

Cook *et al*. 2015	Exclude	Exclude								
Coras *et al*. 2003	Exclude	Exclude								
Dahlen Gyllencreutz *et al*. 2017	Exclude			Exclude						
Del Mar *et al*. 1995	Exclude		Exclude							
Dolianitis *et al*. 2005	Exclude			Exclude						
Drugge *et al*. 2010	Exclude	Exclude								
Ferrandiz *et al*. 2017	Include			Include						
Greenberg *et al*. 2012	Exclude	Exclude								
Griffiths *et al*. 2010	Exclude			Exclude						
Grimaldi *et al*. 2009	Include			Include						
Hennessy *et al*. 2010	Exclude		Exclude							
Jain *et al*. 2012	Exclude	Exclude	Exclude							
Joliffe *et al*. 2001 (A)	Exclude			Exclude						
Joliffe *et al*. 2001 (B)	Exclude			Exclude						
Kenney *et al*. 2016	Exclude			Exclude						
Kitchener *et al*. 2009	Exclude	Exclude	Exclude							
Koelink *et al*. 2014	Include	Include								
Kroemer *et al*. 2001	Exclude			Exclude						
Laheru *et al*. 2017	Exclude			Exclude						
Livingstone *et al*. 2015	Include			Include						
Lorentzen *et al*. 1999	Exclude	Exclude		Exclude						
MacKinnon *et al*. 2016	Exclude		Exclude							
Mahendran *et al*. 2005	Exclude	Exclude								
Masood *et al*. 2014 (A)	Exclude	Exclude								
Masood *et al*. 2014 (B)	Exclude		Exclude							
Masood *et al*. 2015	Exclude		Exclude							
Massone *et al*. 2014	Exclude			Exclude						
Menzies *et al*. 2005	Include	Include								
Menzies *et al*. 2009	Include		Include							

Mikolajcyk *et al*. 2017	Exclude	Exclude								
Monheit *et al*. 2011	Exclude		Exclude							
Moreno-Ramirez *et al*. 2006	Include			Include						
Moreno-Ramirez *et al*. 2007	Exclude	Exclude								
Morris *et al*. 2017 (A)	Include		Include							
Morris *et al*. 2017 (B)	Include	Include								
Morton *et al*. 2011	Exclude			Exclude						
Naka *et al*. 2017	Exclude	Exclude		Exclude						
Nelson *et al*. 2016	Exclude			Exclude						
Pagnanelli *et al*. 2003	Include		Include							
Perakis *et al*. 2014	Exclude	Exclude								
Phelan *et al*. 2008	Exclude			Exclude						
Picollo *et al*. 2000	Exclude			Exclude						
Picollo *et al*. 2002	Exclude			Exclude						
Rademaker *et al*. 2010	Exclude			Exclude						
Riding *et al*. 2016	Exclude		Exclude							
Robson *et al*. 2012*	Exclude	Exclude								
Rogers *et al*. 2016	Include			Include						
Rogers *et al*. 2017	Include			Include						
Rosendahl *et al*. 2011	Include	Include								
Rosendahl *et al*. 2012 (A)	Exclude	Exclude	Exclude							
Rosendahl *et al*. 2012 (B)	Include			Include						
Secker *et al*. 2014	Include	Include								
Shapiro *et al*. 2004	Exclude		Exclude							
Sheraz *et al*. 2011	Exclude	Exclude								
Singh *et al*. 2016	Exclude	Exclude	Exclude							
Skrovseth *et al*. 2010	Exclude	Exclude								
Smith *et al*. 2000	Exclude	Exclude								
Soyer *et al*. 2004	Exclude		Exclude	Exclude						

Stratton *et al*. 2016	Include	Include		Include						
Swanson *et al*. 2017	Exclude	Exclude								
Tan *et al*. 2010	Exclude			Exclude						
Tan *et al*. 2015	Exclude		Exclude							
Thomas *et al*. 2017	Exclude	Exclude								
Van der Heijden *et al.* 2013	Include			Include						
Wadhawan *et al*. 2011	Exclude			Exclude						
Watts *et al*. 2017	Exclude		Exclude							
Westerhoff *et al*. 2000	Include	Include								
Winkelmann *et al*. 2015 (A)	Exclude		Exclude							
Winkelmann *et al*. 2015 (B)	Exclude		Exclude							
Wray *et al*. 2015	Exclude			Exclude						
Zalaudek *et al*. 2008	Exclude			Exclude						
Zortea *et al*. 2014	Exclude		Exclude							
Zouridakis *et al*. 2015	Exclude	Exclude	Exclude							
