# Supplementary material for: Dermoscopy for melanoma detection and triage in primary care: a systematic review
Source: BMJ Open. 2019 Aug 20;9(8):e027529. doi: 10.1136/bmjopen-2018-027529 (PMC6707687; doi:10.1136/bmjopen-2018-027529)
Supplement: Author's manuscript [file bmjopen-2018-027529.draft_revisions.pdf]

BMJ Open

BMJ Open is committed to open peer review. As part of this commitment we make the peer review history of every article we publish publicly available.

When an article is published we post the peer reviewers' comments and the authors' responses online. We also post the versions of the paper that were used during peer review. These are the versions that the peer review comments apply to.

The versions of the paper that follow are the versions that were submitted during the peer review process. They are not the versions of record or the final published versions. They should not be cited or distributed as the published version of this manuscript.

BMJ Open is an open access journal and the full, final, typeset and author-corrected version of record of the manuscript is available on our site with no access controls, subscription charges or pay-per-view fees (http://bmjopen.bmj.com).

If you have any questions on BMJ Open's open peer review process please email info.bmjopen@bmj.com

BMJ Open

Dermoscopy for melanoma detection and triage in primary care: A systematic review

Journal:	*BMJ Open*
Manuscript ID	bmjopen-2018-027529
Article Type:	Research
Date Submitted by the Author:	26-Oct-2018

Complete List of Authors:	Jones, Owain; University of Cambridge Department of Public Health and Primary Care,<br>Jurascheck, Leo; University of Cambridge School of Clinical Medicine<br>van Melle, Marije; University of Cambridge Department of Public Health and Primary Care<br>Hickman, Sarah; Norfolk and Norwich University Hospitals NHS Foundation Trust<br>Burrows, Nigel; Cambridge University Hospitals NHS Foundation Trust, Addenbrooke's Hospital Department of Dermatology<br>Hall, Per; Cambridge University Hospitals NHS Foundation Trust, Addenbrooke's Hospital<br>Emery, Jon; University of Melbourne, General Practice and Primary Care Academic Centre; University of Cambridge Department of Public Health and Primary Care<br>Walter, Fiona; University of Cambridge, Dept of Public Health and Primary Care; University of Melbourne, General Practice and Primary Care Academic Centre

Keywords:	Dermoscopy, Melanoma, PRIMARY CARE, Systematic review, Skin Cancer

SCHOLARONE™
Manuscripts

Dermoscopy for melanoma detection and triage in primary care: A systematic review

Running head: Dermoscopy use in primary care: a systematic review

Manuscript word, table and figure count: 2780 words, 4 tables, 1 figure

OT Jones[1], LC Juraschek[2], MA van Melle[1], S Hickman[3], NP Burrows[4], PN Hall[4], J Emery[5,1], FM Walter[1,5]

[1]The Primary Care Unit, Department of Public Health and Primary Care, University of Cambridge, UK
[2]Clinical School, Addenbrooke's Hospital, University of Cambridge, UK
[3]Norwich and Norfolk University Hospitals NHS Foundation Trust, Norwich, NR4 7UY
[4]Cambridge University Hospitals NHS Foundation Trust, Addenbrooke's Hospital, Cambridge, UK
[5]Department of General Practice and the Centre for Cancer Research, Faculty of Medicine, Dentistry and Health Science, Victorian Comprehensive Cancer Centre, University of Melbourne, Australia

Corresponding author
Dr OT Jones, Department of Public Health and Primary Care, University of Cambridge, UK
E-mail: otj24@medschl.cam.ac.uk

For peer review only

For peer review only - http://bmjopen.bmj.com/site/about/guidelines.xhtml

Summary

Objective: Most skin lesions first present in primary care where distinguishing rare melanomas from benign lesions can be challenging. Dermoscopy improves diagnostic accuracy among specialists and is promoted for use by primary care physicians (PCPs). However, when used by untrained clinicians, accuracy may be no better than visual inspection. This study aimed to undertake a systematic review of literature reporting use of dermoscopy to triage suspicious skin lesions in primary care settings, and challenges for implementation.

Design: A systematic literature review and narrative synthesis (due to the heterogeneity of the study design, populations and outcomes, the results could not be pooled).

Data Sources: We searched Medline, Cochrane, EMBASE, CINAHL and SCOPUS bibliographic databases from 1st January 1990 to 31st December 2017, without language restrictions.

Inclusion criteria: Studies including assessment of dermoscopy accuracy, acceptability to patients and PCPs, training requirements, and cost-effectiveness of dermoscopy modes in primary care, including trials, diagnostic accuracy and acceptability studies.

Results: 23 studies met review criteria, representing 49,769 lesions and 3,708 PCPs, all from high-income countries. Dermoscopy, with appropriate training, was associated with improved diagnostic accuracy for melanoma and benign lesions, and reduced unnecessary excisions and referrals. Teledermoscopy-based referral systems improved triage accuracy. There was insufficient evidence to determine cost-effectiveness; costs, training and time requirements were considered important implementation barriers. Patient satisfaction was seldom assessed. Computer-aided dermoscopy and other technological advances have not yet been tested in primary care.

Conclusions: Dermoscopy could help PCPs triage suspicious lesions for biopsy, urgent referral or reassurance. However, it will be important to establish further evidence on minimum for training to reach competence, as well as the cost-effectiveness and patient acceptability of implementing dermoscopy in primary care.

Strengths and limitations of this study

- This study systematically reviews the published evidence for dermoscopy use by PCPs in primary care settings, including studies of acceptability and cost-effectiveness as well as diagnostic accuracy studies.
- The use of a broad search strategy across multiple databases enabled us to identify 23 studies whose findings examine dermoscopy use in primary care clinical practice.
- The included studies were of varying quality.
- Due to the heterogeneity of the included papers we were not able to undertake any meta-analysis; instead, we performed a narrative synthesis.

For peer review only - http://bmjopen.bmj.com/site/about/guidelines.xhtml

For peer review only

For peer review only - http://bmjopen.bmj.com/site/about/guidelines.xhtml

Introduction

In the UK malignant melanoma is the 5th commonest cancer.[1] Melanoma has one of the fastest rising incidence rates of any cancer; among white populations incidence has quadrupled over the last 30 years and is projected to rise by a further 7% in the UK between 2014 and 2035, this reflects increasing exposure to the main risk factor, UV radiation.[2] There were 2285 deaths from melanoma in 2016.[1]

Primary care (the first point of contact for patients in the healthcare system, usually community-based) can play an important role in improving outcomes for patients with melanoma. More accurate triage of suspicious pigmented skin lesions could lead to more prompt diagnosis of melanoma at earlier stages and improved outcomes, and reduce unnecessary biopsies or referrals. In the UK, most people diagnosed with cancer first present in primary care,[3] where primary care physicians (PCPs) need to distinguish rare melanomas from common benign lesions using clinical history taking and visual inspection, aided by checklists such as the seven-point checklist as recommended in the 2015 NICE guidelines for suspected cancer.[4] Various technologies may also have a role in assisting triage of suspicious skin lesions, including mobile phone applications,[5] reflectance confocal microscopy,[6] optical coherence tomography,[7] computer-aided diagnosis,[8] high frequency ultrasound,[9] and dermoscopy.[10]

Dermoscopy (also referred to as dermatoscopy or epiluminescence microscopy) is a non-invasive technique using a hand-held magnifier and incident light, which may be polarised to reduce reflection, to reveal subsurface structures. Dermoscopy performed by trained specialists is more sensitive and specific in classifying skin lesions than clinical examination with the naked eye alone.[4, 11] Dermatologists and some international guidelines recommend PCPs use dermoscopy;[12] however, when used by untrained or less experienced clinicians, accuracy can be no better than inspection alone,[13] and there is a danger of increased excisions, over-referral or false reassurance. It takes time to train clinicians to use dermoscopy, and PCP training dropout rates have been shown to be high.[14, 15] For these reasons dermoscopy is not currently recommended for use by PCPs in the UK,[4] although it is used routinely by PCPs in Australia,[16] which has the highest incidence of melanoma worldwide. Some digital dermoscopy devices exist, a few of which incorporate computer-aided diagnosis, but they are expensive, and, while showing better sensitivity, even in expert hands they have lower specificity than clinicians alone.[17]

A Cochrane review of dermoscopy has recently been published, and examines the diagnostic accuracy of dermoscopy, with and without visual inspection, for the detection of cutaneous invasive melanoma and intra-epidermal melanocytic variants in adults.[18] Our systematic review has a broader aim, focussing on the first presentation of suspicious skin lesions in primary care and whether dermoscopy and dermoscopy-related technologies, with suitable training, can be used accurately and effectively to triage suspicious skin lesions at this point in the healthcare pathway. We considered hand-held dermoscopy, computer-aided/digital dermoscopy devices, and included novel tele-dermoscopy approaches (i.e. referral using electronic dermoscopy images or video). Additionally, we looked for data on the practical challenges to implementing dermoscopy in primary care, including test accuracy, utility, acceptability to patients and PCPs, training requirements, and cost-effectiveness.

For peer review only - http://bmjopen.bmj.com/site/about/guidelines.xhtml

Methods

This systematic review was conducted in accordance with the PRISMA guidelines,[19] and the protocol was registered with PROSPERO (CRD42018091395) prior to conducting the review.[20] All aspects of the protocol were reviewed by senior faculty from the CanTest collaborative (www.cantest.org).

We searched the MEDLINE, EMBASE, Cochrane Central, CINAHL and SCOPUS databases using keywords related to dermoscopy, melanoma and primary care, without language restrictions, from 1st January 1990 to 31st December 2017. We also manually searched reference lists of included studies. We included all types of study as we anticipated that there would be few relevant randomised controlled trials or diagnostic accuracy studies performed in primary care, and we aimed to find additional quantitative and qualitative evidence. We chose to start the search from 1990 as this was when the earliest research around dermoscopy emerged. We also considered published evidence from international healthcare systems and whether it could be interpreted and applied to the UK, including the extent to which data collected from specialist clinic settings could be applied to the lower-prevalence primary care population.

We included all studies which provide evidence around test accuracy, utility, acceptability to patients and PCPs, training requirements, and cost-effectiveness of dermoscopy modes in primary care; including trials, diagnostic accuracy and acceptability studies. As our interest was in use of dermoscopy by generalist clinicians, we included all studies reporting PCP use of dermoscopy; studies of secondary care physicians who were not trained in dermoscopy were also included. We excluded studies that were based in any clinical setting other than at the first assessment of suspicious skin lesions (e.g. primary care in the UK), and any studies that were not considered primary studies.

Following duplicate removal, one author (OTJ) screened titles and abstracts to identify studies which fitted the inclusion criteria. 10% of the titles and abstracts were checked by two other authors (LJ and SH), and inter-assessor reliability was excellent, with disagreement for only one out of the 100 papers checked. Any disagreements were discussed by the core research team (OTJ, LJ, SH, FMW) and a consensus reached. At least two reviewers (OTJ, LJ, SH, MvM, FMW) independently assessed each full-text article for the possibility of inclusion in the review. Any disagreements were resolved by consensus-based discussion.

Data extraction was undertaken by two reviewers independently (OTJ, LJ, FMW) and summarised using descriptive tables, discussion and consensus. We chose to extract only reported outcomes from the included papers, without calculating further quantitative measures of diagnostic accuracy from their data, unless already reported. Due to the heterogeneity of the included papers we were not able to undertake any meta-analysis; instead, we chose to perform a narrative synthesis.

Risk-of-bias assessment was undertaken for each full-text paper by two independent researchers (OTJ, LJ) using the Joanna Briggs Institute Critical (JBI) Appraisal Tools.[21] These tools incorporate various critical assessments for different study designs, including patient selection, randomisation, data collection and analysis. As assessments for different study designs had varying denominators, the score

For peer review only - http://bmjopen.bmj.com/site/about/guidelines.xhtml

was converted to a percentage and classified as high, medium and low risk to aid clarity of presentation and interpretation (**Table 2**). Although the studies demonstrated a wide range in quality, no studies were excluded based upon their risk-of-bias assessment. Full details of our review question, search strategy, inclusion/exclusion criteria, methodology for data extraction, risk-of-bias assessment and outcomes extraction are described in **Appendices 1 and 2** (see Supporting Information), as well as a full list of excluded studies (**Appendix 3**).

Results

Figure 1 shows the study PRISMA diagram. 837 studies were identified, of which 349 were duplicates. 95 articles underwent full-text review and 23 met the inclusion criteria.[14, 22-43] These 23 articles reported data relating to 49,769 lesions and 3,708 PCPs.

Table 1 provides a summary of the study characteristics for included studies. We included three randomised controlled trials (RCT), two sequential intervention trials (SIT), nine diagnostic accuracy studies, two cohort studies, two case series, one case-control study, and four PCP surveys. Table 1 also visually summarises the practitioner and patient populations reported in the studies and highlights the paucity of studies reporting PCPs using dermoscopy with primary care patients (5 out of 16). Overall, 16 of the 23 papers reported studies of PCPs, but only 11 papers reported studies involving PCPs using dermoscopy with patients in consultations.

For peer review only

For peer review only - http://bmjopen.bmj.com/site/about/guidelines.xhtml

Table 1: Study demographics - patient and practitioner populations

Study details	Location	Study Type	Practitioners: PCPs	Practitioners: SCPs	Practitioners: PCPs & SCPs	Practitioners: DPCSCCs	Patients – Studies F2F with patients from: Primary care	Patients – Studies F2F with patients from: Secondary care	Patients – Studies F2F with patients from: DPCSCCs	Patients – Studies using images from: Primary care	Patients – Studies using images from: Secondary care	Patients – Studies using images from: DPCSCCs	Control group	Intervention group	Gender of patients (except where indicated) (Female [F]/ Male [M])	Age of patients (except where indicated) (mean (years), range)
**Dermoscopy papers**																
Ahmadi *et al.* 2017	Maastricht/ Limburg, Netherlands	Case series	■				■						None	Patients from 3 primary care practices	F=57.8%	54.7 60-79
Argenziano *et al.* 2006	Barcelona, Spain. Naples, Italy	RCT	■				■						Naked eye	Dermoscopy	C: F=62.4% I: F=62.3%	C: 40, 2-90 I: 41, 3-94
Bourne *et al.* 2012	Brisbane, Australia	DA study	■									■	Clinical assessment & algorithms	BLINK algorithm	F=52.2%	58 30-60
Chappuis *et al.* 2016	4 regions of France	Survey	■										N/A	PCPs in France	GPs: F=42.4%	GPs: <30=8, 30-50=169 >50yrs=246
Koelink *et al.* 2014	Groningen, Netherlands	RCT	■				■						Naked eye	Dermoscopy	C: F=61.6% I: F=68.2%	C: 54.7 I: 53.2
Menzies *et al.* 2005	US, Germany & Australia	DA study			■							■	Independent clinicians	SolarScan assessment	N/R	N/R
Menzies *et al.* 2009	Perth, Australia	SIT	■				■						PCP decision before intervention	Outcome after dermoscopy & SDDI	N/R	N/R
Morris *et al.* 2017 (a)	Florida, USA	Survey	■										None	PCPs	Clinicians: F=41.6%	Clinicians: Median 40-49yrs
Morris *et al.* 2017 (b)	Florida, USA	Survey			■								None	Practicing physicians	Clinicians: F=34.7%	Clinicians: N/R
Pagnanelli *et al.* 2003	Rome, Italy	DA study		■ *							■		Pre-training	Post-training	N/R	N/R
Rogers *et al.* 2016	New York, US	DA study			■						■		Histology/ expert opinion	Clinicians using 3 algorithms	F=53.3%	Median 31-40yrs

For peer review

Rogers *et al.* 2017	New York, US	DA study			■						■		Histology/ expert opinion	Clinicians using 3 algorithms	F=53.3%	Median 31-40yrs
Rosendahl *et al.* 2011	Queensland, Australia	SIT	**									■	Naked-eye	Dermoscopy images	F=32.6%	57 SD: 17yrs
Rosendahl *et al.* 2012	Australian SCARD database	Cohort study	■			■	■		■				Histology diagnosis	PCP decision	N/R	N/R
Secker *et al.* 2016	Leiden, Netherlands	DA study	■								■		PCPs before education	After education	F=51.8%	45.2 28-63
Westerhoff *et al.* 2000	Sydney, Australia	DA study	■								■		PCP diagnosis	PCPs +/- Dermoscopy +/- education	N/R	N/R
Teledermoscopy papers																
Borve *et al.* 2015	Gothenburg, Sweden	Case control study	■				■						Paper-based referrals	Teledermoscopy referrals	C: F=57.1% I: F=61.4%	C: 61, 18-97 I: 54, 18-93
Ferrandiz *et al.* 2017	Andalucia, Spain	RCT	■				■						Clinical images	Clinical & Dermoscopy images	C: F=52.88% I: F=62.28%	C: 57.33 I: 54.96
Grimaldi *et al.* 2009	Siena, Italy	DA study	■				■						Judgement before dermoscopy	Judgement after dermoscopy	N/R	N/R
Livingstone *et al.* 2015	Ruislip, UK	Case series	■				■						Expert Diagnosis & standard costs	Teledermoscopy referrals	N/R	N/R
Moreno-Ramirez *et al.* 2006	Sevilla, Spain	DA study	■				■						Tele-dermatology referrals	Same patients + dermoscopy images	F=70.5%	38.8 1-73
Stratton *et al.* 2016	Arizona, US	Survey			■								None	Nurse practitioners	Nurse Practitioners: F=92%	Nurse Practitioners: 48
Van der Heijden *et al.* 2013	Amsterdam, Netherlands	Cohort study	■				■						Face-to-face consult +/- histology	Teledermoscopy consult (same patients)	F=55%	Median 47yrs 6-84yrs

* = minimal dermoscopy experience although secondary care physicians. ** = practitioner population not specified, DA study = Diagnostic Accuracy study, RCT = Randomised Controlle Trial, SIT = sequential intervention trial, PCP = Primary Care Practitioner, SCP = Secondary Care Practitioner, DPCSCC = Dedicated Primary Care Skin Cancer Clinic, SDDI = short-term sequential digital dermoscopy imaging, SCARD = Skin Cancer Audit Research Database, PSL = pigmented skin lesion, C group = control group, I group = intervention group

For peer review only - http://bmjopen.bmj.com/site/about/guidelines.xhtml

Table 2 summarises the outcome measures of each included study, grouped into accuracy and reliability outcomes and implementation outcomes, and shows the heterogeneous nature of the reported outcomes. The accuracy and reliability outcomes were diverse; twelve papers reported sensitivity and specificity, eight reported diagnostic accuracy or area-under-the-curve, five reported positive and negative predictive values (PPVs and NPVs), fourteen reported the proportion of correct decisions, four reported the number needed to excise, and five reported the biopsy rate. The implementation outcomes were less numerous but also quite diverse: four papers reported on PCP opinions, three performed cost-effective analyses, two looked at response times for teledermoscopy services, two looked at image quality for teledermoscopy, and one assessed patient satisfaction.

Risk-of-bias outcomes are included in **Table 2** demonstrating a wide range in quality across the studies. No studies were excluded based upon the risk-of-bias assessment.

For peer review only

For peer review only - http://bmjopen.bmj.com/site/about/guidelines.xhtml

Table 2: Diversity of reported outcomes and critical appraisal results of the included studies

Study	Accuracy and Reliability Outcomes									Implementation Outcomes					JBI Critical Appraisal Checklists					
	Sens & Spec	DA / AUC	PPV &NPV	Correctly diagnosed lesions	NNE	Biopsy rate	Inter-observer agreement	Inter-instrument reliability	Odds Ratio/ Relative Risk	Survey/ PCPs opinion	Cost-effective analysis	Response time for TDS	Patient Satisfaction	Image quality for TDS	Diagnostic Test Accuracy Studies	RCTs	Analytical Cross-Sectional Studies	Quasi-Experimental Studies	Case Series	Cohort Studies
**Randomised Controlled Trials and Sequential Intervention Trials**																				
Argenziano *et al.* 2006	✓		✓	✓												30-60%				
Koelink *et al.* 2014		✓		✓		✓			✓		✓					30-60%				
Menzies *et al.* 2009	✓		✓	✓	✓	✓												>60%		
Rosendahl *et al.* 2011	✓	✓		✓											>60%					
Ferrandiz *et al.* 2017	✓	✓	✓	✓					✓		✓					>60%				
**Non-RCT diagnostic accuracy studies**																				
Ahmadi *et al.* 2017			✓	✓		✓													>60%	
Bourne *et al.* 2012	✓	✓		✓	✓	✓									>60%					
Menzies *et al.* 2005	✓	✓	✓	✓				✓							30-60%					
Pagnanelli *et al.* 2003	✓	✓					✓											>60%		
Rogers *et al.* 2016	✓														30-60%					
Rogers *et al.* 2017	✓			✓											30-60%					
Rosendahl *et al.* 2012					✓														>60%	
Secker *et al.* 2016	✓			✓														30-60%		
Westerhoff *et al.* 2000	✓																	>60%		
Borve *et al.* 2015				✓								✓								30-60%
Grimaldi *et al.* 2009				✓	✓	✓												>60%		
Livingstone *et al.* 2015											✓	✓	✓						30-60%	
Moreno-Ramirez *et al.* 2006	✓	✓		✓										✓				>60%		
Van der Heijden *et al.* 2013		✓		✓			✓							✓				>60%		
**Survey-based studies**																				
Chappuis *et al.* 2016										✓							>60%			
Morris *et al.* 2017 (a)										✓							<30%			
Morris *et al.* 2017 (b)										✓							<30%			
Stratton *et al.* 2016										✓							30-60%			

Sens= sensitivity, spec=specificity, DA=diagnostic accuracy, AUC=area-under-the-curve, PPV=positive predictive value, NPV=negative predictive value, NNE=number-needed-to-excise, PCP=primary care practitioner, TDS=teledermoscopy, JBI=Joanna Briggs Institute, RCTs=randomised controlled trials

Key to JBI score: >60% | 30-60% | <30%

For peer review only - http://bmjopen.bmj.com/site/about/guidelines.xhtml

Table 3 summarises the diagnostic accuracy results, with the studies grouped into RCTs and SITs, non-RCT diagnostic accuracy studies, and survey studies. Among the RCTs and SITs, Argenziano *et al*.[22], Koelink *et al.*[23], Rosendahl *et al.*[42] and Menzies *et al.*[14] found that dermoscopy reduced the Number Needed to Excise to diagnose a melanoma (NNE). Ferrandiz *et al.*[25] evaluated the impact of adding dermoscopic images to the standard teledermatology referral system and found that it improved accuracy and confidence in diagnosing skin lesions.

Most of the studies were non-RCT diagnostic accuracy studies. These showed increased diagnostic accuracy with the use of dermoscopy in primary care, [27, 32, 34, 42, 43] or in teledermoscopy-based referral systems.[33-36] Some studies suggested this was due to improved ability to identify benign lesions when using a dermatoscope.[26, 30, 31, 43] All studies that assessed the effect of training found that it improved diagnostic accuracy compared to minimal or no training.[24, 28-32, 43]. There was evidence that use of dermoscopy without training displayed similar diagnostic accuracy to naked-eye examination. [32] Menzies *et al.*[28] showed that a dermoscopy-related technology, Solarscan, had higher sensitivity than PCPs although this was a non-significant finding.

For peer review only

For peer review only - http://bmjopen.bmj.com/site/about/guidelines.xhtml

Table 3(a): Summarised results of the included Randomised Controlled Trials (RCTs) and Sequential Intervention Trials (SITs)

Study	Summary	Control (C) / Interve-ntion (I) (Number of lesions)	Outcome measures					
			Healthcare professional (HCP) diagnosis	Expert/Histopathology diagnosis		Sensitivity (sens) / Specificity (spec)	Positive predictive value (PPV) / Negative predictive value (NPV)	Others
Randomised Controlled Trials and Sequential Intervention Trials								
Argenziano *et al*. 2006	RCT in primary care comparing Primary Care Practitioners (PCPs) using naked eye observation (ABCD) to PCPs using dermoscopy (3-point checklist)	**C** (1325)	Non-susp=925<br>Susp=408	39 susp<br>46 susp	23 malig<br>30 malig	Sens 54.1%<br>Spec 71.3%	PPV 11.3%<br>NPV 95.8%	2/6 MMs (malignant melanor<br>missed
		**I** (1203)	Non-susp =824<br>Susp=379	16 susp<br>61 susp	6 malig<br>33 malig	Sens 79.2%<br>Spec 71.8%	PPV 16.1%<br>NPV 98.1%	1/6 MMs missed
Koelink *et al*. 2014	Cluster RCT in primary care comparing PCP diagnosis with naked-eye examination and dermoscopy examination	**C** (230)	Non-susp =67<br>Referred=20<br>Biopsy/excision=135	Correctly diagnosed:<br>MMs 22.2% (2/9)<br>Lesions 40.5% (90/222)				All lesions odds ratio (OR)=1.<br>Relative Risk=1.25<br>MM odds ratio=5.52
		**I** (207)	Non-susp =84<br>Referred=18<br>Biopsy/excision=92	Correctly diagnosed:<br>MMs 61.5% (8/13)<br>Lesions 50.5% (98/194)<br>3 skin cancers incorrectly treated				
Menzies *et al*. 2009	SIT using within-lesion controls in primary care assessing effect of dermoscopy & short-term sequential digital dermoscopy imaging (SDDI) on management of suspicious pigmented skin lesions (PSLs) by primary care practitioners (PCPs)	**C** (374)	374 pigmented skin lesions suspicious for referral +/- excision	42 malignant lesions<br>33 MM, 1 MM in situ		**For MM:**<br>**Naked-eye:** Sens 37.5 Spec 84.6 PPV 20.7 NPV 92.7		56.4% reduction in suspiciou<br>excised/referred. 63.5% redu<br>in benign excised PSLs. 1MM<br>situ incorrectly managed in<br>intervention group.<br>Benign:MM ratio of<br>excised/referred lesions 9.5:<br>3.7:1 (after dermoscopy) vs 3<br>(dermoscopy + SDDI) (p<0.00
		**IA** (374)	After dermoscopy<br>110 referred, 192 SDDI,<br>72 observed for change			**Dermoscopy:** Sens 53.1 Spec 89.0 PPV 34 NPV 94.7		
		**IB** (192)	Of 192 SDDI: 46 referred/excised, 6 continued SDDI (2 subsequently referred, 4 observed),<br>140 observed (5 subsequently referred, 135 observed)			**+ SDDI:** Sens 71.9 Spec 86.6 PPV 36.4 NPV 96.6		
						**Overall:**<br>- Increased sens for all malignancies (40 to 67.5%, p=0.014) and MM (37.5 to		

For peer review only - http://bmjopen.bmj.com/site/about/guidelines.xhtml

					71.9%, p=0.006) following intervention<br>- Increased PPV for MM (20.7 to 36.4%, p=0.055) and NPV for MM (92.7 to 96.6%, p=0.041)		
Rosendahl et al. 2011	SIT using within lesion controls. Comparison of PSL diagnosis of 'blinded observers' using macroscopic images then dermoscopy images	**C**<br>(463)	Single best diagnosis matched histopathology diagnosis in 320 cases (69.1%)	29 MMs<br>72 BCCs<br>5 SCCs<br>NB: all PSLs excised	to achieve 80% spec, sensitivity 70.5%		AUC 0.83 [malignant neoplas<br>AUC 0.71 [melanocytic lesion
		**I**<br>(463)	Single best diagnosis matched histopathology diagnosis in 375 cases (80.1%) [P<0.001]		to achieve 80% spec, sensitivity 82.6% (N.S.)		AUC 0.89 [malignant neoplas (P<0.001)<br>AUC 0.76 [melanocytic lesion (N.S.)
Ferrandiz *et al*. 2017	RCT comparing diagnostic accuracy (DA) & cost-effectiveness of clinical teleconsultations with clinical + dermoscopic teleconsultations from 5 primary care centres	**C**<br>(226)	70.36% non-susp<br>45.14% referred for face-to-face evaluation	2.77% MM<br>11.54% non-MM skin Ca	Sens 86.57<br>Spec 72.33	PPV 56.98<br>NPV 92.86<br>Accuracy index 79.20%	False Negative rate 13.43%<br>False Positive rate 22.16%<br>69.71% decisions made with higher confidence
		**I**<br>(228)	73.24% non-susp<br>20.18% referred for face-to-face evaluation (p<0.001)	2.19% MM<br>7.89% non-MM skin Ca	Sens 92.86<br>Spec 96.24	PPV 84.38<br>NPV 98.17<br>Accuracy index 94.30% (P<0.001)	False Negative rate 7.14%<br>False Positive rate 3.76%<br>78.07% decisions made with higher confidence (p=0.001)

For peer review only - http://bmjopen.bmj.com/site/about/guidelines.xhtml

Table 3(b): Summarised results of the included non-RCT diagnostic accuracy studies

Study	Summary	Intervention or group (Number of lesions)	Outcome measures				
			Healthcare (HCP) diagnosis	Expert review	Sensitivity (sens) / Specificity (spec)	Positive predictive value (PPV) / Negative predictive value (NPV)	Others
Non-RCT diagnostic accuracy studies							
Ahmadi *et al*. 2017	Retrospective cross-sectional study of medical files from 3 general practices	(580)	67 malignant, 75 pre-malignant, 399 non-suspicious, 39 unknown. 16.7% of patients referred.	151 lesions confirmed by histopathology/ dermatology: 37 BCC, 4 MMs, 1 lentigo maligna, 20 unknown	PPV: benign lesions 85.7%, pre-malignant lesions 18.2%, malignant lesions 53.8%, BCCs 53.3%, melanoma 25%		Tools used: dermoscopy [illegible] experienced PCP advice 1[illegible] biopsy 1.9%, excision 10.[illegible]
Bourne *et al*. 2012	Sequential design DA study. 4 PCPs used new BLINK dermoscopy algorithm on 50 lesion images, compared to same PCPs using 3-point checklist, Menzies, and Clinical Assessment on same 50 images	**BLINK** (200)	Found 33/36 MMs Biopsied 131/200 lesions	Images of 50 lesions used: 1 invasive MM (0.52mm), 8 in situ	Sens 90.8% Spec 50%		Diagnostic Accuracy 65.5[illegible] Number-needed-to-excis[illegible]
		**3PCL** (200)	Found 19/36 MMs Biopsied 105/200 lesions		Sens 59.4% Spec 42.2%		Diagnostic Accuracy 48.3[illegible] Number-needed-to-excis[illegible]
		**Menzies** (200)	Found 16/36 MMs Biopsied 71/200 lesions		Sens 54.7% Spec 69%		Diagnostic Accuracy 63.9[illegible] Number-needed-to-excis[illegible]
		**Clinical** (200)	Found 9/36 MMs Biopsied 74/200 lesions		Sens 52.6% Spec 74.8%		Diagnostic Accuracy 65% Number-needed-to-excis[illegible]
Menzies *et al*. 2005	Sequential design DA study comparing the performance of SolarScan to that of clinicians with varying dermatology experience on 78 images of pigmented	**Dermatolo-gist** (78)	10.5		Diagnosing MM: Sens 81 Spec 60 PPV 30 NPV 94. Decision to excise: Sens 79 Spec 60 PPV 29 NPV 93		
		**PCP** (78)	8		Diagnosing MM: Sens 62 Spec 63 PPV 26 NPV 89. Decision to excise - Sens 62 Spec 61 PPV 25 NPV 89		

	skin lesions	**SolarScan** (78)	11.1		Diagnosing MM - Sens 85 Spec 65 PPV 32 NPV 96. Decision to excise - Sens 85 Spec 65 PPV 32 NPV 96	SolarScan's sensitivity wa[illegible] higher than PCPs but N.S.
Pagnanelli *et al*. 2003	Sequential design DA study to assess if internet-based course suitable to teach dermoscopy to 16 clinicians with minimal dermoscopy experience looking at 20 images of PSLs	**Control** Pre-training (20)		20 pigmented skin lesions in test set: 6MMs, 14 non-MMs	**Pattern analysis:** Sens 67.7, Spec 76.3	Diagnostic accuracy 49.8 k-intraobserver agreement [illegible]
					**ABCD:** Sens 58, Spec 73.4	Diagnostic accuracy 38.8 k-intraobserver agreement [illegible]
					**7-point checklist:** Sens 100, Spec 67.5	Diagnostic accuracy 60.2 k-intraobserver agreement [illegible]
					**Menzies method:** Sens 80.4, Spec 72	Diagnostic accuracy 53.5 k-intraobserver agreement [illegible]
		**Intervention** Post-training (20)			**Pattern analysis:** Sens 82, Spec 78.5	Diagnostic accuracy 60.1 k-intraobserver agreement [illegible]
					**ABCD:** Sens 78.4, Spec 79.6	Diagnostic accuracy 56.6 k-intraobserver agreement [illegible]
					**7-point checklist:** Sens 100, Spec 69.8	Diagnostic accuracy 64.5 k-intraobserver agreement [illegible]
					**Menzies method:** Sens 93.4, Spec 76	Diagnostic accuracy 62.8 k-intraobserver agreement [illegible]
Rogers *et al*. 2016	Sequential design DA study examining performance of new Triage Amalgamated Dermoscopic Algorithm (TADA) when used by 120 clinicians of various specialties looking at 50 pigmented skin lesion images	(50)	5641 lesion evaluations performed: 3034 malignant, 2607 non-suspicious	50 lesion images in test set, 23 benign, 27 malignant. Sens & Spec calculated for malignant lesions	Dermatologists: Sens 94.8%, Spec 78.5%	
					Non-dermatologists: Sens 93.7%, Spec 72.1%	
					>1yr dermoscopy experience: Sens 95.4%, Spec 77.3%	
					<1yr dermoscopy experience: Sens 91.3% Spec 74.2%	
Rogers *et al*. 2017	Sequential design DA study comparing performance of new Triage Amalgamated Dermoscopic Algorithm (TADA) to existing dermoscopy algorithms when used by 120 clinicians of	(50)	No data for 3-point checklist and AC rule. TADA: 5646 lesions evaluated. 2056 deemed non-suspicious [1891 True negatives (92.0%), 165 False negatives (8.0%)] 3590 deemed malignant [2871 True positives (80.0%), 719 False positives (20.0%)]	50 lesion images in test set, 23 benign, 27 malignant. Sens & Spec based on 40 non-pigmented skin lesions	TADA: Sens 94, Spec 75.5	Sens for MM with TADA [illegible] Spec for untrained clinicia[illegible] for benign pigmented ski[illegible] lesions using TADA 76% - (beginners can be quickly trained to identify benign lesions)
					AC: Sens 88.6, Spec 78.7	
					3-point checklist: Sens 71.9, Spec 81.4	
					Untrained (using TADA): Sens 93.6, Spec 69	
					Trained (using TADA): Sens 95.4, Spec 73.2	

For peer review only - http://bmjopen.bmj.com/site/about/guidelines.xhtml

	various specialties					
Rosendahl *et al*. 2012	Prospective cohort study using Skin Cancer Audit Research Database (SCARD) to assess impact of dermoscopy use and subspecialisation on Malignant Melanoma diagnosis by PCPs	**Dedicated Skin Cancer Practitioners**	No of lesions seen not recorded on SCARD<br>11,992 lesions referred	11.7% were MM		MM number needed to tr<br>8.5
		**PCP with Special Interest in Skin Cancer**	1942 lesions referred	10.6% MM		MM number needed to tr<br>9.4
		**PCPs**	1942 lesions referred	5.9% MM		MM number needed to tr<br>17.0
		**High dermoscopy use**	17,917 lesions referred	11.2% MM		MM number needed to tr<br>8.9
		**Medium use**	2657 lesions referred	9.1% MM		MM number needed to tr<br>10.9
		**Low use**	1093 lesions referred	6.9% MM		MM number needed to tr<br>14.6<br>(P<0.0001, but N.S. when<br>adjusted for subspecialisa
Secker *et al*. 2016	Sequential design DA study comparing performance of 293 PCPs in diagnosing pigmented skin lesions before and after a training intervention	Pre-test (clinical images, no education) (20)		20 pigmented skin lesion images in test set: 3MM, 2BCC, 15 benign	For MM:<br>Sens 0.49, Spec 0.75	% correct treatment:<br>i) malignant 85.87<br>ii) naevi 92.83<br>iii) benign 9.56
		Post-test (clinical images with education) (20)			Sens 0.50, Spec 0.77	i) malignant 84.03<br>ii) naevi 95.61<br>iii) benign 7.81
		Integrated Post-test (clinical and dermoscopic images with education) (20)			Sens 0.66, Spec 0.70	i) malignant 91.74<br>ii) naevi 92.35<br>iii) benign 29.35
		Overall			Training improved sens & spec for all except pigmented naevi. Training	Increase in correct treatm<br>for benign lesions, reduce

For peer review only - http://bmjopen.bmj.com/site/about/guidelines.xhtml

					improved diagnostic accuracy for all pigmented skin lesions except naevi	unnecessary referrals & excisions
Westerhoff *et al*. 2000	Intervention study assessing performance of 74 PCPs with no dermoscopy experience in diagnosing 100 pigmented skin lesions using macroscopic images +/- dermoscopy images before and after an educational intervention	**Control**<br>No education (100)		100 images of lesions used: 50 invasive MMs and 50 non-melanomas	**Macroscopic images**:<br>Pre-test:<br>Sens 50.6%, Spec 55.2%<br>Post-test:<br>Sens 53.7%, Spec 51.5%<br>**+ dermoscopic images:**<br>Pre-test:<br>Sens 52.9%, Spec 58.1%<br>Post-test:<br>Sens 54.8%, Spec 55.8%	Significant improvement training between pre-test (54.6%) and post-test (62 (P=0.007) on macro imag Diagnosis of malignant melanoma with dermosc significantly better (75.9 than macro images (62.7 [P=0.000007]. No significa difference adding dermos images in diagnosing non malignant melanoma pigmented skin lesions
		**Intervention**<br>With education (100)			**Macroscopic images:**<br>Pre-test:<br>Sens 54.6%, Spec 53.0%<br>Post-test:<br>Sens 62.7%, Spec 53.6%<br>**+ dermoscopic images:**<br>Pre-test:<br>Sens 57.8%, Spec 55.5%<br>Post-test:<br>Sens 75.9%, Spec 57.8%	
Borve *et al*. 2015	Case-control study. Smartphone Tele-dermoscopy system in 20 primary healthcare centres compared to traditional, paper-based referral system from other primary healthcare centres	**Control**<br>Paper-based referrals (746)	746 suspicious lesions referred	323 malignant (13 MM, 7 MM in situ, 22 SCCs, 115 BCCs, 164 AKs)<br>423 benign		3/4 invasive MMs given medium/low priority, 3/5 in situ given low priority. response time 5 days (ran 82 days). Patients receive primary treatment on sin face-to-face visit in 82.2% cases.
		**Intervention**<br>Smartphone Tele-dermoscopy referrals (816)	816 suspicious lesions referred. 346 (42%) non-suspicious, final diagnosis benign for 343 (3 malignant lesions missed were AKs). 196 deemed malignant, 146 (74%) also malignant after dermatology/HP	229 malignant (19MM, 16 MM in situ, 24 SCCs, 109 BCCs, 61 AKs)<br>587 benign		All invasive MMs prioritis correctly (high), all MM-in at least medium priority. more referrals given low priority. Mean response t 109 mins (range 2 mins-4 Waiting time for surgical treatment for MM signific shorter (p<0.0001). Patie

For peer review only - http://bmjopen.bmj.com/site/about/guidelines.xhtml

						received primary treatme single face-to-face visit in 93.4% of cases.
Grimaldi *et al*. 2009	Sequential design DA study assessing PCP diagnosis of suspicious pigmented skin lesions before and after dermoscopic evaluation and accuracy of tele-dermoscopy triage system.	**PCP Clinical** (235)	167 lesions non-suspicious, 68 suspicious	16 malignant (5 MMs), 219 benign.	Dermoscopy by PCPs and then experts led to 76.5% reduction in no. of surgical procedures (68 to 16)	PCP clinical vs PCP dermoscopy: P<0.001, OF 0.345731
		**PCP Dermoscopy** (235)	206 non-suspicious , 29 suspicious (dermoscopy corrected diagnosis in 57.3% of cases, only 1 False negative)			PCP clinical vs expert dermoscopy: P<0.001, OF 0.179425
		**Tele-dermoscopy** (235)	219 non-suspicious, 16 suspicious (1 False negative from 206 benign lesions)			PCP dermoscopy vs exper dermoscopy: P<0.05, OR 0.518973
Livingstone *et al*. 2015	Prospective case series to assess cost-effectiveness, accuracy, and patient satisfaction of a tele-dermoscopy system for non-malignant pigmented skin lesions in a primary care practice.	(248)	248 patients that PCP would have been referred routinely to dermatology referred to tele-dermoscopy service. 102 needed face-to-face dermatology review. 146 advised on treatment. 3 lesions possibly malignant so referred 2-week-wait pathway	0/3 possibly malignant lesions were malignant at face-to-face review. None of other 245 lesions were malignant after review or follow-up		Waiting time for images t taken (weeks): 0-1=27%, 2=45%, 2-3=7%, 3-4=0%, 5=7%, 5-6=7%. Waiting time for results (weeks): 0-1=14%, 1-2=61 3=14%, 3-4=7%. 129 patients returned pat satisfaction questionnaire 100% said tele-dermosco service was explained, 10 would recommend tele-dermoscopy
Moreno-Ramirez *et al*. 2006	Sequential design DA study to assess if Tele-dermatology with dermoscopy images would improve the current Tele-dermatology-based triage system in referrals from a primary care centre	**Control** Teledermat-ology referrals (61)	4 BCCs, 1MM, dysplastic naevus 0, benign 56 Referral rates 47.5% (29) Rate of referral of true positive results 17.2% (5 TPs/29 referrals) False positives 58.7% (17/29 referrals)	Histopathology: 2 BCCs, 1MM, 1 dysplastic naevus, benign 57	Sens 1 (as 0 FNs) Spec 0.65 False positive rate 0.35	Clinical picture quality excellent 41%, poor 3.3% Average diagnostic confic 4.14/5. Agreement with histology 0.91.
		**Intervention** Teledermos-copy referrals (61)	2 BCCs, 1MM, 1 dysplastic naevus, benign 54 Referral rates 39.3% (24) (P<0.05) Rate of referral of true positive results 20.8% (5 TPs/24 referrals) (P<0.05)		Sens 1 (as 0 False negatives) Spec 0.78 (P<0.05) False positive rate 0.22 (P<0.05)	Dermoscopic picture qual excellent 63.9%, poor 6.6 Average diagnostic confic 4.75/5 (P<0.05). Agreeme with histology 0.94.

For peer review only - http://bmjopen.bmj.com/site/about/guidelines.xhtml

			False positives 41.7% (10/24) (P<0.05)			
Van der Heijden *et al*. 2013	Cohort study assessing accuracy and reliability of tele-dermoscopy diagnosis with images taken by PCPs compared to diagnosis at face-to-face consultations for same lesions	**Control** Face to face assessment (76)	All 108 lesions also referred for face-to-face (F2F) assessment. 76 lesions seen face-to-face by dermatology. 32 not seen as: did not attend, moved away, GP did excision			Agreement F2F vs HP diag k=0.90 (almost perfect), Diagnostic agreement k=0 0.78 (substantial), Management agreement k=0.31-0.38 (fair)<br>Agreement TDS vs F2F diagnosis k=0.55-0.73 (moderate-substantial), T F2F management k=0.19- (fair). Image quality: 36% 36% good. TDS consultati with good image quality h better agreement TDS vs diagnosis (k=053-0.77, substantial), and TDS vs F management (k=0.34-0.4 fair-moderate)
		**Intervention** Teledermos-copy referrals (108)	108 lesions referred via tele-dermoscopy	Histopathology diagnosis for 36 lesions (33%). 2MMs and 5 non-melanoma skin cancers		Agreement TDS vs HP dia k=0.41-0.63 (moderate). consultations with good i quality had better agreem of TDS vs HP diagnosis (k= 1.0, moderate-almost per

only

For peer review only - http://bmjopen.bmj.com/site/about/guidelines.xhtml

Table 3(c): Summarised results of the included survey-based studies			
Study	Summary	Population studied (N)	Outcomes
Survey-based studies			
Chappuis *et al*. 2016	Survey of PCPs in 3 regions of France	PCPs (425)	Amongst dermoscopy users 21 (54%) had no training, 8 (21%) trained via books, 5 (13%) trained with dermatologist (5%) trained online. Lower referral rates in dermoscopy group.<br>Male PCPs significantly more likely to use a dermatoscope (P=0.001). PCPs >50 significantly more likely to use a dermoscopy (P<0.001). 30 (8%) had dermatoscope available, 16 (52%) used it >1x/week.
Morris *et al*. 2017 (a)	Descriptive cross-sectional survey of US physicians (Medical Doctors and Doctors of Osteopathy) to examine dermoscopy use and barriers	Family physicians (705)	Confidence recognising malignant lesions: Not confident=2.1%, little confident=18.8%, moderate=21.9%, confident=47.7%, very confident=9.4%<br>Currently using a dermatoscope associated with seeing >400 patients/month & >60yrs<br>Number of patients per month with suspicious lesions: <1.5 lesions=12.2%, 1.5-4.99 lesions=19.6%, 5-9.99 lesions=19.2%, 10-19.99 lesions=23.1%, >20 lesions=26%<br>Used a dermatoscope=19.5%. Currently use a dermatoscope=8.3%. Intention to start using in 12 months=63.6%
Morris *et al*. 2017 (b)	Same study but including all clinicians	Physicians (1466)	211 (14.6%) had used dermoscopy, 87 (6.0% of 1445) currently using, 656 (51.8%) intended to use in next 12 month<br>Use of and intention to use dermoscopy were associated with graduating recently, being a family physician, seeing higher number of cancer patients and being more confident differentiating malignant and benign skin lesions.
Stratton *et al*. 2016	Online survey, acceptance of mobile tele-dermoscopy by nurse Primary care practitioners in Arizona, US	Nurse practitioners (62)	Practitioners 40-60yrs & been in practice for 1-15 years scored higher for intention to use mobile tele-dermoscopy. nurse practitioners used mobile tele-dermoscopy. They scored highly for perceiving that mobile tele-dermoscopy w have a positive impact on their practice, they would find it interesting to use, they could easily learn mobile tele-dermoscopy, it would help with rapid diagnosis of skin cancer, and would improve the diagnosis of their patients, th would use mobile tele-dermoscopy if they received training.
Legend: RCT=randomised controlled trial, 3PCL=3 point checklist, 7PCL=7 point checklist, N=no.=number, PPV=positive predictive value, NPV=negative predictive value, susp=suspicious, malig=malignant, MM=malignant melanoma, OR=odds ratio, C=control group, I=intervention group, sens=sensitivity, spec=specificity, PSL=pigmented skin lesion, SDDI=short-term sequent digital dermoscopy imaging, HP=histopathology, BCC=basal cell carcinoma, SCC=squamous cell carcinoma, AK=actinic keratosis, AUC=area under the curve, F2F=face-to-face, FP=false posit FN=false negative, TP=true positive, TN=true negative, SIT=sequential intervention trial, derm=dermatology, NNE=number needed to excise, NNT=number needed to treat, DA=diagnostic accuracy, k-IA=k-intraobserver agreement, N.S.=not significant, TD=teledermatology, MTD=mobile teledermoscopy, TDS=teledermoscopy, GP=general practitioner, PCP=primary care practitioner, HCP=healthcare practitioner, DCSP=dedicated skin cancer practitioner, GPSISC=GP with special interest in skin cancer, Ca=cancer, 2ww=two week wait or urgent referral, SCARD=skin cancer audit research database			

For peer review only - http://bmjopen.bmj.com/site/about/guidelines.xhtml

Table 4 summarises findings from the studies which investigated barriers and facilitators to implementing dermoscopy in primary care. Training requirements, cost of equipment and the time taken to perform dermoscopy were the most important barriers identified from the studies. However, for each barrier there were some papers that described it as a facilitator instead. Three papers performed cost-effective analyses of dermoscopy,[23] and teledermoscopy,[25, 40] none found a significant cost-effective advantage. The main facilitators identified to the use of dermoscopy in primary care were reduced referrals, early detection of melanoma and reduced patient and physician anxiety.

For peer review only

For peer review only - http://bmjopen.bmj.com/site/about/guidelines.xhtml

Table 4: Barriers and facilitators to implementation of dermoscopy and teledermoscopy

Aspect	Quoted as barrier in:	Type of Study	Quoted as facilitator in:	Type of Study
Training requirements	Chappuis *et al*. 2016<br>Morris *et al*. 2017 (b)<br>Van der Heijden *et al*. 2013	Survey<br>Survey<br>Cohort study	Pagnanelli *et al*. 2003	DA study
Cost<br>*=based on cost-effective analysis	Chappuis *et al*. 2016<br>Morris *et al*. 2017 (a)<br>Moreno-Ramirez *et al*. 2006	Survey<br>Survey<br>DA study	Koelink *et al*. 2014 *<br>Rosendahl *et al*. 2012<br>Ferrandiz *et al*. 2017 *<br>Livingstone *et al*. 2015 *	RCT<br>Cohort study<br>RCT<br>Case series
Time consumption	Chappuis *et al*. 2016<br>Moreno-Ramirez *et al*. 2006<br>Van der Heijden *et al*. 2013	Survey<br>DA study<br>Cohort study	Borve *et al*. 2015	Case-control
Reimbursement for offering dermoscopy services (in US)	Morris *et al*. 2017 (a)	Survey		
Equipment issues	Van der Heijden *et al*. 2013	Cohort study	Borve *et al*. 2015<br>Moreno-Ramirez *et al*. 2006	Case-control<br>DA study
Reduced referrals			Chappuis *et al*. 2016<br>Koelink *et al*. 2014<br>Borve *et al*. 2015<br>Moreno-Ramirez *et al*. 2006	Survey<br>RCT<br>DA study<br>DA study
Early detection of melanoma			Chappuis *et al*. 2016	Survey
Reduced patient anxiety			Chappuis *et al*. 2016	Survey
Reduced physician anxiety			Chappuis *et al*. 2016<br>Moreno-Ramirez *et al*. 2006<br>Menzies *et al*. 2009	Survey<br>DA study<br>DA study

Legend: DA=diagnostic accuracy, RCT=randomised controlled trial

For peer review only - http://bmjopen.bmj.com/site/about/guidelines.xhtml

Patient and PCP attitudes and acceptance of dermoscopy

Several papers assessed PCPs attitudes to dermoscopy through questionnaires. Stratton *et al.*[41] found that nurse practitioners in the US did not widely use dermatoscopes; however, they thought that dermoscopy would have a positive impact, and would be willing to use mobile teledermoscopy if they received training. Morris *et al.*[38, 39] found that dermoscopy use amongst US physicians and Doctors of Osteopathic Medicine was associated with seeing higher numbers of patients and with higher confidence in diagnosing skin lesions. Chappuis *et al.* [37] found that dermoscopy use amongst French GPs was associated with being older and male, and that only 8% of respondents had access to a dermatoscope. Livingstone *et al.'s* survey was the only one that assessed patient acceptability;[40] they reported that 97% of patients from one general practice in Greater London were satisfied with the teledermoscopy service and 100% would recommend it.

Discussion

Principal findings

Only a small number of studies have examined the use of dermoscopy or dermoscopy-related technologies in the primary care setting. These studies were all set in Europe, the US and Australia, and, due to their heterogeneous nature, we were not able to synthesise the findings. Nevertheless, our review found that, with appropriate training, dermoscopy in primary care is more accurate than naked-eye examination, with improvements in sensitivity and specificity and number needed to excise. Furthermore, there was some evidence that teledermoscopy-based referrals systems improve triage accuracy compared to paper-based or macroscopic image-based referral systems. The limited evidence did not show a significant cost-effectiveness benefit for either dermoscopy or tele-dermoscopy, although dermoscopy appears to lead to a reduction in unnecessary referrals and excisions. Importantly, the review also suggests that PCPs are receptive to incorporating dermoscopy into their routine practice, although they recognised ongoing implementation barriers, particularly around training, time requirements, and technology costs.

Comparison with other studies

Our review suggests that dermoscopy in primary care is more accurate than naked-eye examination, supporting the findings from a previous review of dermoscopy for melanoma detection specifically in primary care published in 2012.[44] A recently published Cochrane review of dermoscopy for the diagnosis of melanoma has also concluded that, although data to support dermoscopy use in primary care is limited, 'it may assist in triaging suspicious lesions for urgent referral when employed by suitably trained clinicians'.[18] Our review also suggests that training PCPs in dermoscopy improves diagnostic accuracy. Again, this finding is supported by the recent Cochrane review, which also suggests that 'formal algorithms may be of most use for dermoscopy training purposes and for less expert observers, however reliable data comparing approaches using dermoscopy in-person are lacking'.[18] Previous reviews have shown that using dermoscopy without training was no more accurate than naked-eye examination alone.[13, 18] However, we were not able to identify the optimal length of training needed to train PCPs to use dermoscopy accurately, although studies of the effect of training on dermatologist diagnostic performance have shown improvement after between 2 days (6 hours training per day),[45] and 10 weeks (comprising 6 workshops of 4-6 hours).[46] PCPs are likely to need short training courses, preferably with regular updates, as one of the few RCTs examining the impact of dermoscopy on the

For peer review only - http://bmjopen.bmj.com/site/about/guidelines.xhtml

management of pigmented lesions in primary care reported a high drop-out rate of GPs from the 20-hour online training course.[14]

It is important to note that the performance of dermoscopy in specialist clinics is not directly translatable as evidence for the performance of dermoscopy in primary care settings. Spectrum effects are often observed when tests developed using referred patients, or further along the diagnostic pathway, are applied in settings such as primary care the prevalence of the condition is lower, and participants may have had less prior testing.[47] The direction of effect is not consistent across tests and conditions, hence to establish the performance of tests among a non-referred population in primary care they need to be evaluated in the primary care setting. This review has therefore aimed to examine existing evidence for dermoscopy use in primary care settings.

Our review suggests that a range of PCPs, including nurse practitioners in the US, and PCPs in the US and France, hold positive views about incorporating dermoscopy into their routine practice. Evidence from Australia supports these views, and demonstrates that a wide range of PCPs are able to incorporate dermoscopy into their routine clinical practice.[16] Only a small number of cost-effectiveness studies met our review criteria. They all assessed dermoscopy and teledermoscopy from a healthcare perspective, and only reported on short-term costs resulting from dermoscopy or non-dermoscopy approaches. None reported a significant cost-effectiveness benefit for dermoscopy,[23] or tele-dermoscopy,[25] in the primary care setting, although they recommended the technologies as potentially useful tools. An English RCT of another diagnostic aid (MoleMate, incorporating SIAscopy) in primary care,[48] also reported equivocal findings on cost-effectiveness, as the device, similar in accuracy to systematic application of the 7-point checklist, resulted in increased referrals from primary care.[48, 49]

Interestingly, no papers reporting the use of dermoscopy smartphone applications (‘apps’) in the primary care setting met the review inclusion criteria. Kassianos *et al.*[50] reviewed 39 smartphone applications and found little evidence of clinical or research-based input into the design or evaluation of these apps. A recent editorial in Lancet Oncology supported this finding,[51] and urged caution with early adoption of new technologies that are often poorly designed and untested, stressing the need to ensure that these technologies are appropriate, cost-effective and do not compromise patient safety. Recent studies have tested the application of artificial intelligence, neural networks and machine learning to the diagnosis of skin lesions; however, they have not yet been assessed in primary care settings.

Strengths, limitations and future research

Our review examines the evidence for dermoscopy use in the primary care setting. It builds on the recent Cochrane review, which explicitly reviewed evidence only about diagnostic accuracy of dermoscopy, with and without naked eye examination, and describes this in specialist and generalist settings.[18] We therefore included studies with a range of methods, surveys and qualitative studies as well as RCTs and diagnostic accuracy studies, but still only identified a relatively small number of publications. Unfortunately, we were unable to perform a meta-analysis due to the heterogeneity in study designs, settings, populations, and outcomes. All the studies are from high-income countries, and therefore may be less generalisable to other countries with different health care systems.

Conclusions

Despite the limited evidence, this review provides moderate support for the use of dermoscopy in primary care, with the weight of the available evidence pointing to a benefit in diagnostic accuracy for managing suspicious skin lesions. Dermoscopy is acceptable to PCPs, so it could help them triage suspicious lesions for urgent referral or reassurance. However, it will be important to establish further evidence on minimum for training to reach competence, as well as the cost-effectiveness and patient acceptability of implementing dermoscopy in primary care.

For peer review only - http://bmjopen.bmj.com/site/about/guidelines.xhtml

For peer review only

For peer review only - http://bmjopen.bmj.com/site/about/guidelines.xhtml

Acknowledgements

The authors thank Isla Kuhn, Reader Services Librarian, University of Cambridge Medical Library for her help in developing the search strategy.

Funding

This research arises from the CanTest Collaborative, which is funded by Cancer Research UK [C8640/A23385]. This work was also supported by FMW's Clinician Scientist award (RG 68235) from the National Institute for Health Research (NIHR). The views expressed in this publication are those of the authors and not necessarily those of the National Health Service, the NIHR or the Department of Health. The funding sources had no role in the study design, data collection, data analysis, data interpretation, writing of the report, or in the decision to submit for publication.

Contributors

OTJ developed the protocol, completed the search, screened articles for inclusion, extracted data, synthesised the findings, interpreted the results and drafted the manuscript. LCJ screened articles for inclusion, extracted data, and critically revised the manuscript. MAvM screened articles for inclusion, extracted data, and critically revised the manuscript. SH screened articles for inclusion and critically revised the manuscript. NPB developed the protocol and critically revised the manuscript. PNH developed the protocol and critically revised the manuscript. JE developed the protocol, interpreted the results, and critically revised the manuscript. FMW developed the protocol, synthesised the findings, interpreted the results, and critically revised the manuscript. All authors approved the final version.

Competing interests

Per Hall is Clinical Advisor for Skin Cancer to Check4Cancer a company that offers teledermatoscopic analysis of pigmented skin lesions (https://www.check4cancer.com); and is a medical advisor to MedX the Canadian company with the IP for the SIAscope (www.medxhealth.com/default.aspx). No other authors have any conflicts of interest to declare.

Data sharing statement

All data are available from the reports or authors of the primary research. No additional data are available.

For peer review only

For peer review only - http://bmjopen.bmj.com/site/about/guidelines.xhtml

References

1. About melanoma | Melanoma | Cancer Research UK: Cancer Research UK; 2018 [Cancer Research UK Website]. Available from: http://www.cancerresearchuk.org/about-cancer/melanoma/about accessed 25/10/2018.

2. Mistry M, Parkin DM, Ahmad AS, et al. Cancer incidence in the United Kingdom: projections to the year 2030. *Br J Cancer* 2011;105(11):1795-803. doi: 10.1038/bjc.2011.430

3. Hiom SC. Diagnosing cancer earlier: reviewing the evidence for improving cancer survival. *Br J Cancer* 2015;112 Suppl 1:S1-5. doi: 10.1038/bjc.2015.23

4. NICE guidelines NG14 - Melanoma: assessment and management. UK: National Institute for Clinical Excellence, 2015.

5. Chuchu N, Dinnes J, Takwoingi Y, et al. Smartphone applications for the diagnosis of cutaneous melanoma in adults. *Cochrane Database of Systematic Reviews* 2018 (In Press)

6. Dinnes J, Deeks JJ, Chuchu N, et al. Reflectance confocal microscopy for the diagnosis of cutaneous melanoma in adults. *Cochrane Database of Systematic Reviews* 2018 (In Press)

7. Ferrante di Ruffano L, Dinnes J, Deeks JJ, et al. Optical coherence tomography for the diagnosis of skin cancer in adults. *Cochrane Database of Systematic Reviews* 2018 (In Press)

8. Ferrante di Ruffano L, Takwoingi Y, Dinnes J, et al. Computer assisted diagnosis techniques (dermoscopy and spectroscopy-based) for the diagnosis of skin cancer in adults. *Cochrane Database of Systematic Reviews* 2018 (In Press)

9. Dinnes J, Bamber J, Chuchu N, et al. High frequency ultrasound for the diagnosis of skin cancer in adults. *Cochrane Database of Systematic Reviews* 2018 (In Press)

10. Rosendahl C, Cameron A, McColl I, et al. Dermatoscopy in routine practice - 'Chaos and Clues'. *Australian Family Physician* 2012;41(7):482-87.

11. Argenziano G, Cerroni L, Zalaudek I, et al. Accuracy in melanoma detection: a 10-year multicenter survey. *J Am Acad Dermatol* 2012;67(1):54-9. doi: 10.1016/j.jaad.2011.07.019

12. Menzies SW, Chamberlain A, Soyer HP, et al. What is the role of dermoscopy in melanoma diagnosis? Sydney: Cancer Council Australia: Cancer Council Australia Melanoma Guidelines Working Party; 2018 [Available from: https://wiki.cancer.org.au/australia/Clinical_question:What_is_the_role_of_dermoscopy_in_melanoma_diagnosis%3F accessed 25/10/2018.

13. Vestergaard ME, Macaskill P, Holt PE, et al. Dermoscopy compared with naked eye examination for the diagnosis of primary melanoma: a meta-analysis of studies performed in a clinical setting. *Br J Dermatol* 2008;159(3):669-76. doi: 10.1111/j.1365-2133.2008.08713.x [published Online First: 2008/07/12]

14. Menzies SW, Emery J, Staples M, et al. Impact of dermoscopy and short-term sequential digital dermoscopy imaging for the management of pigmented lesions in primary care: A sequential intervention trial. *British Journal of Dermatology* 2009;161(6):1270-77.

15. Noor O, 2nd, Nanda A, Rao BK. A dermoscopy survey to assess who is using it and why it is or is not being used. *Int J Dermatol* 2009;48(9):951-2. doi: 10.1111/j.1365-4632.2009.04095.x [published Online First: 2009/08/26]

16. Chamberlain AJ, Kelly JW. Use of dermoscopy in Australia. *Medical Journal of Australia* 2007;187(4):252-53.

17. Vestergaard ME, Menzies SW. Automated diagnostic instruments for cutaneous melanoma. *Semin Cutan Med Surg* 2008;27(1):32-6. doi: 10.1016/j.sder.2008.01.001 [published Online First: 2008/05/20]

18. Dinnes J, Deeks JJ, Chuchu N, et al. Cochrane Skin Cancer Diagnostic Test Accuracy Group. Dermoscopy, with and without visual inspection, for the diagnosis of melanoma in adults. *Cochrane Database of Systematic Reviews* 2018(10) doi: 10.1002/14651858.CD011902.

For peer review only - http://bmjopen.bmj.com/site/about/guidelines.xhtml

19. Stewart DM, Larissa S, Mike C, et al. Preferred reporting items for systematic review and meta-analysis protocols (PRISMA-P) 2015 statement. *Systematic Reviews* 2015;4(1):1. doi: doi:10.1186/2046-4053-4-1

20. Jones OT, Jurasceck L, Van Melle M, et al. Can dermoscopy be used accurately and effectively in primary care to appropriately triage possible melanoma? PROSPERO: CRD42018091395; 2018 [Available from: https://www.crd.york.ac.uk/prospero/display_record.php?RecordID=91395 accessed 25/10/2018.

21. Joanna Briggs Institute UoA. Joanna Briggs Institute Critical Appraisal Tools 2018 [Available from: http://joannabriggs.org/ accessed 25/10/2018.

22. Argenziano G, Puig S, Zalaudek I, et al. Dermoscopy improves accuracy of primary care physicians to triage lesions suggestive of skin cancer. *Journal of Clinical Oncology* 2006;24(12):1877-82.

23. Koelink C, Vermeulen K, Kollen B, et al. Diagnostic accuracy and cost-effectiveness of dermoscopy in primary care: a cluster randomized clinical trial. *Journal of the european academy of dermatology and venereology : JEADV* 2014; 28(11).

24. Rosendahl C, Williams G, Eley D, et al. The impact of subspecialization and dermatoscopy use on accuracy of melanoma diagnosis among primary care doctors in Australia. *Journal of the American Academy of Dermatology* 2012;67(5):846-52.

25. Ferrandiz L, Ojeda-Vila T, Corrales A, et al. Internet-based skin cancer screening using clinical images alone or in conjunction with dermoscopic images: a randomized teledermoscopy trial. *Journal of the american academy of dermatology* 2017 (In Press).

26. Ahmadi K, Prickaerts E, Smeets JGE, et al. Current approach of skin lesions suspected of malignancy in general practice in the Netherlands: a quantitative overview. *Journal of the European Academy of Dermatology & Venereology* 2017;27:27.

27. Bourne P, Rosendahl C, Keir J, et al. BLINCK-A diagnostic algorithm for skin cancer diagnosis combining clinical features with dermatoscopy findings. *Dermatology Practical & Conceptual* 2012;2(2):202a12.

28. Menzies SW, Bischof L, Talbot H, et al. The performance of SolarScan: An automated dermoscopy image analysis instrument for the diagnosis of primary melanoma. *Archives of Dermatology* 2005;141(11):1388-96.

29. Pagnanelli G, Soyer HP, Argenziano G, et al. Diagnosis of pigmented skin lesions by dermoscopy: web-based training improves diagnostic performance of non-experts. *British Journal of Dermatology* 2003;148(4):698-702.

30. Rogers T, Marino ML, Dusza SW, et al. A clinical aid for detecting skin cancer: The triage amalgamated dermoscopic algorithm (TADA). *Journal of the American Board of Family Medicine* 2016;29(6):694-701.

31. Rogers T, Marino M, Dusza SW, et al. Triage amalgamated dermoscopic algorithm (TADA) for skin cancer screening. *Dermatology Practical & Conceptual* 2017;7(2):39-46.

32. Westerhoff K, McCarthy WH, Menzies SW. Increase in the sensitivity for melanoma diagnosis by primary care physicians using skin surface microscopy. *British Journal of Dermatology* 2000;143(5):1016-20.

33. Börve A, Dahlén Gyllencreutz J, Terstappen K, et al. Smartphone teledermoscopy referrals: A novel process for improved triage of skin cancer patients. *Acta Dermato-Venereologica* 2015;95(2):186-90. doi: 10.2340/00015555-1906

34. Grimaldi L, Silvestri A, Brandi C, et al. Digital epiluminescence dermoscopy for pigmented cutaneous lesions, primary care physicians, and telediagnosis: a useful tool? *Journal of Plastic, Reconstructive and Aesthetic Surgery* 2009;62(8):1054-58. doi: 10.1016/j.bjps.2008.01.011

35. Moreno-Ramirez D, Ferrandiz L, Galdeano R, et al. Teledermatoscopy as a triage system for pigmented lesions: a pilot study. *Clin Exp Dermatol* 2006;31(1):13-8. doi: 10.1111/j.1365-2230.2005.02000.x

36. van der Heijden JP, Thijssing L, Witkamp L, et al. Accuracy and reliability of teledermatoscopy with images taken by general practitioners during everyday practice. *Journal of Telemedicine and Telecare* 2013;19(6):320-25.

For peer review only - http://bmjopen.bmj.com/site/about/guidelines.xhtml

37. Chappuis P, Duru G, Marchal O, et al. Dermoscopy, a useful tool for general practitioners in melanoma screening: A nationwide survey. *British Journal of Dermatology* 2016;175:744-50.

38. Morris JB, Alfonso SV, Hernandez N, et al. Examining the factors associated with past and present dermoscopy use among family physicians. *Dermatology Practical & Conceptual* 2017;7(4):63-70.

39. Morris JB, Alfonso SV, Hernandez N, et al. Use of and intentions to use dermoscopy among physicians in the United States. *Dermatology Practical & Conceptual* 2017;7(2):7-16.

40. Livingstone J, Solomon J. An assessment of the cost-effectiveness, safety of referral and patient satisfaction of a general practice teledermatology service. *London Journal of Primary Care* 2015;7(2):31-35.

41. Stratton D, Loescher LJ. The acceptance of mobile teledermoscopy by primary care nurse practitioners in the state of Arizona. *Journal of the American Association of Nurse Practitioners* 2016;28(6):287-93.

42. Rosendahl C, Tschandl P, Cameron A, et al. Diagnostic accuracy of dermatoscopy for melanocytic and nonmelanocytic pigmented lesions. *Journal of the American Academy of Dermatology* 2011;64(6):1068-73.

43. Secker LJ, Buis PAJ, Bergman W, et al. Effect of a dermoscopy training course on the accuracy of primary care physicians in diagnosing pigmented lesions. *Acta Dermato-Venereologica* 2017;97(2):263-65.

44. Herschorn A. Dermoscopy for melanoma detection in family practice. *Canadian Family Physician* 2012;58(7):740-45.

45. Troyanova P. A beneficial effect of a short-term formal training course in epiluminescence microscopy on the diagnostic performance of dermatologists about cutaneous malignant melanoma. *Skin Research & Technology* 2003(9):269-73.

46. Stanganelli I, Seidenari S, Serafini M, et al. Diagnosis of pigmented skin lesions by epiluminescence microscopy: determinants of accuracy improvement in a nationwide training programme for practical dermatologists. *Public Health* 1999;113(5):237-42. doi: 10.1038/sj.ph.1900575

47. Juliet AU-S, Stephen JS, Simon JG. The spectrum effect in tests for risk prediction, screening, and diagnosis. *BMJ* 2016 doi: 10.1136/bmj.i3139

48. Wilson ECF, Emery JD, Louise Kinmonth A, et al. The cost-effectiveness of a novel siascopic diagnostic aid for the management of pigmented skin lesions in primary care: A decision-analytic model. *Value in Health* 2013;16(2):356-66.

49. Walter FM, Morris HC, Humphrys E, et al. Effect of adding a diagnostic aid to best practice to manage suspicious pigmented lesions in primary care: randomised controlled trial. *BMJ* 2012;345:e4110.

50. Kassianos AP, Emery JD, Murchie P, et al. Smartphone applications for melanoma detection by community, patient and generalist clinician users: A review. *British Journal of Dermatology* 2015;172(6):1507-18.

51. Editorial. Digital oncology apps: revolution or evolution? *Lancet Oncology* August 2018;19

For peer review only

For peer review only - http://bmjopen.bmj.com/site/about/guidelines.xhtml

Supporting Information

Appendix 1 (Protocol for methods), **Appendix 2** (Search strategies) and **Appendix 3** (Full list of studies that underwent full text review, their assessors and outcomes) are available in the attached supporting information.

Figure Legends

Figure 1: PRISMA flow diagram for the studies included in the review.

For peer review only

For peer review only - http://bmjopen.bmj.com/site/about/guidelines.xhtml

For peer review only

For peer review only - http://bmjopen.bmj.com/site/about/guidelines.xhtml

Figure 1: PRISMA flow diagram for the studies included in the review

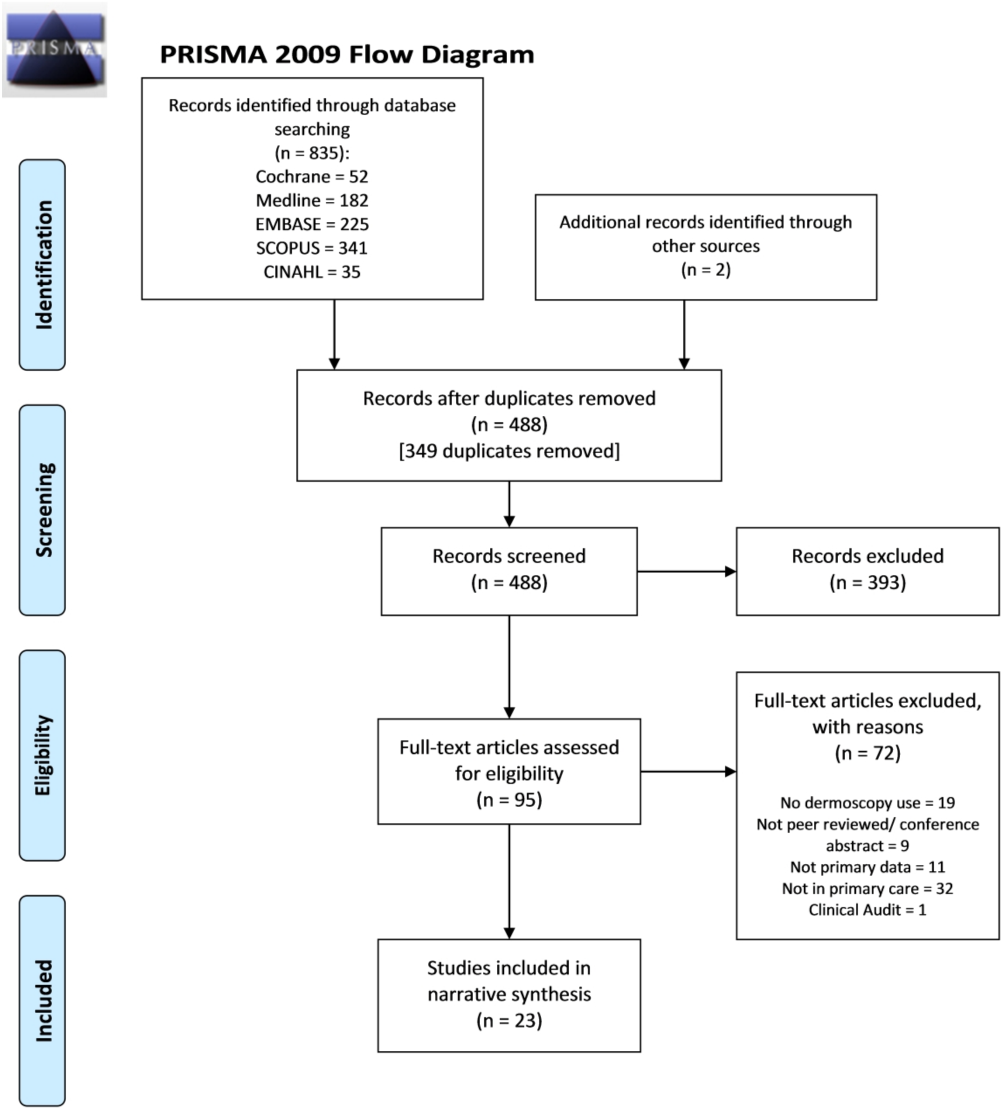

Figure 1: PRISMA flow diagram for the studies included in the review

1134x1309mm (96 x 96 DPI)

Supporting Information

Appendix 1 – Protocol for methods

Databases

We searched the following bibliographic databases from January 1990 to December 2017 for relevant published studies:

- Medline
- The Cochrane Central Register of Controlled Trials (CENTRAL) in the Cochrane Library
- EMBASE
- CINAHL (Cumulative Index to Nursing and Allied Health Literature via EBSCO)
- SCOPUS

Inclusion and exclusion criteria

The following inclusion criteria were applied:

i. All published literature from January 1990 to December 2017
ii. Which provide evidence around test accuracy, utility, acceptability to patients and GPs, and cost-effectiveness of dermoscopy modes in primary care
iii. Including trials, diagnostic accuracy and acceptability studies

The following exclusion criteria were also applied:

i. Studies based in any clinical setting other than at the first assessment of suspicious skin lesions (i.e. primary care in the UK)
ii. Studies involving specialist clinicians (in line with the Cochrane review of dermoscopy which stated 'although primary care clinicians can in practice be specialists in skin cancer, we considered primary care physicians as generalist practitioners and dermatologists as specialists'[1].)
iii. Studies that are not primary studies

Search Strategy

An electronic literature search of Medline, EMBASE, CENTRAL, CINAHL, and SCOPUS was performed from January 1990 to December 2017 using a combination of subject headings as detailed in Appendix Two. The validity of the search strategy was assessed by cross-checking articles identified in the search against:

i. the reference lists of key review articles[1-5]
ii. papers identified by a first and last author search of six key papers in the field[6-11]

The search was then further extended by manually screening the reference lists of all included papers.

Study Selection

The study selection followed these stages:

1. The titles and abstracts of the complete list of identified papers were independently assessed by one researcher (OTJ) against the inclusion and exclusion criteria. Those studies clearly not relevant were rejected at this stage.
2. One or two other members of the research team (SH, LJ) independently assessed a random selection of 10% of the papers that have been excluded, to check agreement between the reviewers.
3. For studies where a definite decision to reject could not be made based on title and abstract alone, the full paper was obtained for detailed assessment.

For peer review only

4. At least two reviewers (OTJ, FW, SH, LJ, MvM) independently assessed the full-text of included articles for the possibility of inclusion in the review. Papers identified as not meeting the inclusion criteria by both researchers were excluded.
5. The researchers met regularly to discuss papers in which it is unclear whether they meet the inclusion criteria or not and any disagreements were resolved by discussion with other members of the research team.

Data Extraction

Included papers had relevant information extracted and entered into a spreadsheet, including:

1. The characteristics of the study including:
   a. Study details
   b. Location of study
   c. Study design
   d. Study population
      i. Primary care, referred, secondary care
      ii. Control comparator
      iii. Total number of benign/suspicious lesions and number of patients
      iv. Gender
      v. Age
   e. Study methods
      i. Selection of participants
      ii. Exclusions
      iii. Follow-up time
      iv. Definition of the outcome
2. The outcomes of the study in terms of:
   a. Number of lesions deemed banal and suspicious in control and intervention groups
   b. Number of excised lesions and excised benign lesions
   c. Outcome Measure: Sensitivity/specificity
   d. Outcome Measure: Positive Predictive Value/Negative Predictive Value
   e. Outcome Measure: Others (e.g. diagnostic rate of new test, accuracy index etc.)
   f. Outcome Measure: specific to melanoma
   g. Stage of melanoma at diagnosis
   h. 5-year survival rate
3. Challenges for implementation in primary care:
   a. Training
   b. Cost
   c. Availability
   d. Cost-effective analysis.

To minimise bias, data extraction was undertaken by the lead researcher (OTJ) and by at least one other researcher independently (FMW or LJ). The research team met regularly to ensure consensus of views by discussing and resolving any differences in data extraction.

Quality Assessment Framework

Quality of the studies was assessed using the Joanna Briggs Institute Critical Appraisal Tools (Joanna Briggs Institute, 2016). The aim of the quality assessment was to determine the validity of the results based on the design, methods,

For peer review only

analysis and conclusions of each study, and to assess the relative contribution of each study to the review. Limitations were acknowledged but studies were not excluded based on the quality assessment alone.

References

1. Dinnes J, Matin RN, Moreau JF, Patel L, Chan SA, Chuchu N, et al. Tests to assist in the diagnosis of cutaneous melanoma in adults: a generic protocol. Cochrane Database of Systematic Reviews. 2015.
2. Chappuis P, Duru G, Marchal O, Girier P, Dalle S, Thomas L. Dermoscopy, a useful tool for general practitioners in melanoma screening: A nationwide survey. British Journal of Dermatology. 2016;175:744-50.
3. Chia A, Trevena L. A Systematic Review of Training to Improve Melanoma Diagnostic Skills in General Practitioners. Journal of Cancer Education. 2016;31(4):730-5.
4. Koelink CJ, Jonkman MF, Van Der Meer K, Van Der Heide WK. Examination of skin lesions for cancer: which clinical decision aids and tools are available in general practice? European Journal of Dermatology. 2014;24(3):297-304.
5. Herschorn A. Dermoscopy for melanoma detection in family practice. Canadian Family Physician. 2012;58(7):740-5.
6. Harrington E, Clyne B, Wesseling N, Sandhu H, Armstrong L, Bennett H, et al. Diagnosing malignant melanoma in ambulatory care: A systematic review of clinical prediction rules. BMJ Open. 2017;7 (3) (no pagination)(e014096).
7. Koelink C, Vermeulen K, Kollen B, Bock G, Dekker J, Jonkman M, et al. Diagnostic accuracy and cost-effectiveness of dermoscopy in primary care: a cluster randomized clinical trial. Journal of the european academy of dermatology and venereology : JEADV [Internet]. 2014; 28(11):[1442-9 pp.].
8. Walter FM, Morris HC, Humphrys E, Hall PN, Prevost AT, Burrows N, et al. Effect of adding a diagnostic aid to best practice to manage suspicious pigmented lesions in primary care: randomised controlled trial. BMJ. 2012;345:e4110.
9. Plüddemann A, Heneghan C, Thompson M, Wolstenholme J, Price CP. Dermoscopy for the diagnosis of melanoma: Primary care diagnostic technology update. British Journal of General Practice. 2011;61(587):416-7.
10. Argenziano G, Puig S, Zalaudek I, Sera F, Corona R, Alsina M, et al. Dermoscopy improves accuracy of primary care physicians to triage lesions suggestive of skin cancer. Journal of Clinical Oncology. 2006;24(12):1877-82.
11. Gerbert B, Bronstone A, Maurer T, Berger T, McPhee SJ, Caspers N. The effectiveness of an Internet-based tutorial in improving primary care physicians' skin cancer triage skills. Journal of Cancer Education. 2002;17(1):7-11.

For peer review only

Appendix 2 – Search Strategies

The specific strategies for Medline, CENTRAL, EMBASE, CINAHL and SCOPUS were as follows:

A. Medline

1. primary health care.mp. or exp Primary Health Care/
2. general practice.mp. or exp General Practice/
3. family practice.mp. or exp Family Practice/
4. ((family or general or community) adj2 (doctor* or medicine or medical pratitioner* or medical practice* or practice* or practitioner* or healthcare* or health care*)).mp. [mp=title, abstract, original title, name of substance word, subject heading word, keyword heading word, protocol supplementary concept word, rare disease supplementary concept word, unique identifier, synonyms]
5. ((primary or community) adj2 (care* or health care* or healthcare* or medical care* or patient care*)).mp. [mp=title, abstract, original title, name of substance word, subject heading word, keyword heading word, protocol supplementary concept word, rare disease supplementary concept word, unique identifier, synonyms]
6. exp physicians, family/ or family physician*.mp.
7. (nurse* adj2 practi*).mp. [mp=title, abstract, original title, name of substance word, subject heading word, keyword heading word, protocol supplementary concept word, rare disease supplementary concept word, unique identifier, synonyms]
8. clinician with minimal dermoscopy training.mp.
9. melanoma screening clinic*.mp.
10. non-expert*.mp.
11. generalist clinician*.mp.
12. 1 or 2 or 3 or 4 or 5 or 6 or 7 or 8 or 9 or 10 or 11
13. dermoscopy.mp. or exp Dermoscopy/
14. dermatoscopy.mp.
15. epiluminescence microscopy.mp.
16. confocal microscopy.mp. or exp Microscopy, Confocal/
17. exp Photomicrography/ or photomicrograph.mp.
18. incident light microscopy.mp.
19. surface microscopy.mp.
20. dermoscopic.mp.
21. novel technique*.mp.

22. (siascope or siascopy or siascopic).mp. [mp=title, abstract, original title, name of substance word, subject heading word, keyword heading word, protocol supplementary concept word, rare disease supplementary concept word, unique identifier, synonyms]

23. molemate.mp.

24. AI.mp.

25. artificial intelligence.mp. or exp Artificial Intelligence/

26. exp Image Processing, Computer-Assisted/ or computer assisted.mp.

27. exp Diagnosis, Computer-Assisted/ or computer aided.mp.

28. exp "Neural Networks (Computer)"/ or neural network*.mp.

29. image analysis.mp.

30. automatic classification.mp.

31. machine learning.mp. or exp Machine Learning/

32. computer vision.mp.

33. digital analysis.mp.

34. digitally analysed.mp.

35. (analysis adj5 digitally).mp. [mp=title, abstract, original title, name of substance word, subject heading word, keyword heading word, protocol supplementary concept word, rare disease supplementary concept word, unique identifier, synonyms]

36. computer diagnos*.mp.

37. exp Mobile Applications/ or mobile app*.mp.

38. smartphone app*.mp. or exp Smartphone/

39. recognition skill*.mp.

40. automatic detection.mp.

41. collective intelligence assessment*.mp.

42. analysis device*.mp.

43. aid to diagnosis.mp.

44. diagnostic aid*.mp.

45. (decision adj3 tool*).mp. [mp=title, abstract, original title, name of substance word, subject heading word, keyword heading word, protocol supplementary concept word, rare disease supplementary concept word, unique identifier, synonyms]

For peer review only

46. tele-dermatos*.mp. [mp=title, abstract, original title, name of substance word, subject heading word, keyword heading word, protocol supplementary concept word, rare disease supplementary concept word, unique identifier, synonyms]

47. tele-dermos*.mp. [mp=title, abstract, original title, name of substance word, subject heading word, keyword heading word, protocol supplementary concept word, rare disease supplementary concept word, unique identifier, synonyms]

48. ((dermosc* or dermatosc* or epiluminescence microscopy or surface microscopy) adj3 (image* or picture* or photo*)).mp. [mp=title, abstract, original title, name of substance word, subject heading word, keyword heading word, protocol supplementary concept word, rare disease supplementary concept word, unique identifier, synonyms]

49. 13 or 14 or 15 or 16 or 17 or 18 or 19 or 20 or 21 or 22 or 23 or 24 or 25 or 26 or 27 or 28 or 29 or 30 or 31 or 32 or 33 or 34 or 35 or 36 or 37 or 38 or 39 or 40 or 41 or 42 or 43 or 44 or 45 or 46 or 47 or 48

50. exp Melanoma/ or melanoma*.mp.

51. exp Skin Neoplasms/ or skin neoplasm*.mp.

52. pigmented nevus.mp. or exp Nevus, Pigmented/

53. (naevocarcinoma or nevocarcinoma).mp. [mp=title, abstract, original title, name of substance word, subject heading word, keyword heading word, protocol supplementary concept word, rare disease supplementary concept word, unique identifier, synonyms]

54. ((neoplasm* or cancer* or tumour*) adj3 (skin or pigment*)).mp. [mp=title, abstract, original title, name of substance word, subject heading word, keyword heading word, protocol supplementary concept word, rare disease supplementary concept word, unique identifier, synonyms]

55. ((suspicious or melanocytic or pigmented) adj3 (lesion* or mole* or naevus or nevus or naevi or nevi or skin)).mp. [mp=title, abstract, original title, name of substance word, subject heading word, keyword heading word, protocol supplementary concept word, rare disease supplementary concept word, unique identifier, synonyms]

56. skin lesion*.mp.

57. 50 or 51 or 52 or 53 or 54 or 55 or 56

58. 12 and 49 and 57

For peer review only

B. CENTRAL

1. "primary health care":ti,ab,kw (Word variations have been searched)

2. "general practice":ti,ab,kw (Word variations have been searched)

3. "family practice":ti,ab,kw (Word variations have been searched)

4. family physician*:ti,ab,kw (Word variations have been searched)

5. MeSH descriptor: [Primary Health Care] explode all trees

6. MeSH descriptor: [General Practice] explode all trees

7. MeSH descriptor: [Family Practice] explode all trees

8. MeSH descriptor: [Physicians, Family] explode all trees

9. ((family or general or community) near/2 (doctor* or medicine or medical practitioner* or medical practice* or practice* or practitioner* or healthcare* or health care*)):ti,ab,kw (Word variations have been searched)

10. ((primary or community) near/2 (care* or health care* of healthcare* or medical care* or patient care*)):ti,ab,kw (Word variations have been searched)

11. (nurse* near/2 practi*):ti,ab,kw (Word variations have been searched)

12. clinician with minimal dermoscopy training:ti,ab,kw (Word variations have been searched)

13. melanoma screening clinic*:ti,ab,kw (Word variations have been searched)

14. non-expert*:ti,ab,kw (Word variations have been searched)

15. generalist clinician*:ti,ab,kw (Word variations have been searched)

16. #1 or #2 or #3 or #4 or #5 or #6 or #7 or #8 or #9 or #10 or #11 or #12 or #13 or #14 or #15

17. "dermoscopy":ti,ab,kw (Word variations have been searched)

18. dermatoscopy:ti,ab,kw (Word variations have been searched)

19. epiluminescence microscopy:ti,ab,kw (Word variations have been searched)

20. confocal microscopy:ti,ab,kw (Word variations have been searched)

21. photomicrograph:ti,ab,kw (Word variations have been searched)

22. incident light microscopy:ti,ab,kw (Word variations have been searched)

23. "surface microscopy":ti,ab,kw (Word variations have been searched)

24. "dermoscopic":ti,ab,kw (Word variations have been searched)

25. novel technique*:ti,ab,kw (Word variations have been searched)

26. siascop*:ti,ab,kw (Word variations have been searched)

27. molemate:ti,ab,kw (Word variations have been searched)

For peer review only

28. AI:ti,ab,kw (Word variations have been searched)

29. "artificial intelligence":ti,ab,kw (Word variations have been searched)

30. "computer assisted":ti,ab,kw (Word variations have been searched)

31. "computer aided":ti,ab,kw (Word variations have been searched)

32. "neural network":ti,ab,kw (Word variations have been searched)

33. image analysis:ti,ab,kw (Word variations have been searched)

34. automatic classification:ti,ab,kw (Word variations have been searched)

35. "machine learning":ti,ab,kw (Word variations have been searched)

36. computer vision:ti,ab,kw (Word variations have been searched)

37. digital analysis:ti,ab,kw (Word variations have been searched)

38. digitally analysed:ti,ab,kw (Word variations have been searched)

39. digitally analysed:ti,ab,kw (Word variations have been searched)

40. analysis near/5 digitally:ti,ab,kw (Word variations have been searched)

41. computer diagnos*:ti,ab,kw (Word variations have been searched)

42. mobile app*:ti,ab,kw (Word variations have been searched)

43. smartphone app*:ti,ab,kw (Word variations have been searched)

44. recognition skill*:ti,ab,kw (Word variations have been searched)

45. automatic detection:ti,ab,kw (Word variations have been searched)

46. collective intelligence assessment*:ti,ab,kw (Word variations have been searched)

47. analysis device*:ti,ab,kw (Word variations have been searched)

48. aid to diagnosis:ti,ab,kw (Word variations have been searched)

49. diagnostic aid*:ti,ab,kw (Word variations have been searched)

50. decision near/3 tool*:ti,ab,kw (Word variations have been searched)

51. tele-dermatos*:ti,ab,kw (Word variations have been searched)

52. tele-dermos*:ti,ab,kw (Word variations have been searched)

53. ((dermoscopy or dermatoscopy or epiluminescence microscopy or surface microscopy or dermoscopic) near/3 (image* or picture* or photo*)):ti,ab,kw (Word variations have been searched)

54. MeSH descriptor: [Dermoscopy] explode all trees

55. MeSH descriptor: [Microscopy, Confocal] explode all trees

For peer review only

56. MeSH descriptor: [Photomicrography] explode all trees

57. MeSH descriptor: [Artificial Intelligence] explode all trees

58. MeSH descriptor: [Diagnosis, Computer-Assisted] explode all trees

59. MeSH descriptor: [Image Processing, Computer-Assisted] explode all trees

60. MeSH descriptor: [Neural Networks (Computer)] explode all trees

61. MeSH descriptor: [Machine Learning] explode all trees

62. MeSH descriptor: [Mobile Applications] explode all trees

63. MeSH descriptor: [Smartphone] explode all trees

64. #17 or #18 or #19 or #20 or #21 or #22 or #23 or #24 or #25 or #26 or #27 or #28 or #29 or #30 or #31 or #32 or #33 or #34 or #35 or #36 or #37 or #38 or #39 or #40 or #41 or #42 or #43 or #44 or #45 or #46 or #47 or #48 or #49 or #50 or #51 or #52 or #53 or #54 or #55 or #56 or #57 or #58 or #59 or #60 or #61 or #62 or #63

65. "skin neoplasm*":ti,ab,kw (Word variations have been searched)

66. melanoma*:ti,ab,kw (Word variations have been searched)

67. "pigmented nevus":ti,ab,kw (Word variations have been searched)

68. skin lesion*:ti,ab,kw (Word variations have been searched)

69. naevocarcinoma or nevocarcinoma:ti,ab,kw (Word variations have been searched)

70. ((neoplasm* or cancer* or tumour*) near/3 (skin or pigment*)):ti,ab,kw (Word variations have been searched)

71. ((suspicious or melanocytic or pigmented) near/3 (lesion* or mole* or naevus or nevus or naevi or nevi or skin)):ti,ab,kw (Word variations have been searched)

72. MeSH descriptor: [Melanoma] explode all trees

73. MeSH descriptor: [Skin Neoplasms] explode all trees

74. MeSH descriptor: [Nevus, Pigmented] explode all trees

75. #65 or #66 or #67 or #68 or #69 or #70 or #71 or #72 or #73 or #74

76. #16 and #64 and #75

For peer review only

C. EMBASE

1. exp *primary health care/
2. primary health care.ti,ab.
3. exp *general practice/
4. general practice.ti,ab.
5. family practice.ti,ab.
6. ((family or general or community) adj2 (doctor* or medicine or medical practitioner* or medical practice* or practice* or practitioner* or healthcare* or health care*)).ti,ab.
7. ((primary or community) adj2 (care* or health care* or healthcare* or medical care* or patient care*)).ti,ab.
8. family physician*.ti,ab.
9. (nurse* adj2 practi*).ti,ab.
10. clinician with minimal dermoscopy training.ti,ab.
11. melanoma screening clinic*.ti,ab.
12. non-expert*.ti,ab.
13. generalist clinician*.ti,ab.
14. 1 or 2 or 3 or 4 or 5 or 6 or 7 or 8 or 9 or 10 or 11 or 12 or 13
15. exp *epiluminescence microscopy/
16. epiluminescence microscopy.ti,ab.
17. dermoscopy.ti,ab.
18. dermatoscopy.ti,ab.
19. exp *confocal microscopy/
20. confocal microscopy.ti,ab.
21. photomicrograph*.ti,ab.
22. incident light microscopy.ti,ab.
23. surface microscopy.ti,ab.
24. dermoscopic.ti,ab.
25. novel technique*.ti,ab.
26. (siascope or siascopy or siascopic).ti,ab.
27. molemate.ti,ab.
28. AI.ti,ab.
29. exp *artificial intelligence/
30. artificial intelligence.ti,ab.
31. exp computer assisted diagnosis/
32. computer assisted.ti,ab.
33. exp computer analysis/
34. computer aided.ti,ab.
35. computer analysis.ti,ab.
36. exp *artificial neural network/
37. neural network*.ti,ab.
38. exp *image analysis/
39. image analysis.ti,ab.
40. automatic classification.ti,ab.
41. exp *machine learning/
42. machine learning.ti,ab.
43. computer vision.ti,ab.
44. digital analysis.ti,ab.

For peer review only

45. digitally analy*.ti,ab.
46. (analysis adj5 digitally).ti,ab.
47. computer diagnos*.ti,ab.
48. exp *mobile application/
49. mobile app*.ti,ab.
50. exp *smartphone/
51. smartphone app*.ti,ab.
52. recognition skill*.ti,ab.
53. automatic detection.ti,ab.
54. collective intelligence assessment*.ti,ab.
55. analysis device*.ti,ab.
56. aid to diagnosis.ti,ab.
57. diagnostic aid*.ti,ab.
58. (decision adj3 tool*).ti,ab.
59. tele-dermatos*.ti,ab.
60. tele-dermos*.ti,ab.
61. ((dermoscopy or dermatoscopy or epiluminescence microscopy or surface microscopy or dermoscopic) adj3 (image* or picture* or photo*)).ti,ab.
62. 15 or 16 or 17 or 18 or 19 or 20 or 21 or 22 or 23 or 24 or 25 or 26 or 27 or 28 or 29 or 30 or 31 or 32 or 33 or 34 or 35 or 36 or 37 or 38 or 39 or 40 or 41 or 42 or 43 or 44 or 45 or 46 or 47 or 48 or 49 or 50 or 51 or 52 or 53 or 54 or 55 or 56 or 57 or 58 or 59 or 60 or 61
63. exp *melanoma/ or exp *melanoma skin cancer/
64. melanoma*.ti,ab.
65. exp *skin tumor/ or exp *skin cancer/
66. skin neoplasm*.ti,ab.
67. exp *pigmented nevus/
68. pigmented naevus.ti,ab.
69. skin lesion*.ti,ab.
70. (naevocarcinoma or nevocarcinoma).ti,ab.
71. ((neoplasm* or cancer* or tumour*) adj3 (skin or pigment*)).ti,ab.
72. ((suspicious or melanocytic or pigmented) adj3 (lesion* or mole* or naevus or nevus or naevi or nevi or skin)).ti,ab.
73. 63 or 64 or 65 or 66 or 67 or 68 or 69 or 70 or 71 or 72
74. 14 and 62 and 73

For peer review only

D. CINAHL

66 S15 AND S56 AND S65
65 S57 OR S58 OR S59 OR S60 OR S61 OR S62 OR S63 OR S64
64 (MH "Skin Neoplasms+")
63 (MH "Melanoma+")
62 ((suspicious or melanocytic or pigment*) N3 (lesion* or mole* or naevus or nevus or naevi or nevi or skin))
61 ((tumour* or neoplasm* or cancer*) N3 (skin or pigment*))
60 naevocarcinoma or nevocarcinoma
59 skin lesion*
58 skin neoplasm*
57 melanoma*
56 S16 OR S17 OR S18 OR S19 OR S20 OR S21 OR S22 OR S23 OR S24 OR S25 OR S26 OR S27 OR S28 OR S29 OR S30 OR S31 OR S32 OR S33 OR S34 OR S35 OR S36 OR S37 OR S38 OR S39 OR S40 OR S41 OR S42 OR S43 OR S44 OR S45 OR S46 OR S47 OR S48 OR S49 OR S50 OR S51 OR S52 OR S53 OR S54 OR S55
55 (MH "Smartphone+")
54 (MH "Mobile Applications")
53 (MH "Neural Networks (Computer)")
52 (MH "Image Processing, Computer Assisted+") OR (MH "Image Interpretation, Computer Assisted+") OR (MH "Diagnosis, Computer Assisted+")
51 (MH "Artificial Intelligence+")
50 ((dermoscop* or dermatoscopy or epiluminescence microscopy or surface microscopy) N3 (image* or picture* or photo*))
49 tele-dermatos*
48 tele-dermos*
47 decision N3 tool*
46 diagnos* N4 aid*
45 analysis device*
44 collective intelligence assessment*
43 automatic detection
42 recognition skill*
41 smartphone app*
40 mobile app*
39 computer diagnos*
38 analysis N5 digital*
37 digitally analysed
36 digital analysis
35 computer vision
34 machine learning
33 automatic classification
32 image analysis
31 neural network*
30 computer aided
29 computer assisted
28 artificial intelligence
27 AI
26 molemate

For peer review only

25 siascop*
24 novel technique*
23 dermoscopic
22 surface microscopy
21 incident light microscopy
20 photomicrograph
19 confocal microscopy
18 epiluminescence microscopy
17 dermatoscopy
16 dermoscopy
15 S1 OR S2 OR S3 OR S4 OR S5 OR S6 OR S7 OR S8 OR S9 OR S10 OR S11 OR S12 OR S13 OR S14
14 (MH "Physicians, Family")
13 (MH "Family Practice")
12 (MH "Primary Health Care")
11 generalist clinician*
10 non-expert*
9 melanoma screening clinic*
8 clinician with minimal dermoscopy training
7 nurse* N2 practi*
6 family physician*
5 ((primary or community) N2 (care* or health care* or healthcare* or medical care* or patient care*))
4 ((family or general or community) N2 (doctor* or medicine or medical practitioner* or medical practice* or practice* or practitioner* or healthcare* or health care*))
3 family practice
2 general practice
1 primary health care

For peer review only

E. SCOPUS

(((TITLE-ABS-KEY(primary w/1 (health w/1 care))) OR (TITLE-ABS-KEY(general w/1 practice)) OR (TITLE-ABS-KEY(family w/1 practice)) OR (TITLE-ABS-KEY(family w/1 physician*)) OR (TITLE-ABS-KEY((family W/2 doctor*) OR (family W/2 medicine) OR (family W/2 (medical w/1 practitioner*)) OR (family W/2 (medical w/1 practice*)) OR (family W/2 practice*) OR (family W/2 practitioner*) OR (family W/2 healthcare*) OR (family W/2 (health w/1 care*)))) OR (TITLE-ABS-KEY((general W/2 doctor*) OR (general W/2 medicine) OR (general W/2 (medical w/1 practitioner*)) OR (general W/2 (medical w/1 practice*)) OR (general W/2 practice*) OR (general W/2 practitioner*) OR (general W/2 healthcare*) OR (general W/2 (health w/1 care*)))) OR (TITLE-ABS-KEY((community W/2 doctor*) OR (community W/2 medicine) OR (community W/2 (medical w/1 practitioner*)) OR (community W/2 (medical w/1 practice*)) OR (community W/2 practice*) OR (community W/2 practitioner*) OR (community W/2 healthcare*) OR (community W/2 (health w/1 care*))))) OR ((TITLE-ABS-KEY((community W/2 care*) OR (community W/2 (medical w/1 care*)) OR (community W/2 (patient w/1 care*)) OR (community W/2 healthcare*) OR (community W/2 (health w/1 care*)))) OR (TITLE-ABS-KEY((primary W/2 care*) OR (primary W/2 (medical w/1 care*)) OR (primary W/2 (patient w/1 care*)) OR (primary W/2 healthcare*) OR (primary W/2 (health w/1 care*)))) OR (TITLE-ABS-KEY(nurs* W/2 practi*)) OR (TITLE-ABS-KEY(clinician w/1 with w/1 minimal w/1 dermoscopy w/1 training)) OR (TITLE-ABS-KEY(melanoma w/1 screening w/1 clinic*)) OR (TITLE-ABS-KEY(generalist w/1 clinician*))))

AND

(((TITLE-ABS-KEY(surface w/1 microscopy)) OR (TITLE-ABS-KEY(dermoscopic)) OR (TITLE-ABS-KEY(novel w/1 technique)) OR (TITLE-ABS-KEY(siascop*)) OR (TITLE-ABS-KEY(molemate)) OR (TITLE-ABS-KEY(ai)) OR (TITLE-ABS-KEY(artificial w/1 intelligence))) OR ((TITLE-ABS-KEY(computer w/1 assisted)) OR (TITLE-ABS-KEY(computer w/1 aided)) OR (TITLE-ABS-KEY(neural w/1 network)) OR (TITLE-ABS-KEY(image w/1 analysis)) OR (TITLE-ABS-KEY(automatic w/1 classification)) OR (TITLE-ABS-KEY(machine w/1 learning)) OR (TITLE-ABS-KEY(computer w/1 vision))) OR ((TITLE-ABS-KEY(digital* W/5 analys*)) OR (TITLE-ABS-KEY(computer w/1 diagnos*)) OR (TITLE-ABS-KEY(mobile w/1 app*)) OR (TITLE-ABS-KEY(smartphone)) OR (TITLE-ABS-KEY(recognition w/1 skill*)) OR (TITLE-ABS-KEY(automatic w/1 detection)) OR (TITLE-ABS-KEY(collective w/1 intelligence AND assessment*))) OR ((TITLE-ABS-KEY(analysis w/1 device*)) OR (TITLE-ABS-KEY(diagnos* W/3 aid*)) OR (TITLE-ABS-KEY(decision W/3 aid*)) OR (TITLE-ABS-KEY(decision W/3 tool*)) OR (TITLE-ABS-KEY(tele-dermos*)) OR (TITLE-ABS-KEY(tele-dermatos*)) OR (TITLE-ABS-KEY((dermos* W/3 image*) OR (dermos* W/3 photo*) OR (dermos* W/3 picture*)))) OR ((TITLE-ABS-KEY((dermatos* W/3 image*) OR (dermatos* W/3 photo*) OR (dermatos* W/3 picture*))) OR (TITLE-ABS-KEY((epiluminescence W/4 image*) OR (epiluminescence W/4 photo*) OR (epiluminescence W/4 picture*))) OR (TITLE-ABS-KEY(((surface w/1 microscopy) W/4 image*) OR ((surface w/1 microscopy) W/4 photo*) OR ((surface w/1 microscopy) W/4 picture*)))) OR ((TITLE-ABS-KEY(dermoscopy)) OR (TITLE-ABS-KEY(dermatoscopy)) OR (TITLE-ABS-KEY(epiluminescence w/1 microscopy)) OR (TITLE-ABS-KEY(confocal w/1 microscopy)) OR (TITLE-ABS-KEY(photomicrograph)) OR (TITLE-ABS-KEY(incident w/1 light w/1 microscopy))))

AND

((TITLE-ABS-KEY(melanoma)) OR (TITLE-ABS-KEY(naevocarcinoma OR nevocarcinoma)) OR (TITLE-ABS-KEY((pigment* W/3 neoplasm*) OR (pigment* W/3 cancer*) OR (pigment* W/3 tumo*) OR (pigment* W/3 lesion*))) OR (TITLE-ABS-KEY((pigment* W/3 lesion*) OR (pigment* W/3 mole*) OR (pigment* W/3 naev*) OR (pigment* W/3 nev*) OR (pigment* W/3 skin))) OR (TITLE-ABS-KEY((melanocytic W/3 lesion*) OR (melanocytic W/3 mole*) OR (melanocytic W/3 naev*) OR (melanocytic W/3 nev*) OR (melanocytic W/3 skin))) OR (TITLE-ABS-KEY((suspicious W/3 lesion*) OR (suspicious W/3 mole*) OR (suspicious W/3 naev*) OR (suspicious W/3 nev*) OR (suspicious W/3 skin))) OR (TITLE-ABS-KEY((skin W/3 neoplasm*) OR (skin W/3 cancer*) OR (skin W/3

For peer review only - http://bmjopen.bmj.com/site/about/guidelines.xhtml

tumo*) OR (skin W/3 lesion*))))

For peer review only

Appendix 3 – Full list of studies that underwent full text review, their assessors and outcomes

Paper	Assessor					Reason for exclusion				
	OJ	LJ	SH	FW	MvM	No dermoscopy use	Not in 1ary care	Not peer reviewed / conference abstract	No primary data	Clinical Audit
Affifi *et al*. 2017 (A)	Exclude		Exclude			■				
Affifi *et al*. 2017 (B)	Exclude		Exclude			■				
Ahmadi *et al*. 2017	Include	Include								
Alonso *et al*. 2016	Exclude		Exclude					■		
Argenziano *et al*. 2006	Include		Include							
Baldi *et al*. 2010	Exclude	Exclude							■	
Bataille *et al*. 2011	Exclude	Exclude						■		
Bergman *et al*. 2008	Exclude				Exclude				■	
Bergstrom *et al*. 2012	Exclude								■	
Berryman *et al*. 2012	Exclude		Exclude			■				
Blackledge *et al*. 2009	Exclude	Exclude				■				
Borve *et al*. 2015	Include	Include								
Bourne *et al*. 2012	Include			Include						
Bowns *et al*. 2006	Exclude			Exclude			■			
Brinkhuijsen *et al*. 2008	Exclud				Exclud				■	

For peer review only

	e				e					
Brown *et al*. 2000	Exclude		Exclude							
Carli *et al*. 2007	Exclude		Exclude							
Carrara *et al*. 2007	Exclude	Exclude								
Chao Li *et al*. 2013	Exclude			Exclude						
Chappuis *et al*. 2016	Include		Include							
Chen *et al*. 2011	Exclude	Exclude		Exclude						
Congalton *et al*. 2015	Exclude			Exclude						
Cook *et al*. 2015	Exclude	Exclude								
Coras *et al*. 2003	Exclude	Exclude								
Dahlen Gyllencreutz *et al*. 2017	Exclude			Exclude						
Del Mar *et al*. 1995	Exclude		Exclude							
Dolianitis *et al*. 2005	Exclude			Exclude						
Drugge *et al*. 2010	Exclude	Exclude								
Ferrandiz *et al*. 2017	Include			Include						
Greenberg *et al*. 2012	Exclude	Exclude								
Griffiths *et al*. 2010	Exclude			Exclude						
Grimaldi *et al*. 2009	Include			Include						
Hennessy *et al*. 2010	Exclude		Exclude							

For peer review only

Jain *et al*. 2012	Exclude	Exclude	Exclude							
Joliffe *et al*. 2001 (A)	Exclude			Exclude						
Joliffe *et al*. 2001 (B)	Exclude			Exclude						
Kenney *et al*. 2016	Exclude			Exclude						
Kitchener *et al*. 2009	Exclude	Exclude	Exclude							
Koelink *et al*. 2014	Include	Include								
Kroemer *et al*. 2001	Exclude			Exclude						
Laheru *et al*. 2017	Exclude			Exclude						
Livingstone *et al*. 2015	Include			Include						
Lorentzen *et al*. 1999	Exclude	Exclude		Exclude						
MacKinnon *et al*. 2016	Exclude		Exclude							
Mahendran *et al*. 2005	Exclude	Exclude								
Masood *et al*. 2014 (A)	Exclude	Exclude								
Masood *et al*. 2014 (B)	Exclude		Exclude							
Masood *et al*. 2015	Exclude		Exclude							
Massone *et al*. 2014	Exclude			Exclude						
Menzies *et al*. 2005	Include	Include								
Menzies *et al*. 2009	Include		Include							
Mikolajcyk *et al*. 2017	Exclud	Exclud								

For peer review only

	e	e								
Monheit *et al*. 2011	Exclude		Exclude							
Moreno-Ramirez *et al*. 2006	Include			Include						
Moreno-Ramirez *et al*. 2007	Exclude	Exclude								
Morris *et al*. 2017 (A)	Include		Include							
Morris *et al*. 2017 (B)	Include	Include								
Morton *et al*. 2011	Exclude			Exclude						
Naka *et al*. 2017	Exclude	Exclude		Exclude						
Nelson *et al*. 2016	Exclude			Exclude						
Pagnanelli *et al*. 2003	Include		Include							
Perakis *et al*. 2014	Exclude	Exclude								
Phelan *et al*. 2008	Exclude			Exclude						
Picollo *et al*. 2000	Exclude			Exclude						
Picollo *et al*. 2002	Exclude			Exclude						
Rademaker *et al*. 2010	Exclude			Exclude						
Riding *et al*. 2016	Exclude		Exclude							
Robson *et al*. 2012*	Exclude	Exclude								
Rogers *et al*. 2016	Include			Include						
Rogers *et al*. 2017	Include			Include						

For peer review only

Rosendahl *et al*. 2011	Include	Include								
Rosendahl *et al*. 2012 (A)	Exclude	Exclude	Exclude							
Rosendahl *et al*. 2012 (B)	Include			Include						
Secker *et al*. 2014	Include	Include								
Shapiro *et al*. 2004	Exclude		Exclude							
Sheraz *et al*. 2011	Exclude	Exclude								
Singh *et al*. 2016	Exclude	Exclude	Exclude							
Skrovseth *et al*. 2010	Exclude	Exclude								
Smith *et al*. 2000	Exclude	Exclude								
Soyer *et al*. 2004	Exclude		Exclude	Exclude						
Stratton *et al*. 2016	Include	Include		Include						
Swanson *et al*. 2017	Exclude	Exclude								
Tan *et al*. 2010	Exclude			Exclude						
Tan *et al*. 2015	Exclude		Exclude							
Thomas *et al*. 2017	Exclude	Exclude								
Van der Heijden *et al*. 2013	Include			Include						
Wadhawan *et al*. 2011	Exclude			Exclude						
Watts *et al*. 2017	Exclude		Exclude							
Westerhoff *et al*. 2000	Includ	Includ								

For peer review only

	e	e								
Winkelmann *et al*. 2015 (A)	Exclude		Exclude							
Winkelmann *et al*. 2015 (B)	Exclude		Exclude							
Wray *et al*. 2015	Exclude			Exclude						
Zalaudek *et al*. 2008	Exclude			Exclude						
Zortea *et al*. 2014	Exclude		Exclude							
Zouridakis *et al*. 2015	Exclude	Exclude	Exclude							

For peer review only

Reporting checklist for systematic review and meta-analysis.

Based on the PRISMA guidelines.

Instructions to authors

Complete this checklist by entering the page numbers from your manuscript where readers will find each of the items listed below.

Your article may not currently address all the items on the checklist. Please modify your text to include the missing information. If you are certain that an item does not apply, please write "n/a" and provide a short explanation.

Upload your completed checklist as an extra file when you submit to a journal.

In your methods section, say that you used the PRISMA reporting guidelines, and cite them as:

Moher D, Liberati A, Tetzlaff J, Altman DG, The PRISMA Group. Preferred Reporting Items for Systematic Reviews and Meta-Analyses: The PRISMA Statement

		Reporting Item	Page Number
	#1	Identify the report as a systematic review, meta-analysis, or both.	1
Structured summary	#2	Provide a structured summary including, as applicable: background; objectives; data sources; study eligibility criteria, participants, and interventions; study appraisal and synthesis methods; results; limitations; conclusions and implications of key findings; systematic review registration number	2
Rationale	#3	Describe the rationale for the review in the context of what is already known.	3
Objectives	#4	Provide an explicit statement of questions being addressed with reference to participants, interventions, comparisons, outcomes, and study design (PICOS).	3
Protocol and registration	#5	Indicate if a review protocol exists, if and where it can be accessed (e.g., Web address) and, if available, provide registration information including the registration number.	4

For peer review only

Eligibility criteria	#6	Specify study characteristics (e.g., PICOS, length of follow-up) and report characteristics (e.g., years considered, language, publication status) used as criteria for eligibility, giving rational	4
Information sources	#7	Describe all information sources in the search (e.g., databases with dates of coverage, contact with study authors to identify additional studies) and date last searched.	4
Search	#8	Present full electronic search strategy for at least one database, including any limits used, such that it could be repeated.	Appendix 2
Study selection	#9	State the process for selecting studies (i.e., for screening, for determining eligibility, for inclusion in the systematic review, and, if applicable, for inclusion in the meta-analysis).	See note 1
Data collection process	#10	Describe the method of data extraction from reports (e.g., piloted forms, independently by two reviewers) and any processes for obtaining and confirming data from investigators.	See note 2
Data items	#11	List and define all variables for which data were sought (e.g., PICOS, funding sources), and any assumptions and simplifications made.	Appendix 1
Risk of bias in individual studies	#12	Describe methods used for assessing risk of bias in individual studies (including specification of whether this was done at the study or outcome level, or both), and how this information is to be used in any data synthesis.	See note 3
Summary measures	#13	State the principal summary measures (e.g., risk ratio, difference in means).	4
Planned methods of analyis	#14	Describe the methods of handling data and combining results of studies, if done, including measures of consistency (e.g., I2) for each meta-analysis.	4
Risk of bias across studies	#15	Specify any assessment of risk of bias that may affect the cumulative evidence (e.g., publication bias, selective reporting within studies).	4
Additional analyses	#16	Describe methods of additional analyses (e.g., sensitivity or subgroup analyses, meta-regression), if done, indicating which were pre-specified.	4
Study selection	#17	Give numbers of studies screened, assessed for eligibility, and included in the review, with reasons for exclusions at each stage, ideally with a flow diagram.	See note 4

For peer review only

Study characteristics	#18	For each study, present characteristics for which data were extracted (e.g., study size, PICOS, follow-up period) and provide the citation.	See note 5
Risk of bias within studies	#19	Present data on risk of bias of each study and, if available, any outcome-level assessment (see Item 12).	See note 6
Results of individual studies	#20	For all outcomes considered (benefits and harms), present, for each study: (a) simple summary data for each intervention group and (b) effect estimates and confidence intervals, ideally with a forest plot.	See note 7
Synthesis of results	#21	Present the main results of the review. If meta-analyses are done, include for each, confidence intervals and measures of consistency.	pages 5-22
Risk of bias across studies	#22	Present results of any assessment of risk of bias across studies (see Item 15).	N/a
Additional analysis	#23	Give results of additional analyses, if done (e.g., sensitivity or subgroup analyses, meta-regression [see Item 16]).	N/a
Summary of Evidence	#24	Summarize the main findings, including the strength of evidence for each main outcome; consider their relevance to key groups (e.g., health care providers, users, and policy makers	22
Limitations	#25	Discuss limitations at study and outcome level (e.g., risk of bias), and at review level (e.g., incomplete retrieval of identified research, reporting bias).	23
Conclusions	#26	Provide a general interpretation of the results in the context of other evidence, and implications for future research.	23
Funding	#27	Describe sources of funding or other support (e.g., supply of data) for the systematic review; role of funders for the systematic review.	24

Author notes

1. 4 and appendix 1
2. 4 and appendix 1
3. 4 & 5 & appendix 1
4. 5 and PRISMA diagram (fig.1)
5. Pages 5, 8 and 10 (tables 1, 2 & 3)
6. Page 8 & 9 (table 2)

For peer review only

7. Pages 10-19

The PRISMA checklist is distributed under the terms of the Creative Commons Attribution License CC-BY.
This checklist was completed on 26. October 2018 using http://www.goodreports.org/, a tool made by the EQUATOR Network in collaboration with Penelope.ai

For peer review only

BMJ Open

Dermoscopy for melanoma detection and triage in primary care: A systematic review

Journal:	*BMJ Open*
Manuscript ID	bmjopen-2018-027529.R1
Article Type:	Research
Date Submitted by the Author:	05-Feb-2019
Complete List of Authors:	Jones, Owain; University of Cambridge Department of Public Health and Primary Care,<br>Jurascheck, Leo; University of Cambridge School of Clinical Medicine<br>van Melle, Marije; University of Cambridge Department of Public Health and Primary Care<br>Hickman, Sarah; Norfolk and Norwich University Hospitals NHS Foundation Trust<br>Burrows, Nigel; Cambridge University Hospitals NHS Foundation Trust, Addenbrooke's Hospital Department of Dermatology<br>Hall, Per; Cambridge University Hospitals NHS Foundation Trust, Addenbrooke's Hospital<br>Emery, Jon; University of Melbourne, General Practice and Primary Care Academic Centre; University of Cambridge Department of Public Health and Primary Care<br>Walter, Fiona; University of Cambridge, Dept of Public Health and Primary Care; University of Melbourne, General Practice and Primary Care Academic Centre
**Primary Subject Heading**:	General practice / Family practice
Secondary Subject Heading:	Dermatology, Diagnostics
Keywords:	Dermoscopy, Melanoma, PRIMARY CARE, Systematic review, Skin Cancer

SCHOLARONE™ Manuscripts

Dermoscopy for melanoma detection and triage in primary care: A systematic review

Running head: Dermoscopy use in primary care: a systematic review

Manuscript word, table and figure count: 2937 words, 4 tables, 1 figure

OT Jones[1], LC Jurascheck[2], MA van Melle[1], S Hickman[3], NP Burrows[4], PN Hall[4], J Emery[5,1], FM Walter[1,5]

[1]*The Primary Care Unit, Department of Public Health and Primary Care, University of Cambridge, UK*
[2]*Clinical School, Addenbrooke's Hospital, University of Cambridge, UK*
[3]*Norwich and Norfolk University Hospitals NHS Foundation Trust, Norwich, NR4 7UY*
[4]*Cambridge University Hospitals NHS Foundation Trust, Addenbrooke's Hospital, Cambridge, UK*
[5]*Department of General Practice and the Centre for Cancer Research, Faculty of Medicine, Dentistry and Health Science, Victorian Comprehensive Cancer Centre, University of Melbourne, Australia*

Corresponding author
Dr OT Jones, Department of Public Health and Primary Care, University of Cambridge, UK
E-mail: otj24@medschl.cam.ac.uk

For peer review only

For peer review only - http://bmjopen.bmj.com/site/about/guidelines.xhtml

Summary

Objective: Most skin lesions first present in primary care where distinguishing rare melanomas from benign lesions can be challenging. Dermoscopy improves diagnostic accuracy among specialists and is promoted for use by primary care physicians (PCPs). However, when used by untrained clinicians, accuracy may be no better than visual inspection. This study aimed to undertake a systematic review of literature reporting use of dermoscopy to triage suspicious skin lesions in primary care settings, and challenges for implementation.

Design: A systematic literature review and narrative synthesis.

Data Sources: We searched Medline, Cochrane, EMBASE, CINAHL and SCOPUS bibliographic databases from 1st January 1990 to 31st December 2017, without language restrictions.

Inclusion criteria: Studies including assessment of dermoscopy accuracy, acceptability to patients and PCPs, training requirements, and cost-effectiveness of dermoscopy modes in primary care, including trials, diagnostic accuracy and acceptability studies.

Results: 23 studies met the review criteria, representing 49,769 lesions and 3,708 PCPs, all from high-income countries. There was a paucity of studies set truly in primary care and the outcomes measured were diverse. The heterogeneity therefore made meta-analysis unfeasible; the data were synthesised through narrative review. Dermoscopy, with appropriate training, was associated with improved diagnostic accuracy for melanoma and benign lesions, and reduced unnecessary excisions and referrals. Teledermoscopy-based referral systems improved triage accuracy. Only 3 studies examined cost-effectiveness, hence there was insufficient evidence to draw conclusions. Costs, training and time requirements were considered important implementation barriers. Patient satisfaction was seldom assessed. Computer-aided dermoscopy and other technological advances have not yet been tested in primary care.

Conclusions: Dermoscopy could help PCPs triage suspicious lesions for biopsy, urgent referral or reassurance. However, it will be important to establish further evidence on minimum for training to reach competence, as well as the cost-effectiveness and patient acceptability of implementing dermoscopy in primary care.

Strengths and limitations of this study

- This study systematically reviews the published evidence for dermoscopy use by PCPs in primary care settings, including studies of acceptability and cost-effectiveness as well as diagnostic accuracy studies.
- The use of a broad search strategy across multiple databases enabled us to identify 23 studies whose findings examine dermoscopy use in primary care clinical practice.
- The included studies were of varying quality.
- Due to the heterogeneity of the included papers we were not able to undertake any meta-analysis; instead, we performed a narrative synthesis.

For peer review only - http://bmjopen.bmj.com/site/about/guidelines.xhtml

Introduction

Worldwide malignant melanoma is the 15th commonest cancer.[1] Melanoma has one of the fastest rising incidence rates of any cancer, and among white populations incidence has quadrupled over the last 30 years. In the UK this is projected to rise by a further 7% between 2014 and 2035, reflecting increasing exposure to the main risk factor, UV radiation.[2] There were nearly 300,000 new cases of melanoma worldwide in 2018.[1]

Primary care (the first point of contact for patients in the healthcare system, usually community-based) can play an important role in improving outcomes for patients with melanoma. More accurate triage of suspicious pigmented skin lesions could lead to more prompt diagnosis of melanoma at earlier stages and improved outcomes, and reduce unnecessary biopsies or referrals. Most people diagnosed with cancer first present in primary care,[3] where primary care physicians (PCPs) need to distinguish rare melanomas from common benign lesions using clinical history taking and visual inspection, aided by checklists such as the seven-point checklist as recommended in the 2015 NICE guidelines for suspected cancer.[4] Various technologies may also have a role in assisting triage of suspicious skin lesions, including mobile phone applications,[5] reflectance confocal microscopy,[6] optical coherence tomography,[7] computer-aided diagnosis,[8] high frequency ultrasound,[9] and dermoscopy.[10]

Dermoscopy (also referred to as dermatoscopy or epiluminescence microscopy) is a non-invasive technique using a hand-held magnifier and incident light, which may be polarised to reduce reflection, to reveal subsurface structures. Dermoscopy performed by trained specialists is more sensitive and specific in classifying skin lesions than clinical examination with the naked eye alone.[4, 11] Dermatologists and some international guidelines recommend PCPs use dermoscopy;[12] however, when used by untrained or less experienced clinicians, accuracy can be no better than inspection alone,[13] and there is a danger of increased excisions, over-referral or false reassurance. It takes time to train clinicians to use dermoscopy, and PCP training dropout rates have been shown to be high.[14, 15] For these reasons dermoscopy is not currently recommended for use by PCPs in the UK,[4] although it is used routinely by PCPs in Australia,[16] which has the highest incidence of melanoma worldwide. Some digital dermoscopy devices exist, a few of which incorporate computer-aided diagnosis, but they are expensive, and, while showing better sensitivity, even in expert hands many have lower specificity than clinicians alone.[17] However, recent research suggests computer-aided diagnostic tools have the potential to exceed the diagnostic performance of dermatologists.[18]

A Cochrane review of dermoscopy has recently been published, and examines the diagnostic accuracy of dermoscopy, with and without visual inspection, for the detection of cutaneous invasive melanoma and intra-epidermal melanocytic variants in adults.[19] Our systematic review has a broader aim, focussing on the first presentation of suspicious skin lesions in primary care and whether dermoscopy and dermoscopy-related technologies, with suitable training, can be used accurately and effectively to triage suspicious skin lesions at this point in the healthcare pathway. We considered various types of dermoscopy technologies, including hand-held dermoscopy, computer-aided/digital dermoscopy devices, and novel tele-dermoscopy approaches (i.e. referral using electronic dermoscopy images or video). In addition to data on the diagnostic accuracy of dermoscopy, we looked for data on the practical challenges to implementing dermoscopy in primary

For peer review only - http://bmjopen.bmj.com/site/about/guidelines.xhtml

care, including utility, acceptability to patients and PCPs, training requirements, and cost-effectiveness.

Methods

This systematic review was conducted in accordance with the PRISMA guidelines,[20] and the protocol was registered with PROSPERO (CRD42018091395) prior to conducting the review.[21] All aspects of the protocol were reviewed by senior faculty from the CanTest collaborative (www.cantest.org).

We searched the MEDLINE, EMBASE, Cochrane Central, CINAHL and SCOPUS databases using keywords related to dermoscopy, melanoma and primary care, without language restrictions, from 1st January 1990 to 31st December 2017. We also manually searched reference lists of included studies. We included all types of study design as we anticipated that there would be few relevant randomised controlled trials (RCTs) or diagnostic accuracy studies performed in primary care, and we aimed to find additional qualitative evidence on barriers to the use of dermoscopy which may be found in non-RCT study designs. We chose to start the search from 1990 as this was when the earliest dermoscopy-related research emerged. We considered published evidence from any international healthcare system and whether it could be interpreted and applied to primary care settings, including the extent to which data collected from specialist clinic settings could be applied to the lower-prevalence primary care population.

We included all studies which provide evidence around test accuracy, utility, acceptability to patients and PCPs, training requirements, and cost-effectiveness of dermoscopy modes in primary care; including trials, diagnostic accuracy and acceptability studies. As our interest was in use of dermoscopy by generalist clinicians, we included all studies reporting PCP use of dermoscopy; studies of secondary care physicians who were not trained in dermoscopy were assessed for the applicability of their study to answer the research question. We excluded studies that were based in any clinical setting other than at the first assessment of suspicious skin lesions, and any studies that were not considered primary studies.

Following duplicate removal, one author (OTJ) screened titles and abstracts to identify studies which fitted the inclusion criteria. 10% of the titles and abstracts were checked by two other authors (LJ and SH), and inter-assessor reliability was excellent, with disagreement for only one out of the 100 papers checked. Any disagreements were discussed by the core research team (OTJ, LJ, SH, FMW) and a consensus reached. At least two reviewers (OTJ, LJ, SH, MvM, FMW) independently assessed each full-text article for the possibility of inclusion in the review. Any disagreements were resolved by consensus-based discussion.

Data extraction was undertaken by two reviewers independently (OTJ, LJ, FMW) and summarised using descriptive tables, discussion and consensus. We chose to extract only reported outcomes from the included papers, without calculating further quantitative measures of diagnostic accuracy from their data, unless already reported. Due to the heterogeneity of the included papers we were not able to undertake any meta-analysis; instead, we chose to perform a narrative synthesis.

For peer review only - http://bmjopen.bmj.com/site/about/guidelines.xhtml

Risk-of-bias assessment was undertaken for each full-text paper by two independent researchers (OTJ, LJ) using the Joanna Briggs Institute Critical (JBI) Appraisal Tools.[22] These tools incorporate various critical assessments for different study designs, including patient selection, randomisation, data collection and analysis. As assessments for different study designs had varying denominators, the score was converted to a percentage and classified as high, medium and low risk to aid clarity of presentation and interpretation. Although the studies demonstrated a wide range in quality, no studies were excluded based upon their risk-of-bias assessment. Full details of our review question, search strategy, inclusion/exclusion criteria, methodology for data extraction, risk-of-bias assessment and outcomes extraction are described in **Appendices 1 and 2** (see Supporting Information), as well as a full list of excluded studies (**Appendix 3**).

Patient and Public Involvement

Our longstanding collaborator, Mrs Margaret Johnson, is a patient advocate. She commented regularly on the study from its conception, including aspects of the research question, outcome measures and study design. There was no patient recruitment required for this study. The results will be disseminated to patient advocates, groups, and relevant charities.

Results

Figure 1 shows the study PRISMA diagram. 837 studies were identified, of which 349 were duplicates. 95 articles underwent full-text review and 23 met the inclusion criteria.[14, 23-44] These 23 articles reported data relating to 49,769 lesions and 3,708 PCPs.

Table 1 provides a summary of the study characteristics for included studies. We included three randomised controlled trials (RCT), two sequential intervention trials (SIT), nine diagnostic accuracy studies, two cohort studies, two case series, one case-control study, and four PCP surveys. Table 1 also visually summarises the practitioner and patient populations reported in the studies and highlights the paucity of studies reporting PCPs using dermoscopy with primary care patients (5 out of 16). Studies of teledermoscopy-based referral systems were more frequently set in primary care with 6 out of 7 studies involving primary care clinicians and primary care patients. Overall, 16 of the 23 papers reported studies of PCPs, but only 11 papers reported studies involving primary care patients.

For peer review only - http://bmjopen.bmj.com/site/about/guidelines.xhtml

Table 1: Study demographics - patient and practitioner populations

Study details	Location	Study Type	Practitioners: PCPs	Practitioners: SCPs	Practitioners: PCPs & SCPs	Practitioners: DPCSCCs	Patients – Studies with patients from: Primary care	Patients – Studies with patients from: Secondary care	Patients – Studies with patients from: DPCSCCs	Patients – Studies using images from: Primary care	Patients – Studies using images from: Secondary care	Patients – Studies using images from: DPCSCCs	Control group	Intervention group	Gender of patients (except where indicated) (Female [F]/ Male [M])	Age of patients (except where indicated) (mean (years), range)
Dermoscopy papers																
Ahmadi *et al*. 2017[27]	Maastricht/ Limburg, Netherlands	Case series	■				■						None	Patients from 3 primary care practices	F=57.8%	54.7 60-79
Argenziano *et al*. 2006[23]	Barcelona, Spain. Naples, Italy	RCT	■				■						Naked eye	Dermoscopy	C: F=62.4% I: F=62.3%	C: 40, 2-90 I: 41, 3-94
Bourne *et al*. 2012[28]	Brisbane, Australia	DA study	■									■	Clinical assessment & algorithms	BLINK algorithm	F=52.2%	58 30-60
Chappuis *et al*. 2016[38]	4 regions of France	Survey	■										N/A	PCPs in France	GPs: F=42.4%	GPs: <30=8, 30-50=169 >50yrs=246
Koelink *et al*. 2014[24]	Groningen, Netherlands	RCT	■				■						Naked eye	Dermoscopy	C: F=61.6% I: F=68.2%	C: 54.7 I: 53.2
Menzies *et al*. 2005[29]	US, Germany & Australia	DA study			■							■	Independent clinicians	SolarScan assessment	N/R	N/R
Menzies *et al*. 2009[14]	Perth, Australia	SIT	■				■						PCP decision before intervention	Outcome after dermoscopy & SDDI	N/R	N/R
Morris *et al*. 2017 (a)[39]	Florida, USA	Survey	■										None	PCPs	Clinicians: F=41.6%	Clinicians: Median 40-49yrs
Morris *et al*. 2017 (b)[40]	Florida, USA	Survey			■								None	Practicing physicians	Clinicians: F=34.7%	Clinicians: N/R
Pagnanelli *et al*. 2003[30]	Rome, Italy	DA study		■ *							■		Pre-training	Post-training	N/R	N/R
Rogers *et al*. 2016[31]	New York, US	DA study			■						■		Histology/ expert opinion	Clinicians using 3 algorithms	F=53.3%	Median 31-40yrs

For peer review only - http://bmjopen.bmj.com/site/about/guidelines.xhtml

Rogers *et al.* 2017[32]	New York, US	DA study											Histology/ expert opinion	Clinicians using 3 algorithms	F=53.3%	Median 31-40yrs
Rosendahl *et al.* 2011[43]	Queensland, Australia	SIT	**										Naked-eye	Dermoscopy images	F=32.6%	57 SD: 17yrs
Rosendahl *et al.* 2012[25]	Australian SCARD database	Cohort study											Histology diagnosis	PCP decision	N/R	N/R
Secker *et al.* 2017[44]	Leiden, Netherlands	DA study											PCPs before education	After education	F=51.8%	45.2 28-63
Westerhoff *et al.* 2000[33]	Sydney, Australia	DA study											PCP diagnosis	PCPs +/- Dermoscopy +/- education	N/R	N/R
Teledermoscopy papers																
Borve *et al.* 2015[34]	Gothenburg, Sweden	Case control study											Paper-based referrals	Teledermoscopy referrals	C: F=57.1% I: F=61.4%	C: 61, 18-97 I: 54, 18-93
Ferrandiz *et al.* 2017[26]	Andalucia, Spain	RCT											Clinical images	Clinical & Dermoscopy images	C: F=52.88% I: F=62.28%	C: 57.33 I: 54.96
Grimaldi *et al.* 2009[35]	Siena, Italy	DA study											Judgement before dermoscopy	Judgement after dermoscopy	N/R	N/R
Livingstone *et al.* 2015[41]	Ruislip, UK	Case series											Expert Diagnosis & standard costs	Teledermoscopy referrals	N/R	N/R
Moreno-Ramirez *et al.* 2006[36]	Sevilla, Spain	DA study											Tele-dermatology referrals	Same patients + dermoscopy images	F=70.5%	38.8 1-73
Stratton *et al.* 2016[42]	Arizona, US	Survey											None	Nurse practitioners	Nurse Practitioners: F=92%	Nurse Practitioners: 48
Van der Heijden *et al.* 2013[37]	Amsterdam, Netherlands	Cohort study											Face-to-face consult +/- histology	Teledermoscopy consult (same patients)	F=55%	Median 47yrs 6-84yrs

* = minimal dermoscopy experience although secondary care physicians. ** = practitioner population not specified, DA study = Diagnostic Accuracy study, RCT = Randomised Controlled Trial, SIT = sequential intervention trial, PCP = Primary Care Practitioner, SCP = Secondary Care Practitioner, DPCSCC = Dedicated Primary Care Skin Cancer Clinic, SDDI = short-term sequential digital dermoscopy imaging, SCARD = Skin Cancer Audit Research Database, PSL = pigmented skin lesion, C group = control group, I group = intervention group

For peer review only - http://bmjopen.bmj.com/site/about/guidelines.xhtml

Table 2 summarises the outcome measures of each included study, grouped into accuracy and reliability outcomes and implementation outcomes, and shows the heterogeneous nature of the reported outcomes. The accuracy and reliability outcomes were diverse; twelve papers reported sensitivity and specificity, eight reported diagnostic accuracy or area-under-the-curve, five reported positive and negative predictive values (PPVs and NPVs), fourteen reported the proportion of correct decisions, four reported the number needed to excise, and five reported the biopsy rate. The implementation outcomes were less numerous but also quite diverse: four papers reported on PCP opinions, three performed cost-effective analyses, two looked at response times for teledermoscopy services, two looked at image quality for teledermoscopy, and one assessed patient satisfaction.

Risk-of-bias outcomes from the JBI critical appraisal tools are included in **Table 2** demonstrating a wide range in quality across the studies. No studies were excluded based upon the risk-of-bias assessment.

For peer review only - http://bmjopen.bmj.com/site/about/guidelines.xhtml

Table 2: Diversity of reported outcomes and critical appraisal results of the included studies

Study	Accuracy and Reliability Outcomes									Implementation Outcomes					JBI Critical Appraisal Checklists[22]					
	Sens & Spec	DA / AUC	PPV &NPV	Correctly diagnosed lesions	NNE	Biopsy rate	Inter-observer agreement	Inter-instrument reliability	Odds Ratio/ Relative Risk	Survey/ PCPs opinion	Cost-effective analysis	Response time for TDS	Patient Satisfaction	Image quality for TDS	Diagnostic Test Accuracy Studies	RCTs	Analytical Cross-Sectional Studies	Quasi-Experimental Studies	Case Series	Cohort Studies
Randomised Controlled Trials and Sequential Intervention Trials																				
Argenziano *et al.* 2006[23]	■		■	■												30-60%				
Koelink *et al.* 2014[24]		■		■		■			■		■					30-60%				
Menzies *et al.* 2009[14]	■		■	■	■	■												>60%		
Rosendahl *et al.* 2011[43]	■	■		■											>60%					
Ferrandiz *et al.* 2017[26]	■	■	■	■					■		■					>60%				
Non-RCT diagnostic accuracy studies																				
Ahmadi *et al.* 2017[27]			■	■		■													>60%	
Bourne *et al.* 2012[28]	■	■		■	■	■									>60%					
Menzies *et al.* 2005[29]	■	■	■	■				■							30-60%					
Pagnanelli *et al.* 2003[30]	■	■					■											>60%		
Rogers *et al.* 2016[31]	■														30-60%					
Rogers *et al.* 2017[32]	■			■											30-60%					
Rosendahl *et al.* 2012[25]					■														>60%	
Secker *et al.* 2017[44]	■			■														30-60%		
Westerhoff *et al.* 2000[33]	■																	>60%		
Borve *et al.* 2015[34]				■								■								30-60%
Grimaldi *et al.* 2009[35]				■	■	■												>60%		
Livingstone *et al.* 2015[41]											■	■	■						30-60%	
Moreno-Ramirez *et al.* 2006[36]	■	■		■										■				>60%		
Van der Heijden *et al.* 2013[37]		■		■			■							■				>60%		
Survey-based studies																				
Chappuis *et al.* 2016[38]										■							>60%			
Morris *et al.* 2017 (a)[39]										■							<30%			
Morris *et al.* 2017 (b)[40]										■							<30%			
Stratton *et al.* 2016[42]										■							30-60%			

Sens= sensitivity, spec=specificity, DA=diagnostic accuracy, AUC=area-under-the-curve, PPV=positive predictive value, NPV=negative predictive value, NNE=number-needed-to-excise, PCP=primary care practitioner, TDS=teledermoscopy, JBI=Joanna Briggs Institute, RCTs=randomised controlled trials

Key to JBI score: >60% | 30-60% | <30%

For peer review only

Table 3 summarises the diagnostic accuracy results, with the studies grouped into RCTs and SITs, non-RCT diagnostic accuracy studies, and survey studies. Among the RCTs and SITs, Argenziano *et al*.[23], Koelink *et al.*[24], Rosendahl *et al.*[43] and Menzies *et al.*[14] found that dermoscopy reduced the Number Needed to Excise to diagnose a melanoma (NNE). Ferrandiz *et al.*[26] evaluated the impact of adding dermoscopic images to the standard teledermatology referral system and found that it improved accuracy and confidence in diagnosing skin lesions.

Most of the studies were non-RCT diagnostic accuracy studies. These showed increased diagnostic accuracy with the use of dermoscopy in primary care, [28, 33, 35, 43, 44] or in teledermoscopy-based referral systems.[34-37] Some studies suggested this was due to improved ability to identify benign lesions when using a dermatoscope.[27, 31, 32, 44] All studies that assessed the effect of training found that it improved diagnostic accuracy compared to minimal or no training.[25, 29-33, 44]. There was evidence that use of dermoscopy without training displayed similar diagnostic accuracy to naked-eye examination. [33] Menzies *et al.*[29] showed that a dermoscopy-related technology, Solarscan, had higher sensitivity than PCPs although this was a non-significant finding.

For peer review only - http://bmjopen.bmj.com/site/about/guidelines.xhtml

Table 3(a): Summarised results of the included Randomised Controlled Trials (RCTs) and Sequential Intervention Trials (SITs)

Study	Summary	Control (C) / Interve-ntion (I) (Number of lesions)	Outcome measures: Healthcare professional (HCP) diagnosis	Outcome measures: Expert/Histopathology diagnosis	Outcome measures: Sensitivity (sens) / Specificity (spec)	Outcome measures: Positive predictive value (PPV) / Negative predictive value (NPV)	Outcome measures: Others
Randomised Controlled Trials and Sequential Intervention Trials							
Argenziano *et al*. 2006[23]	RCT in primary care comparing Primary Care Practitioners (PCPs) using naked eye observation (ABCD) to PCPs using dermoscopy (3-point checklist)	**C**<br>(1325)	Non-susp=925<br>Susp=408	39 susp, 23 malig<br>46 susp, 30 malig	Sens 54.1%<br>Spec 71.3%	PPV 11.3%<br>NPV 95.8%	2/6 MMs (malignant melanomas) missed
		**I**<br>(1203)	Non-susp =824<br>Susp=379	16 susp, 6 malig<br>61 susp, 33 malig	Sens 79.2%<br>Spec 71.8%	PPV 16.1%<br>NPV 98.1%	1/6 MMs missed
Koelink *et al*. 2014[24]	Cluster RCT in primary care comparing PCP diagnosis with naked-eye examination and dermoscopy examination	**C**<br>(230)	Non-susp =67<br>Referred=20<br>Biopsy/excision=135	Correctly diagnosed:<br>MMs 22.2% (2/9)<br>Lesions 40.5% (90/222)			All lesions odds ratio (OR)=1.51<br>Relative Risk=1.25<br>MM odds ratio=5.52
		**I**<br>(207)	Non-susp =84<br>Referred=18<br>Biopsy/excision=92	Correctly diagnosed:<br>MMs 61.5% (8/13)<br>Lesions 50.5% (98/194)<br>3 skin cancers incorrectly treated			
Menzies *et al*. 2009[14]	SIT using within-lesion controls in primary care assessing effect of dermoscopy & short-term sequential digital dermoscopy imaging (SDDI) on management of suspicious pigmented skin lesions (PSLs) by primary care practitioners (PCPs)	**C**<br>(374)	374 pigmented skin lesions suspicious for referral +/- excision	42 malignant lesions<br>33 MM, 1 MM in situ	**For MM:**<br>**Naked-eye:** Sens 37.5 Spec 84.6 PPV 20.7 NPV 92.7		56.4% reduction in suspicious PSLs excised/referred. 63.5% reduction in benign excised PSLs. 1MM in situ incorrectly managed in intervention group.<br>Benign:MM ratio of excised/referred lesions 9.5:1 vs 3.7:1 (after dermoscopy) vs 3.5:1 (dermoscopy + SDDI) (p<0.0005)
		**IA**<br>(374)	After dermoscopy<br>110 referred, 192 SDDI,<br>72 observed for change		**Dermoscopy:** Sens 53.1 Spec 89.0 PPV 34 NPV 94.7		
		**IB**<br>(192)	Of 192 SDDI: 46 referred/excised, 6 continued SDDI (2 subsequently referred, 4 observed),<br>140 observed (5 subsequently referred, 135 observed)		**+ SDDI:** Sens 71.9 Spec 86.6 PPV 36.4 NPV 96.6		
					**Overall:**<br>- Increased sens for all malignancies (40 to 67.5%,		

For peer review only - http://bmjopen.bmj.com/site/about/guidelines.xhtml

					p=0.014) and MM (37.5 to 71.9%, p=0.006) following intervention<br>- Increased PPV for MM (20.7 to 36.4%, p=0.055) and NPV for MM (92.7 to 96.6%, p=0.041)		
Rosendahl et al. 2011[43]	SIT using within lesion controls. Comparison of PSL diagnosis of ‘blinded observers’ using macroscopic images then dermoscopy images	**C**<br>(463)	Single best diagnosis matched histopathology diagnosis in 320 cases (69.1%)	29 MMs<br>72 BCCs<br>5 SCCs<br>NB: all PSLs excised	to achieve 80% spec, sensitivity 70.5%		AUC 0.83 [malignant neoplasms]<br>AUC 0.71 [melanocytic lesions]
		**I**<br>(463)	Single best diagnosis matched histopathology diagnosis in 375 cases (80.1%) [P<0.001]		to achieve 80% spec, sensitivity 82.6% (N.S.)		AUC 0.89 [malignant neoplasms] (P<0.001)<br>AUC 0.76 [melanocytic lesions] (N.S.)
Ferrandiz *et al*. 2017[26]	RCT comparing diagnostic accuracy (DA) & cost-effectiveness of clinical teleconsultations with clinical + dermoscopic teleconsultations from 5 primary care centres	**C**<br>(226)	70.36% non-susp<br>45.14% referred for face-to-face evaluation	2.77% MM<br>11.54% non-MM skin Ca	Sens 86.57<br>Spec 72.33	PPV 56.98<br>NPV 92.86<br>Accuracy index 79.20%	False Negative rate 13.43%<br>False Positive rate 22.16%<br>69.71% decisions made with higher confidence
		**I**<br>(228)	73.24% non-susp<br>20.18% referred for face-to-face evaluation (p<0.001)	2.19% MM<br>7.89% non-MM skin Ca	Sens 92.86<br>Spec 96.24	PPV 84.38<br>NPV 98.17<br>Accuracy index 94.30% (P<0.001)	False Negative rate 7.14%<br>False Positive rate 3.76%<br>78.07% decisions made with higher confidence (p=0.001)

v only

For peer review only - http://bmjopen.bmj.com/site/about/guidelines.xhtml

Table 3(b): Summarised results of the included non-RCT diagnostic accuracy studies

Study	Summary	Intervention or group (Number of lesions)	Outcome measures				
			Healthcare (HCP) diagnosis	Expert review	Sensitivity (sens) / Specificity (spec)	Positive predictive value (PPV) / Negative predictive value (NPV)	Others
Non-RCT diagnostic accuracy studies							
Ahmadi *et al*. 2017[27]	Retrospective cross-sectional study of medical files from 3 general practices	(580)	67 malignant, 75 pre-malignant, 399 non-suspicious, 39 unknown. 16.7% of patients referred.	151 lesions confirmed by histopathology/ dermatology: 37 BCC, 4 MMs, 1 lentigo maligna, 20 unknown	PPV: benign lesions 85.7%, pre-malignant lesions 18.2%, malignant lesions 53.8%, BCCs 53.3%, melanoma 25%		Tools used: dermoscopy 8.4%, experienced PCP advice 1.4%, biopsy 1.9%, excision 10.3%
Bourne *et al*. 2012[28]	Sequential design DA study. 4 PCPs used new BLINK dermoscopy algorithm on 50 lesion images, compared to same PCPs using 3-point checklist, Menzies, and Clinical Assessment on same 50 images	**BLINK** (200)	Found 33/36 MMs Biopsied 131/200 lesions	Images of 50 lesions used: 1 invasive MM (0.52mm), 8 in situ	Sens 90.8% Spec 50%		Diagnostic Accuracy 65.5% Number-needed-to-excise 6
		**3PCL** (200)	Found 19/36 MMs Biopsied 105/200 lesions		Sens 59.4% Spec 42.2%		Diagnostic Accuracy 48.3% Number-needed-to-excise 11
		**Menzies** (200)	Found 16/36 MMs Biopsied 71/200 lesions		Sens 54.7% Spec 69%		Diagnostic Accuracy 63.9% Number-needed-to-excise 13
		**Clinical** (200)	Found 9/36 MMs Biopsied 74/200 lesions		Sens 52.6% Spec 74.8%		Diagnostic Accuracy 65% Number-needed-to-excise 22
Menzies *et al*. 2005[29]	Sequential design DA study comparing the performance of SolarScan to that of clinicians with varying	**Dermatolo-gist** (78)	10.5		Diagnosing MM: Sens 81 Spec 60 PPV 30 NPV 94. Decision to excise: Sens 79 Spec 60 PPV 29 NPV 93		

	dermatology experience on 78 images of pigmented skin lesions	**PCP** (78)	8		Diagnosing MM: Sens 62 Spec 63 PPV 26 NPV 89. Decision to excise - Sens 62 Spec 61 PPV 25 NPV 89	
		**SolarScan** (78)	11.1		Diagnosing MM - Sens 85 Spec 65 PPV 32 NPV 96. Decision to excise - Sens 85 Spec 65 PPV 32 NPV 96	SolarScan's sensitivity was higher than PCPs but N.S.
Pagnanelli *et al*. 2003[30]	Sequential design DA study to assess if internet-based course suitable to teach dermoscopy to 16 clinicians with minimal dermoscopy experience looking at 20 images of PSLs	**Control** Pre-training (20)		20 pigmented skin lesions in test set: 6MMs, 14 non-MMs	**Pattern analysis:** Sens 67.7, Spec 76.3	Diagnostic accuracy 49.8 k-intraobserver agreement 0.42
					**ABCD:** Sens 58, Spec 73.4	Diagnostic accuracy 38.8 k-intraobserver agreement 0.31
					**7-point checklist:** Sens 100, Spec 67.5	Diagnostic accuracy 60.2 k-intraobserver agreement 0.58
					**Menzies method:** Sens 80.4, Spec 72	Diagnostic accuracy 53.5 k-intraobserver agreement 0.50
		**Intervention** Post-training (20)			**Pattern analysis:** Sens 82, Spec 78.5	Diagnostic accuracy 60.1 k-intraobserver agreement 0.58
					**ABCD:** Sens 78.4, Spec 79.6	Diagnostic accuracy 56.6 k-intraobserver agreement 0.55
					**7-point checklist:** Sens 100, Spec 69.8	Diagnostic accuracy 64.5 k-intraobserver agreement 0.61
					**Menzies method:** Sens 93.4, Spec 76	Diagnostic accuracy 62.8 k-intraobserver agreement 0.66
Rogers *et al*. 2016[31]	Sequential design DA study examining performance of new Triage Amalgamated Dermoscopic Algorithm (TADA) when used by 120 clinicians of various specialties looking at 50 pigmented skin lesion images	(50)	5641 lesion evaluations performed: 3034 malignant, 2607 non-suspicious	50 lesion images in test set, 23 benign, 27 malignant. Sens & Spec calculated for malignant lesions	Dermatologists: Sens 94.8%, Spec 78.5%	
					Non-dermatologists: Sens 93.7%, Spec 72.1%	
					>1yr dermoscopy experience: Sens 95.4%, Spec 77.3%	
					<1yr dermoscopy experience: Sens 91.3% Spec 74.2%	
Rogers *et al*. 2017[32]	Sequential design DA	(50)	No data for 3-point checklist and	50 lesion images in	TADA: Sens 94, Spec 75.5	Sens for MM with TADA 94%.

	study comparing performance of new Triage Amalgamated Dermoscopic Algorithm (TADA) to existing dermoscopy algorithms when used by 120 clinicians of various specialties		AC rule.<br>TADA: 5646 lesions evaluated. 2056 deemed non-suspicious [1891 True negatives (92.0%), 165 False negatives (8.0%)]<br>3590 deemed malignant [2871 True positives (80.0%), 719 False positives (20.0%)]	test set, 23 benign, 27 malignant. Sens & Spec based on 40 non-pigmented skin lesions	AC: Sens 88.6, Spec 78.7<br>3-point checklist: Sens 71.9, Spec 81.4<br>Untrained (using TADA): Sens 93.6, Spec 69<br>Trained (using TADA): Sens 95.4, Spec 73.2	Spec for untrained clinicians for benign pigmented skin lesions using TADA 76% - 94% (beginners can be quickly trained to identify benign lesions)
Rosendahl *et al*. 2012[25]	Prospective cohort study using Skin Cancer Audit Research Database (SCARD) to assess impact of dermoscopy use and subspecialisation on Malignant Melanoma diagnosis by PCPs	**Dedicated Skin Cancer Practitioners**	No of lesions seen not recorded on SCARD<br>11,992 lesions referred	11.7% were MM		MM number needed to treat: 8.5
		**PCP with Special Interest in Skin Cancer**	1942 lesions referred	10.6% MM		MM number needed to treat: 9.4
		**PCPs**	1942 lesions referred	5.9% MM		MM number needed to treat: 17.0
		**High dermoscopy use**	17,917 lesions referred	11.2% MM		MM number needed to treat: 8.9
		**Medium use**	2657 lesions referred	9.1% MM		MM number needed to treat: 10.9
		**Low use**	1093 lesions referred	6.9% MM		MM number needed to treat: 14.6<br>(P<0.0001, but N.S. when adjusted for subspecialisation)
Secker *et al*. 2017[44]	Sequential design DA study comparing performance of 293 PCPs in diagnosing pigmented skin lesions before and after a training intervention	Pre-test (clinical images, no education) (20)		20 pigmented skin lesion images in test set: 3MM, 2BCC, 15 benign	For MM:<br>Sens 0.49, Spec 0.75	% correct treatment:<br>i) malignant 85.87<br>ii) naevi 92.83<br>iii) benign 9.56
		Post-test (clinical images with education) (20)			Sens 0.50, Spec 0.77	i) malignant 84.03<br>ii) naevi 95.61<br>iii) benign 7.81

For peer review only - http://bmjopen.bmj.com/site/about/guidelines.xhtml

		Integrated Post-test (clinical and dermoscopic images with education) (20)			Sens 0.66, Spec 0.70	i) malignant 91.74<br>ii) naevi 92.35<br>iii) benign 29.35
		Overall			Training improved sens & spec for all except pigmented naevi. Training improved diagnostic accuracy for all pigmented skin lesions except naevi	Increase in correct treatments for benign lesions, reduced unnecessary referrals & excisions
Westerhoff *et al*. 2000[33]	Intervention study assessing performance of 74 PCPs with no dermoscopy experience in diagnosing 100 pigmented skin lesions using macroscopic images +/- dermoscopy images before and after an educational intervention	**Control**<br>No education (100)		100 images of lesions used: 50 invasive MMs and 50 non-melanomas	**Macroscopic images**:<br>Pre-test:<br>Sens 50.6%, Spec 55.2%<br>Post-test:<br>Sens 53.7%, Spec 51.5%<br>**+ dermoscopic images:**<br>Pre-test:<br>Sens 52.9%, Spec 58.1%<br>Post-test:<br>Sens 54.8%, Spec 55.8%	Significant improvement with training between pre-test (54.6%) and post-test (62.7%) (P=0.007) on macro images. Diagnosis of malignant melanoma with dermoscopy significantly better (75.9%) than macro images (62.7%) [P=0.000007]. No significant difference adding dermoscopic images in diagnosing non-malignant melanoma pigmented skin lesions
		**Intervention**<br>With education (100)			**Macroscopic images:**<br>Pre-test:<br>Sens 54.6%, Spec 53.0%<br>Post-test:<br>Sens 62.7%, Spec 53.6%<br>**+ dermoscopic images:**<br>Pre-test:<br>Sens 57.8%, Spec 55.5%<br>Post-test:<br>Sens 75.9%, Spec 57.8%	
Borve *et al*. 2015[34]	Case-control study. Smartphone Tele-dermoscopy system in 20 primary healthcare centres compared to	**Control**<br>Paper-based referrals (746)	746 suspicious lesions referred	323 malignant (13 MM, 7 MM in situ, 22 SCCs, 115 BCCs, 164 AKs)<br>423 benign		3/4 invasive MMs given medium/low priority, 3/5 MMs in situ given low priority. Mean response time 5 days (range 0-82 days). Patients received primary treatment

For peer review only - http://bmjopen.bmj.com/site/about/guidelines.xhtml

	traditional, paper-based referral system from other primary healthcare centres					on single face-to-face visit in 82.2% of cases.
		**Intervention** Smartphone Tele-dermoscopy referrals (816)	816 suspicious lesions referred. 346 (42%) non-suspicious, final diagnosis benign for 343 (3 malignant lesions missed were AKs). 196 deemed malignant, 146 (74%) also malignant after dermatology/HP	229 malignant (19MM, 16 MM in situ, 24 SCCs, 109 BCCs, 61 AKs) 587 benign		All invasive MMs prioritised correctly (high), all MM-in-situ at least medium priority. 22.6% more referrals given low priority. Mean response time 109 mins (range 2 mins-46hrs). Waiting time for surgical treatment for MM significantly shorter (p<0.0001). Patients received primary treatment on single face-to-face visit in 93.4% of cases.
Grimaldi *et al*. 2009[35]	Sequential design DA study assessing PCP diagnosis of suspicious pigmented skin lesions before and after dermoscopic evaluation and accuracy of tele-dermoscopy triage system.	**PCP Clinical** (235)	167 lesions non-suspicious, 68 suspicious	16 malignant (5 MMs), 219 benign.	Dermoscopy by PCPs and then experts led to 76.5% reduction in no. of surgical procedures (68 to 16)	PCP clinical vs PCP dermoscopy: P<0.001, OR 0.345731
		**PCP Dermoscopy** (235)	206 non-suspicious , 29 suspicious (dermoscopy corrected diagnosis in 57.3% of cases, only 1 False negative)			PCP clinical vs expert dermoscopy: P<0.001, OR 0.179425
		**Tele-dermoscopy** (235)	219 non-suspicious, 16 suspicious (1 False negative from 206 benign lesions)			PCP dermoscopy vs expert dermoscopy: P<0.05, OR 0.518973
Livingstone *et al*. 2015[41]	Prospective case series to assess cost-effectiveness, accuracy, and patient satisfaction of a tele-dermoscopy system for non-malignant pigmented skin lesions in a primary care practice.	(248)	248 patients that PCP would have been referred routinely to dermatology referred to tele-dermoscopy service. 102 needed face-to-face dermatology review. 146 advised on treatment. 3 lesions possibly malignant so referred 2-week-wait pathway	0/3 possibly malignant lesions were malignant at face-to-face review. None of other 245 lesions were malignant after review or follow-up		Waiting time for images to be taken (weeks): 0-1=27%, 1-2=45%, 2-3=7%, 3-4=0%, 4-5=7%, 5-6=7%.<br>Waiting time for results (weeks): 0-1=14%, 1-2=61%, 2-3=14%, 3-4=7%.<br>129 patients returned patient satisfaction questionnaires. 100% said tele-dermoscopy service was explained, 100% would recommend tele-dermoscopy

Moreno-Ramirez *et al.* 2006[36]	Sequential design DA study to assess if Tele-dermatology with dermoscopy images would improve the current Tele-dermatology-based triage system in referrals from a primary care centre	**Control** Teledermat-ology referrals (61)	4 BCCs, 1MM, dysplastic naevus 0, benign 56<br>Referral rates 47.5% (29)<br>Rate of referral of true positive results 17.2% (5 TPs/29 referrals)<br>False positives 58.7% (17/29 referrals)	Histopathology: 2 BCCs, 1MM, 1 dysplastic naevus, benign 57	Sens 1 (as 0 FNs)<br>Spec 0.65<br>False positive rate 0.35	Clinical picture quality excellent 41%, poor 3.3%. Average diagnostic confidence 4.14/5. Agreement with histology 0.91.
		**Intervention** Teledermos-copy referrals (61)	2 BCCs, 1MM, 1 dysplastic naevus, benign 54<br>Referral rates 39.3% (24) (P<0.05) Rate of referral of true positive results 20.8% (5 TPs/24 referrals) (P<0.05)<br>False positives 41.7% (10/24) (P<0.05)		Sens 1 (as 0 False negatives)<br>Spec 0.78 (P<0.05)<br>False positive rate 0.22 (P<0.05)	Dermoscopic picture quality excellent 63.9%, poor 6.6%. Average diagnostic confidence 4.75/5 (P<0.05). Agreement with histology 0.94.
Van der Heijden *et al.* 2013[37]	Cohort study assessing accuracy and reliability of tele-dermoscopy diagnosis with images taken by PCPs compared to diagnosis at face-to-face consultations for same lesions	**Control** Face to face assessment (76)	All 108 lesions also referred for face-to-face (F2F) assessment. 76 lesions seen face-to-face by dermatology. 32 not seen as: did not attend, moved away, GP did excision			Agreement F2F vs HP diagnosis k=0.90 (almost perfect), Diagnostic agreement k=0.56-0.78 (substantial), Management agreement k=0.31-0.38 (fair)
						Agreement TDS vs F2F diagnosis k=0.55-0.73 (moderate-substantial), TDS vs F2F management k=0.19-0.29 (fair). Image quality: 36% bad, 36% good. TDS consultations with good image quality had better agreement TDS vs F2F diagnosis (k=053-0.77, substantial), and TDS vs F2F management (k=0.34-0.47, fair-moderate)
		**Intervention** Teledermos-copy referrals (108)	108 lesions referred via tele-dermoscopy	Histopathology diagnosis for 36 lesions (33%). 2MMs and 5 non-melanoma skin cancers		Agreement TDS vs HP diagnosis k=0.41-0.63 (moderate). TDS consultations with good image quality had better agreement of TDS vs HP diagnosis (k=0.53-1.0, moderate-almost perfect)

For peer review only - http://bmjopen.bmj.com/site/about/guidelines.xhtml

Table 3(c): Summarised results of the included survey-based studies

Study	Summary	Population studied (N)	Outcomes
Survey-based studies			
Chappuis *et al.* 2016[38]	Survey of PCPs in 3 regions of France	PCPs (425)	Amongst dermoscopy users 21 (54%) had no training, 8 (21%) trained via books, 5 (13%) trained with dermatologist, 2 (5%) trained online. Lower referral rates in dermoscopy group.<br>Male PCPs significantly more likely to use a dermatoscope (P=0.001). PCPs >50 significantly more likely to use a dermoscopy (P<0.001). 30 (8%) had dermatoscope available, 16 (52%) used it >1x/week.
Morris *et al.* 2017(a) [39]	Descriptive cross-sectional survey of US physicians (Medical Doctors and Doctors of Osteopathy) to examine dermoscopy use and barriers	Family physicians (705)	Confidence recognising malignant lesions: Not confident=2.1%, little confident=18.8%, moderate=21.9%, confident=47.7%, very confident=9.4%<br>Currently using a dermatoscope associated with seeing >400 patients/month & >60yrs<br>Number of patients per month with suspicious lesions: <1.5 lesions=12.2%, 1.5-4.99 lesions=19.6%, 5-9.99 lesions=19.2%, 10-19.99 lesions=23.1%, >20 lesions=26%<br>Used a dermatoscope=19.5%. Currently use a dermatoscope=8.3%. Intention to start using in 12 months=63.6%
Morris *et al.* 2017(b) [40]	Same study but including all clinicians	Physicians (1466)	211 (14.6%) had used dermoscopy, 87 (6.0% of 1445) currently using, 656 (51.8%) intended to use in next 12 months. Use of and intention to use dermoscopy were associated with graduating recently, being a family physician, seeing a higher number of cancer patients and being more confident differentiating malignant and benign skin lesions.
Stratton *et al.* 2016[42]	Online survey, acceptance of mobile tele-dermoscopy by nurse Primary care practitioners in Arizona, US	Nurse practitioners (62)	Practitioners 40-60yrs & been in practice for 1-15 years scored higher for intention to use mobile tele-dermoscopy. Few nurse practitioners used mobile tele-dermoscopy. They scored highly for perceiving that mobile tele-dermoscopy would have a positive impact on their practice, they would find it interesting to use, they could easily learn mobile tele-dermoscopy, it would help with rapid diagnosis of skin cancer, and would improve the diagnosis of their patients, and they would use mobile tele-dermoscopy if they received training.

Legend: RCT=randomised controlled trial, 3PCL=3 point checklist, 7PCL=7 point checklist, N=no.=number, PPV=positive predictive value, NPV=negative predictive value, susp=suspicious, malig=malignant, MM=malignant melanoma, OR=odds ratio, C=control group, I=intervention group, sens=sensitivity, spec=specificity, PSL=pigmented skin lesion, SDDI=short-term sequential digital dermoscopy imaging, HP=histopathology, BCC=basal cell carcinoma, SCC=squamous cell carcinoma, AK=actinic keratosis, AUC=area under the curve, F2F=face-to-face, FP=false positive, FN=false negative, TP=true positive, TN=true negative, SIT=sequential intervention trial, derm=dermatology, NNE=number needed to excise, NNT=number needed to treat, DA=diagnostic accuracy, k-IA=k-intraobserver agreement, N.S.=not significant, TD=teledermatology, MTD=mobile teledermoscopy, TDS=teledermoscopy, GP=general practitioner, PCP=primary care practitioner, HCP=healthcare practitioner, DCSP=dedicated skin cancer practitioner, GPSISC=GP with special interest in skin cancer, Ca=cancer, 2ww=two week wait or urgent referral, SCARD=skin cancer audit research database

For peer review only - http://bmjopen.bmj.com/site/about/guidelines.xhtml

Table 4 summarises findings from the studies which investigated barriers and facilitators to implementing dermoscopy in primary care. Training requirements, cost of equipment and the time taken to perform dermoscopy were the most important barriers identified from the studies. However, for each barrier there were some papers that described it as a facilitator instead. Three papers performed cost-effective analyses of dermoscopy,[24] and teledermoscopy,[26, 41] none found a significant cost-effective advantage. The main facilitators identified to the use of dermoscopy in primary care were reduced referrals, early detection of melanoma and reduced patient and physician anxiety.

For peer review only

For peer review only - http://bmjopen.bmj.com/site/about/guidelines.xhtml

Table 4: Barriers and facilitators to implementation of dermoscopy and teledermoscopy

Aspect	Quoted as barrier in:	Type of Study	Quoted as facilitator in:	Type of Study
Training requirements	Chappuis *et al*. 2016[38]	Survey	Pagnanelli *et al*. 2003[30]	DA study
	Morris *et al*. 2017 (b)[40]	Survey		
	Van der Heijden *et al*. 2013[37]	Cohort study		
Cost<br>*=based on cost-effective analysis	Chappuis *et al*. 2016[38]	Survey	Koelink *et al*. 2014[24] *	RCT
	Morris *et al*. 2017 (a)[39]	Survey	Rosendahl *et al*. 2012[25]	Cohort study
	Moreno-Ramirez *et al*. 2006[36]	DA study	Ferrandiz *et al*. 2017[26] *	RCT
			Livingstone *et al*. 2015[41] *	Case series
Time consumption	Chappuis *et al*. 2016[38]	Survey	Borve *et al*. 2015[34]	Case-control
	Moreno-Ramirez *et al*. 2006[36]	DA study		
	Van der Heijden *et al*. 2013[37]	Cohort study		
Reimbursement for offering dermoscopy services (in US)	Morris *et al*. 2017 (a)[39]	Survey		
Equipment issues	Van der Heijden *et al*. 2013[37]	Cohort study	Borve *et al*. 2015[34]	Case-control
			Moreno-Ramirez *et al*. 2006[36]	DA study
Reduced referrals			Chappuis *et al*. 2016[38]	Survey
			Koelink *et al*. 2014[24]	RCT
			Borve *et al*. 2015[34]	DA study
			Moreno-Ramirez *et al*. 2006[36]	DA study
Early detection of melanoma			Chappuis *et al*. 2016[38]	Survey
Reduced patient anxiety			Chappuis *et al*. 2016[38]	Survey
Reduced physician anxiety			Chappuis *et al*. 2016[38]	Survey
			Moreno-Ramirez *et al*. 2006[36]	DA study
			Menzies *et al*. 2009[14]	DA study

Legend: DA=diagnostic accuracy, RCT=randomised controlled trial

For peer review only - http://bmjopen.bmj.com/site/about/guidelines.xhtml

Patient and PCP attitudes and acceptance of dermoscopy

Several papers assessed PCPs attitudes to dermoscopy through questionnaires. Stratton *et al.*[42] found that nurse practitioners in the US did not widely use dermatoscopes; however, they thought that dermoscopy would have a positive impact, and would be willing to use mobile teledermoscopy if they received training. Morris *et al.*[39, 40] found that dermoscopy use amongst US physicians and Doctors of Osteopathic Medicine was associated with seeing higher numbers of patients and with higher confidence in diagnosing skin lesions. Chappuis *et al.* [38] found that dermoscopy use amongst French GPs was associated with being older and male, and that only 8% of respondents had access to a dermatoscope. Livingstone *et al.'s* survey was the only one that assessed patient acceptability;[41] they reported that 97% of patients from one general practice in Greater London were satisfied with the teledermoscopy service and 100% would recommend it.

Discussion

Principal findings

Only a small number of studies have examined the use of dermoscopy or dermoscopy-related technologies in the primary care setting. These studies were all set in Europe, the US and Australia, and, due to their heterogeneous nature, we were not able to synthesise the findings. Nevertheless, our review found that, with appropriate training, dermoscopy in primary care is more accurate than naked-eye examination, with improvements in sensitivity and specificity and number needed to excise. Furthermore, there was some evidence that teledermoscopy-based referrals systems improve triage accuracy compared to paper-based or macroscopic image-based referral systems. The limited evidence did not show a significant cost-effectiveness benefit for either dermoscopy or tele-dermoscopy, although dermoscopy appears to lead to a reduction in unnecessary referrals and excisions. Importantly, the review also suggests that PCPs are receptive to incorporating dermoscopy into their routine practice, although they recognised ongoing implementation barriers, particularly around training, time requirements, and technology costs.

Comparison with other studies

Our review suggests that dermoscopy in primary care is more accurate than naked-eye examination, supporting the findings from a previous review of dermoscopy for melanoma detection specifically in primary care published in 2012.[45] A recently published Cochrane review of dermoscopy for the diagnosis of melanoma has also concluded that, although data to support dermoscopy use in primary care is limited, 'it may assist in triaging suspicious lesions for urgent referral when employed by suitably trained clinicians'.[19] Our review also suggests that training PCPs in dermoscopy improves diagnostic accuracy. Again, this finding is supported by the recent Cochrane review, which also suggests that 'formal algorithms may be of most use for dermoscopy training purposes and for less expert observers, however reliable data comparing approaches using dermoscopy in-person are lacking'.[19] Previous reviews have shown that using dermoscopy without training was no more accurate than naked-eye examination alone.[13, 19] However, we were not able to identify the optimal length of training needed to train PCPs to use dermoscopy accurately, although studies of the effect of training on dermatologist diagnostic performance have shown improvement after between 2 days (6 hours training per day),[46] and 10 weeks (comprising 6 workshops of 4-6 hours).[47] PCPs are likely to need short training courses, preferably with regular updates, as one of the few RCTs examining the impact of dermoscopy on the management of pigmented lesions in primary care reported a high drop-out rate of GPs from the 20-hour online training course.[14]

It is important to note that the performance of dermoscopy in specialist clinics is not directly translatable as evidence for the performance of dermoscopy in primary care settings. A spectrum effect or spectrum bias is often observed when tests developed in one population are then used on another population. For example, the secondary

For peer review only - http://bmjopen.bmj.com/site/about/guidelines.xhtml

care population is a referred population and has a higher prevalence of the condition being tested than primary care populations. This means that a diagnostic test, such as dermoscopy, will perform differently in the primary care population with the lower prevalence of the condition, compared to the secondary care population.[48] The direction of effect is not consistent across tests and conditions, hence to establish the performance of tests among the non-referred population in primary care they need to be evaluated in a primary care population. This review has therefore aimed to examine existing evidence for dermoscopy use in primary care settings.

Our review suggests that a range of PCPs, including nurse practitioners in the US, and PCPs in the US and France, hold positive views about incorporating dermoscopy into their routine practice. Evidence from Australia supports these views, and demonstrates that a wide range of PCPs are able to incorporate dermoscopy into their routine clinical practice.[16] Only a small number of cost-effectiveness studies met our review criteria. They all assessed dermoscopy and teledermoscopy from a healthcare perspective, and only reported on short-term costs resulting from dermoscopy or non-dermoscopy approaches. None reported a significant cost-effectiveness benefit for dermoscopy,[24] or tele-dermoscopy,[26] in the primary care setting, although they recommended the technologies as potentially useful tools. An English RCT of another diagnostic aid (MoleMate, incorporating SIAscopy) in primary care,[49] also reported equivocal findings on cost-effectiveness, as the device, similar in accuracy to systematic application of the 7-point checklist, resulted in increased referrals from primary care.[49, 50]

Interestingly, no papers reporting the use of dermoscopy smartphone applications ('apps') for automated diagnosis of melanoma or skin cancers in the primary care setting met the review inclusion criteria. Kassianos *et al.*[51] reviewed 39 smartphone applications and found little evidence of clinical or research-based input into the design or evaluation of these apps. A recent editorial in Lancet Oncology supported this finding,[52] and urged caution with early adoption of new technologies that are often poorly designed and untested, stressing the need to ensure that these technologies are appropriate, cost-effective and do not compromise patient safety. Recent studies have tested the application of artificial intelligence, neural networks and machine learning to the diagnosis of skin lesions; however, they have not yet been assessed in primary care settings.

Strengths, limitations and future research

Our review examines the evidence for dermoscopy use in the primary care setting. It builds on the recent Cochrane review, which explicitly reviewed evidence only about diagnostic accuracy of dermoscopy, with and without naked eye examination, and describes this in specialist and generalist settings.[19] We therefore included studies with a range of methods, surveys and qualitative studies as well as RCTs and diagnostic accuracy studies, but still only identified a relatively small number of publications. Unfortunately, we were unable to perform a meta-analysis due to the heterogeneity in study designs, settings, populations, and outcomes. All the studies are from high-income countries, and therefore may be less generalisable to other countries with different health care systems.

Conclusions

Despite the limited evidence, this review provides moderate support for the use of dermoscopy in primary care, with the weight of the available evidence pointing to a benefit in diagnostic accuracy for managing suspicious skin lesions. Dermoscopy is acceptable to PCPs, so it could help them triage suspicious lesions for urgent referral or reassurance. However, it will be important to establish further evidence on minimum for training to reach competence, as well as the cost-effectiveness and patient acceptability of implementing dermoscopy in primary care.

For peer review only - http://bmjopen.bmj.com/site/about/guidelines.xhtml

Acknowledgements

The authors thank Isla Kuhn, Reader Services Librarian, University of Cambridge Medical Library for her help in developing the search strategy, and Margaret Johnson, a patient advocate, who provided regular comments on the study from its conception.

Funding

This research arises from the CanTest Collaborative, which is funded by Cancer Research UK [C8640/A23385]. This work was also supported by FMW's Clinician Scientist award (RG 68235) from the National Institute for Health Research (NIHR). The views expressed in this publication are those of the authors and not necessarily those of the National Health Service, the NIHR or the Department of Health. The funding sources had no role in the study design, data collection, data analysis, data interpretation, writing of the report, or in the decision to submit for publication.

Contributors

OTJ developed the protocol, completed the search, screened articles for inclusion, extracted data, synthesised the findings, interpreted the results and drafted the manuscript. LCJ screened articles for inclusion, extracted data, and critically revised the manuscript. MAvM screened articles for inclusion, extracted data, and critically revised the manuscript. SH screened articles for inclusion and critically revised the manuscript. NPB developed the protocol and critically revised the manuscript. PNH developed the protocol and critically revised the manuscript. JE developed the protocol, interpreted the results, and critically revised the manuscript. FMW developed the protocol, synthesised the findings, interpreted the results, and critically revised the manuscript. All authors approved the final version.

Competing interests

Per Hall is Clinical Advisor for Skin Cancer to Check4Cancer a company that offers teledermatoscopic analysis of pigmented skin lesions (https://www.check4cancer.com); and is a medical advisor to MedX the Canadian company with the IP for the SIAscope (www.medxhealth.com/default.aspx). No other authors have any conflicts of interest to declare.

Data sharing statement

All data are available from the reports or authors of the primary research. No additional data are available.

For peer review only

For peer review only - http://bmjopen.bmj.com/site/about/guidelines.xhtml

References

1. Skin cancer statistics: International World Cancer Research Fund; 2018 [updated 2018-08-22. Available from: https://www.wcrf.org/dietandcancer/cancer-trends/skin-cancer-statistics accessed 05/02/2019.
2. Mistry M, Parkin DM, Ahmad AS, et al. Cancer incidence in the United Kingdom: projections to the year 2030. *Br J Cancer* 2011;105(11):1795-803. doi: 10.1038/bjc.2011.430
3. Hiom SC. Diagnosing cancer earlier: reviewing the evidence for improving cancer survival. *Br J Cancer* 2015;112 Suppl 1:S1-5. doi: 10.1038/bjc.2015.23
4. NICE. NICE NG14 melanoma assessment & management.pdf. UK: National Institute for Clinical Excellence, 2015.
5. Chuchu N, Dinnes J, Takwoingi Y, et al. Smartphone applications for the diagnosis of cutaneous melanoma in adults. *Cochrane Database of Systematic Reviews* 2018 (In Press)
6. Dinnes J, Deeks JJ, Chuchu N, et al. Reflectance confocal microscopy for the diagnosis of cutaneous melanoma in adults. *Cochrane Database of Systematic Reviews* 2018 (In Press)
7. Ferrante di Ruffano L, Dinnes J, Deeks JJ, et al. Optical coherence tomography for the diagnosis of skin cancer in adults. *Cochrane Database of Systematic Reviews* 2018 (In Press)
8. Ferrante di Ruffano L, Takwoingi Y, Dinnes J, et al. Computer assisted diagnosis techniques (dermoscopy and spectroscopy-based) for the diagnosis of skin cancer in adults. *Cochrane Database of Systematic Reviews* 2018 (In Press)
9. Dinnes J, Bamber J, Chuchu N, et al. High frequency ultrasound for the diagnosis of skin cancer in adults. *Cochrane Database of Systematic Reviews* 2018 (In Press)
10. Rosendahl C, Cameron A, McColl I, et al. Dermatoscopy in routine practice - 'Chaos and Clues'. *Australian Family Physician* 2012;41(7):482-87.
11. Argenziano G, Cerroni L, Zalaudek I, et al. Accuracy in melanoma detection: a 10-year multicenter survey. *J Am Acad Dermatol* 2012;67(1):54-9. doi: 10.1016/j.jaad.2011.07.019
12. Menzies SW, Chamberlain A, Soyer HP, et al. What is the role of dermoscopy in melanoma diagnosis? Sydney: Cancer Council Australia: Cancer Council Australia Melanoma Guidelines Working Party; 2018 [cited 2018 Aug 30th]. Available from: https://wiki.cancer.org.au/australia/Clinical_question:What_is_the_role_of_dermoscopy_in_melanoma_diagnosis%3F accessed Aug 30th 2018.
13. Vestergaard ME, Macaskill P, Holt PE, et al. Dermoscopy compared with naked eye examination for the diagnosis of primary melanoma: a meta-analysis of studies performed in a clinical setting. *Br J Dermatol* 2008;159(3):669-76. doi: 10.1111/j.1365-2133.2008.08713.x [published Online First: 2008/07/12]
14. Menzies SW, Emery J, Staples M, et al. Impact of dermoscopy and short-term sequential digital dermoscopy imaging for the management of pigmented lesions in primary care: A sequential intervention trial. *British Journal of Dermatology* 2009;161(6):1270-77.
15. Noor O, 2nd, Nanda A, Rao BK. A dermoscopy survey to assess who is using it and why it is or is not being used. *Int J Dermatol* 2009;48(9):951-2. doi: 10.1111/j.1365-4632.2009.04095.x [published Online First: 2009/08/26]
16. Chamberlain AJ, Kelly JW. Use of dermoscopy in Australia. *Medical Journal of Australia* 2007;187(4):252-53.
17. Vestergaard ME, Menzies SW. Automated diagnostic instruments for cutaneous melanoma. *Semin Cutan Med Surg* 2008;27(1):32-6. doi: 10.1016/j.sder.2008.01.001 [published Online First: 2008/05/20]
18. Haenssle HA, Fink C, Schneiderbauer R, et al. Man against machine: diagnostic performance of a deep learning convolutional neural network for dermoscopic melanoma recognition in comparison to 58 dermatologists. *Ann Oncol* 2018;29(8):1836-42. doi: 10.1093/annonc/mdy166 [published Online First: 2018/05/31]
19. Dinnes J, Deeks JJ, Chuchu N, et al. Cochrane Skin Cancer Diagnostic Test Accuracy Group. Dermoscopy, with and without visual inspection, for the diagnosis of melanoma in adults. *Cochrane Database of Systematic Reviews* 2018(10) doi: 10.1002/14651858.CD011902.
20. Stewart DM, Larissa S, Mike C, et al. Preferred reporting items for systematic review and meta-analysis protocols (PRISMA-P) 2015 statement. *Systematic Reviews* 2015;4(1):1. doi: doi:10.1186/2046-4053-4-1
21. Jones OT, Jurasceck L, Van Melle M, et al. Can dermoscopy be used accurately and effectively in primary care to appropriately triage possible melanoma? PROSPERO: CRD42018091395; 2018 [Available from: https://www.crd.york.ac.uk/prospero/display_record.php?RecordID=91395 accessed 15/08/2018.
22. Joanna Briggs Institute UoA. Joanna Briggs Institute Critical Appraisal Tools 2018 [Available from: http://joannabriggs.org/ accessed 30/05/2018.

For peer review only - http://bmjopen.bmj.com/site/about/guidelines.xhtml

23. Argenziano G, Puig S, Zalaudek I, et al. Dermoscopy improves accuracy of primary care physicians to triage lesions suggestive of skin cancer. *Journal of Clinical Oncology* 2006;24(12):1877-82.
24. Koelink C, Vermeulen K, Kollen B, et al. Diagnostic accuracy and cost-effectiveness of dermoscopy in primary care: a cluster randomized clinical trial. *Journal of the european academy of dermatology and venereology : JEADV* 2014; 28(11). http://onlinelibrary.wiley.com/o/cochrane/clcentral/articles/747/CN-01111747/frame.html.
25. Rosendahl C, Williams G, Eley D, et al. The impact of subspecialization and dermatoscopy use on accuracy of melanoma diagnosis among primary care doctors in Australia. *Journal of the American Academy of Dermatology* 2012;67(5):846-52.
26. Ferrandiz L, Ojeda-Vila T, Corrales A, et al. Internet-based skin cancer screening using clinical images alone or in conjunction with dermoscopic images: a randomized teledermoscopy trial. *Journal of the american academy of dermatology* 2017; (no pagination). http://onlinelibrary.wiley.com/o/cochrane/clcentral/articles/754/CN-01299754/frame.html.
27. Ahmadi K, Prickaerts E, Smeets JGE, et al. Current approach of skin lesions suspected of malignancy in general practice in the Netherlands: a quantitative overview. *Journal of the European Academy of Dermatology & Venereology* 2017;27:27.
28. Bourne P, Rosendahl C, Keir J, et al. BLINCK-A diagnostic algorithm for skin cancer diagnosis combining clinical features with dermatoscopy findings. *Dermatology Practical & Conceptual* 2012;2(2):202a12.
29. Menzies SW, Bischof L, Talbot H, et al. The performance of SolarScan: An automated dermoscopy image analysis instrument for the diagnosis of primary melanoma. *Archives of Dermatology* 2005;141(11):1388-96.
30. Pagnanelli G, Soyer HP, Argenziano G, et al. Diagnosis of pigmented skin lesions by dermoscopy: web-based training improves diagnostic performance of non-experts. *British Journal of Dermatology* 2003;148(4):698-702.
31. Rogers T, Marino ML, Dusza SW, et al. A clinical aid for detecting skin cancer: The triage amalgamated dermoscopic algorithm (TADA). *Journal of the American Board of Family Medicine* 2016;29(6):694-701.
32. Rogers T, Marino M, Dusza SW, et al. Triage amalgamated dermoscopic algorithm (TADA) for skin cancer screening. *Dermatology Practical & Conceptual* 2017;7(2):39-46.
33. Westerhoff K, McCarthy WH, Menzies SW. Increase in the sensitivity for melanoma diagnosis by primary care physicians using skin surface microscopy. *British Journal of Dermatology* 2000;143(5):1016-20.
34. Börve A, Dahlén Gyllencreutz J, Terstappen K, et al. Smartphone teledermoscopy referrals: A novel process for improved triage of skin cancer patients. *Acta Dermato-Venereologica* 2015;95(2):186-90. doi: 10.2340/00015555-1906
35. Grimaldi L, Silvestri A, Brandi C, et al. Digital epiluminescence dermoscopy for pigmented cutaneous lesions, primary care physicians, and telediagnosis: a useful tool? *Journal of Plastic, Reconstructive and Aesthetic Surgery* 2009;62(8):1054-58. doi: 10.1016/j.bjps.2008.01.011
36. Moreno-Ramirez D, Ferrandiz L, Galdeano R, et al. Teledermatoscopy as a triage system for pigmented lesions: a pilot study. *Clin Exp Dermatol* 2006;31(1):13-8. doi: 10.1111/j.1365-2230.2005.02000.x
37. van der Heijden JP, Thijssing L, Witkamp L, et al. Accuracy and reliability of teledermatoscopy with images taken by general practitioners during everyday practice. *Journal of Telemedicine and Telecare* 2013;19(6):320-25.
38. Chappuis P, Duru G, Marchal O, et al. Dermoscopy, a useful tool for general practitioners in melanoma screening: A nationwide survey. *British Journal of Dermatology* 2016;175:744-50.
39. Morris JB, Alfonso SV, Hernandez N, et al. Examining the factors associated with past and present dermoscopy use among family physicians. *Dermatology Practical & Conceptual* 2017;7(4):63-70.
40. Morris JB, Alfonso SV, Hernandez N, et al. Use of and intentions to use dermoscopy among physicians in the United States. *Dermatology Practical & Conceptual* 2017;7(2):7-16.
41. Livingstone J, Solomon J. An assessment of the cost-effectiveness, safety of referral and patient satisfaction of a general practice teledermatology service. *London Journal of Primary Care* 2015;7(2):31-35.
42. Stratton D, Loescher LJ. The acceptance of mobile teledermoscopy by primary care nurse practitioners in the state of Arizona. *Journal of the American Association of Nurse Practitioners* 2016;28(6):287-93.
43. Rosendahl C, Tschandl P, Cameron A, et al. Diagnostic accuracy of dermatoscopy for melanocytic and nonmelanocytic pigmented lesions. *Journal of the American Academy of Dermatology* 2011;64(6):1068-73.
44. Secker LJ, Buis PAJ, Bergman W, et al. Effect of a dermoscopy training course on the accuracy of primary care physicians in diagnosing pigmented lesions. *Acta Dermato-Venereologica* 2017;97(2):263-65.
45. Herschorn A. Dermoscopy for melanoma detection in family practice. *Canadian Family Physician* 2012;58(7):740-45.

For peer review only - http://bmjopen.bmj.com/site/about/guidelines.xhtml

46. Troyanova P. A beneficial effect of a short-term formal training course in epiluminescence microscopy on the diagnostic performance of dermatologists about cutaneous malignant melanoma. *Skin Research & Technology* 2003(9):269-73.
47. Stanganelli I, Seidenari S, Serafini M, et al. Diagnosis of pigmented skin lesions by epiluminescence microscopy: determinants of accuracy improvement in a nationwide training programme for practical dermatologists. *Public Health* 1999;113(5):237-42. doi: 10.1038/sj.ph.1900575
48. Juliet AU-S, Stephen JS, Simon JG. The spectrum effect in tests for risk prediction, screening, and diagnosis. *BMJ* 2016 doi: 10.1136/bmj.i3139
49. Wilson ECF, Emery JD, Louise Kinmonth A, et al. The cost-effectiveness of a novel siascopic diagnostic aid for the management of pigmented skin lesions in primary care: A decision-analytic model. *Value in Health* 2013;16(2):356-66.
50. Walter FM, Morris HC, Humphrys E, et al. Effect of adding a diagnostic aid to best practice to manage suspicious pigmented lesions in primary care: randomised controlled trial. *BMJ* 2012;345:e4110.
51. Kassianos AP, Emery JD, Murchie P, et al. Smartphone applications for melanoma detection by community, patient and generalist clinician users: A review. *British Journal of Dermatology* 2015;172(6):1507-18.
52. Editorial. Digital oncology apps: revolution or evolution? *Lancet Oncology* 2018;19(August 2018)

For peer review only

For peer review only - http://bmjopen.bmj.com/site/about/guidelines.xhtml

Supporting Information

Appendix 1 (Protocol for methods), **Appendix 2** (Search strategies) and **Appendix 3** (Full list of studies that underwent full text review, their assessors and outcomes) are available in the attached supporting information.

Figure Legends

Figure 1: PRISMA flow diagram for the studies included in the review.

For peer review only

For peer review only - http://bmjopen.bmj.com/site/about/guidelines.xhtml

Figure 1: PRISMA flow diagram for the studies included in the review

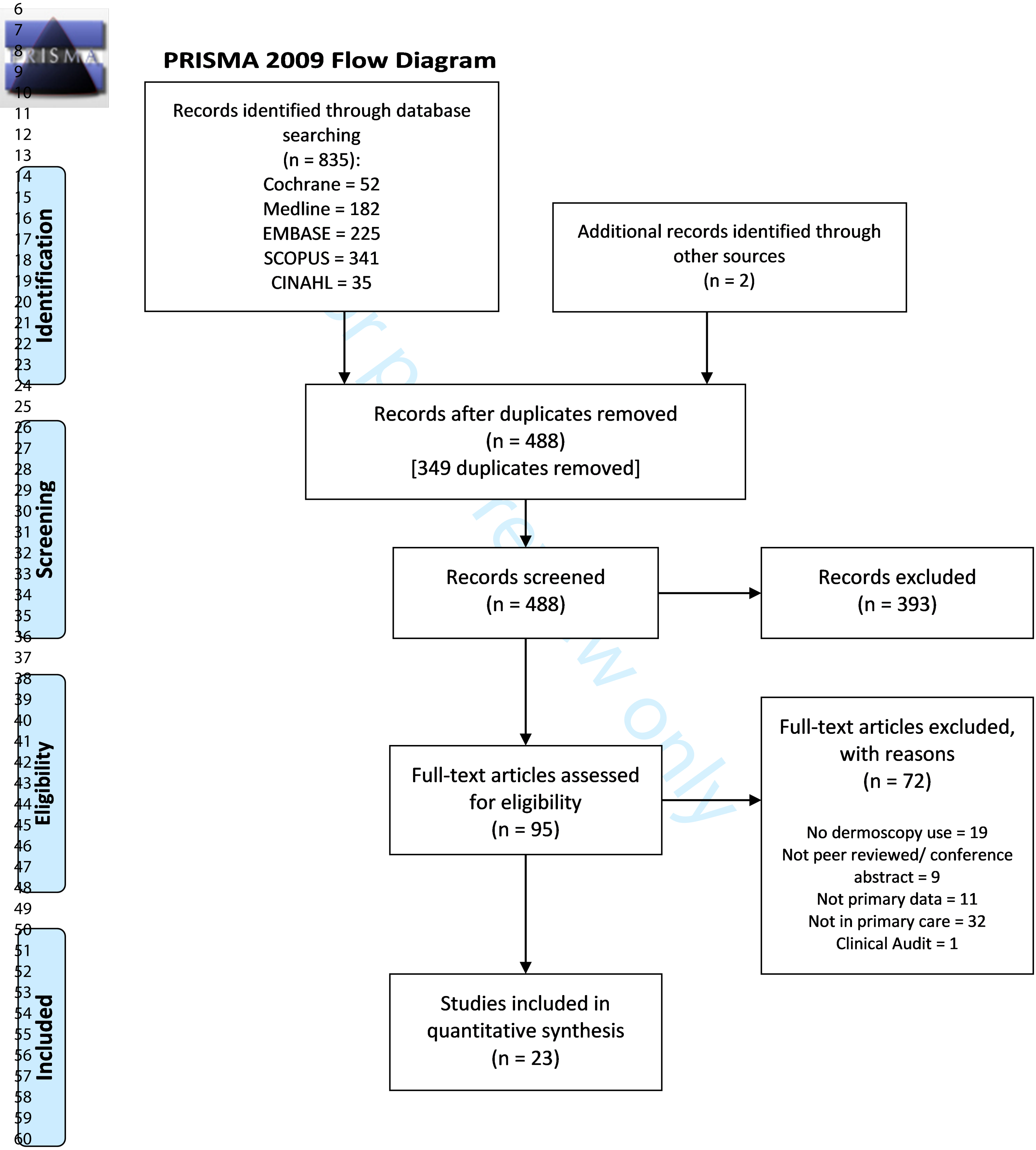

Supporting Information

Appendix 1 – Protocol for methods

Databases

We searched the following bibliographic databases from January 1990 to December 2017 for relevant published studies:

- Medline
- The Cochrane Central Register of Controlled Trials (CENTRAL) in the Cochrane Library
- EMBASE
- CINAHL (Cumulative Index to Nursing and Allied Health Literature via EBSCO)
- SCOPUS

Inclusion and exclusion criteria

The following inclusion criteria were applied:

i. All published literature from January 1990 to December 2017
ii. Which provide evidence around test accuracy, utility, acceptability to patients and GPs, and cost-effectiveness of dermoscopy modes in primary care
iii. Including trials, diagnostic accuracy and acceptability studies

The following exclusion criteria were also applied:

i. Studies based in any clinical setting other than at the first assessment of suspicious skin lesions (i.e. primary care in the UK)
ii. Studies involving specialist clinicians (in line with the Cochrane review of dermoscopy which stated 'although primary care clinicians can in practice be specialists in skin cancer, we considered primary care physicians as generalist practitioners and dermatologists as specialists'[1].)
iii. Studies that are not primary studies

Search Strategy

An electronic literature search of Medline, EMBASE, CENTRAL, CINAHL, and SCOPUS was performed from January 1990 to December 2017 using a combination of subject headings as detailed in Appendix Two. The validity of the search strategy was assessed by cross-checking articles identified in the search against:

i. the reference lists of key review articles[1-5]
ii. papers identified by a first and last author search of six key papers in the field[6-11]

The search was then further extended by manually screening the reference lists of all included papers.

Study Selection

The study selection followed these stages:

1. The titles and abstracts of the complete list of identified papers were independently assessed by one researcher (OTJ) against the inclusion and exclusion criteria. Those studies clearly not relevant were rejected at this stage.
2. One or two other members of the research team (SH, LJ) independently assessed a random selection of 10% of the papers that have been excluded, to check agreement between the reviewers.
3. For studies where a definite decision to reject could not be made based on title and abstract alone, the full paper was obtained for detailed assessment.
4. At least two reviewers (OTJ, FW, SH, LJ, MvM) independently assessed the full-text of included articles for the possibility of inclusion in the review. Papers identified as not meeting the inclusion criteria by both researchers were excluded.

For peer review only - http://bmjopen.bmj.com/site/about/guidelines.xhtml

5. The researchers met regularly to discuss papers in which it is unclear whether they meet the inclusion criteria or not and any disagreements were resolved by discussion with other members of the research team.

Data Extraction

Included papers had relevant information extracted and entered into a spreadsheet, including:

1. The characteristics of the study including:
    a. Study details
    b. Location of study
    c. Study design
    d. Study population
        i. Primary care, referred, secondary care
        ii. Control comparator
        iii. Total number of benign/suspicious lesions and number of patients
        iv. Gender
        v. Age
    e. Study methods
        i. Selection of participants
        ii. Exclusions
        iii. Follow-up time
        iv. Definition of the outcome
2. The outcomes of the study in terms of:
    a. Number of lesions deemed banal and suspicious in control and intervention groups
    b. Number of excised lesions and excised benign lesions
    c. Outcome Measure: Sensitivity/specificity
    d. Outcome Measure: Positive Predictive Value/Negative Predictive Value
    e. Outcome Measure: Others (e.g. diagnostic rate of new test, accuracy index etc.)
    f. Outcome Measure: specific to melanoma
    g. Stage of melanoma at diagnosis
    h. 5-year survival rate
3. Challenges for implementation in primary care:
    a. Training
    b. Cost
    c. Availability
    d. Cost-effective analysis.

To minimise bias, data extraction was undertaken by the lead researcher (OTJ) and by at least one other researcher independently (FMW or LJ). The research team met regularly to ensure consensus of views by discussing and resolving any differences in data extraction.

Quality Assessment Framework

Quality of the studies was assessed using the Joanna Briggs Institute Critical Appraisal Tools (Joanna Briggs Institute, 2016). The aim of the quality assessment was to determine the validity of the results based on the design, methods, analysis and conclusions of each study, and to assess the relative contribution of each study to the review. Limitations were acknowledged but studies were not excluded based on the quality assessment alone.

For peer review only

References

1. Dinnes J, Matin RN, Moreau JF, Patel L, Chan SA, Chuchu N, et al. Tests to assist in the diagnosis of cutaneous melanoma in adults: a generic protocol. Cochrane Database of Systematic Reviews. 2015.
2. Chappuis P, Duru G, Marchal O, Girier P, Dalle S, Thomas L. Dermoscopy, a useful tool for general practitioners in melanoma screening: A nationwide survey. British Journal of Dermatology. 2016;175:744-50.
3. Chia A, Trevena L. A Systematic Review of Training to Improve Melanoma Diagnostic Skills in General Practitioners. Journal of Cancer Education. 2016;31(4):730-5.
4. Koelink CJ, Jonkman MF, Van Der Meer K, Van Der Heide WK. Examination of skin lesions for cancer: which clinical decision aids and tools are available in general practice? European Journal of Dermatology. 2014;24(3):297-304.
5. Herschorn A. Dermoscopy for melanoma detection in family practice. Canadian Family Physician. 2012;58(7):740-5.
6. Harrington E, Clyne B, Wesseling N, Sandhu H, Armstrong L, Bennett H, et al. Diagnosing malignant melanoma in ambulatory care: A systematic review of clinical prediction rules. BMJ Open. 2017;7 (3) (no pagination)(e014096).
7. Koelink C, Vermeulen K, Kollen B, Bock G, Dekker J, Jonkman M, et al. Diagnostic accuracy and cost-effectiveness of dermoscopy in primary care: a cluster randomized clinical trial. Journal of the european academy of dermatology and venereology : JEADV [Internet]. 2014; 28(11):[1442-9 pp.].
8. Walter FM, Morris HC, Humphrys E, Hall PN, Prevost AT, Burrows N, et al. Effect of adding a diagnostic aid to best practice to manage suspicious pigmented lesions in primary care: randomised controlled trial. BMJ. 2012;345:e4110.
9. Plüddemann A, Heneghan C, Thompson M, Wolstenholme J, Price CP. Dermoscopy for the diagnosis of melanoma: Primary care diagnostic technology update. British Journal of General Practice. 2011;61(587):416-7.
10. Argenziano G, Puig S, Zalaudek I, Sera F, Corona R, Alsina M, et al. Dermoscopy improves accuracy of primary care physicians to triage lesions suggestive of skin cancer. Journal of Clinical Oncology. 2006;24(12):1877-82.
11. Gerbert B, Bronstone A, Maurer T, Berger T, McPhee SJ, Caspers N. The effectiveness of an Internet-based tutorial in improving primary care physicians' skin cancer triage skills. Journal of Cancer Education. 2002;17(1):7-11.

For peer review only

Appendix 2 – Search Strategies

The specific strategies for Medline, CENTRAL, EMBASE, CINAHL and SCOPUS were as follows:

A. Medline

1. primary health care.mp. or exp Primary Health Care/

2. general practice.mp. or exp General Practice/

3. family practice.mp. or exp Family Practice/

4. ((family or general or community) adj2 (doctor* or medicine or medical pratitioner* or medical practice* or practice* or practitioner* or healthcare* or health care*)).mp. [mp=title, abstract, original title, name of substance word, subject heading word, keyword heading word, protocol supplementary concept word, rare disease supplementary concept word, unique identifier, synonyms]

5. ((primary or community) adj2 (care* or health care* or healthcare* or medical care* or patient care*)).mp. [mp=title, abstract, original title, name of substance word, subject heading word, keyword heading word, protocol supplementary concept word, rare disease supplementary concept word, unique identifier, synonyms]

6. exp physicians, family/ or family physician*.mp.

7. (nurse* adj2 practi*).mp. [mp=title, abstract, original title, name of substance word, subject heading word, keyword heading word, protocol supplementary concept word, rare disease supplementary concept word, unique identifier, synonyms]

8. clinician with minimal dermoscopy training.mp.

9. melanoma screening clinic*.mp.

10. non-expert*.mp.

11. generalist clinician*.mp.

12. 1 or 2 or 3 or 4 or 5 or 6 or 7 or 8 or 9 or 10 or 11

13. dermoscopy.mp. or exp Dermoscopy/

14. dermatoscopy.mp.

15. epiluminescence microscopy.mp.

16. confocal microscopy.mp. or exp Microscopy, Confocal/

17. exp Photomicrography/ or photomicrograph.mp.

18. incident light microscopy.mp.

19. surface microscopy.mp.

20. dermoscopic.mp.

21. novel technique*.mp.

For peer review only

22. (siascope or siascopy or siascopic).mp. [mp=title, abstract, original title, name of substance word, subject heading word, keyword heading word, protocol supplementary concept word, rare disease supplementary concept word, unique identifier, synonyms]

23. molemate.mp.

24. AI.mp.

25. artificial intelligence.mp. or exp Artificial Intelligence/

26. exp Image Processing, Computer-Assisted/ or computer assisted.mp.

27. exp Diagnosis, Computer-Assisted/ or computer aided.mp.

28. exp "Neural Networks (Computer)"/ or neural network*.mp.

29. image analysis.mp.

30. automatic classification.mp.

31. machine learning.mp. or exp Machine Learning/

32. computer vision.mp.

33. digital analysis.mp.

34. digitally analysed.mp.

35. (analysis adj5 digitally).mp. [mp=title, abstract, original title, name of substance word, subject heading word, keyword heading word, protocol supplementary concept word, rare disease supplementary concept word, unique identifier, synonyms]

36. computer diagnos*.mp.

37. exp Mobile Applications/ or mobile app*.mp.

38. smartphone app*.mp. or exp Smartphone/

39. recognition skill*.mp.

40. automatic detection.mp.

41. collective intelligence assessment*.mp.

42. analysis device*.mp.

43. aid to diagnosis.mp.

44. diagnostic aid*.mp.

45. (decision adj3 tool*).mp. [mp=title, abstract, original title, name of substance word, subject heading word, keyword heading word, protocol supplementary concept word, rare disease supplementary concept word, unique identifier, synonyms]

46. tele-dermatos*.mp. [mp=title, abstract, original title, name of substance word, subject heading word, keyword heading word, protocol supplementary concept word, rare disease supplementary concept word, unique identifier, synonyms]

For peer review only

47. tele-dermos*.mp. [mp=title, abstract, original title, name of substance word, subject heading word, keyword heading word, protocol supplementary concept word, rare disease supplementary concept word, unique identifier, synonyms]

48. ((dermosc* or dermatosc* or epiluminescence microscopy or surface microscopy) adj3 (image* or picture* or photo*)).mp. [mp=title, abstract, original title, name of substance word, subject heading word, keyword heading word, protocol supplementary concept word, rare disease supplementary concept word, unique identifier, synonyms]

49. 13 or 14 or 15 or 16 or 17 or 18 or 19 or 20 or 21 or 22 or 23 or 24 or 25 or 26 or 27 or 28 or 29 or 30 or 31 or 32 or 33 or 34 or 35 or 36 or 37 or 38 or 39 or 40 or 41 or 42 or 43 or 44 or 45 or 46 or 47 or 48

50. exp Melanoma/ or melanoma*.mp.

51. exp Skin Neoplasms/ or skin neoplasm*.mp.

52. pigmented nevus.mp. or exp Nevus, Pigmented/

53. (naevocarcinoma or nevocarcinoma).mp. [mp=title, abstract, original title, name of substance word, subject heading word, keyword heading word, protocol supplementary concept word, rare disease supplementary concept word, unique identifier, synonyms]

54. ((neoplasm* or cancer* or tumour*) adj3 (skin or pigment*)).mp. [mp=title, abstract, original title, name of substance word, subject heading word, keyword heading word, protocol supplementary concept word, rare disease supplementary concept word, unique identifier, synonyms]

55. ((suspicious or melanocytic or pigmented) adj3 (lesion* or mole* or naevus or nevus or naevi or nevi or skin)).mp. [mp=title, abstract, original title, name of substance word, subject heading word, keyword heading word, protocol supplementary concept word, rare disease supplementary concept word, unique identifier, synonyms]

56. skin lesion*.mp.

57. 50 or 51 or 52 or 53 or 54 or 55 or 56

58. 12 and 49 and 57

For peer review only

B. CENTRAL

1. "primary health care":ti,ab,kw (Word variations have been searched)

2. "general practice":ti,ab,kw (Word variations have been searched)

3. "family practice":ti,ab,kw (Word variations have been searched)

4. family physician*:ti,ab,kw (Word variations have been searched)

5. MeSH descriptor: [Primary Health Care] explode all trees

6. MeSH descriptor: [General Practice] explode all trees

7. MeSH descriptor: [Family Practice] explode all trees

8. MeSH descriptor: [Physicians, Family] explode all trees

9. ((family or general or community) near/2 (doctor* or medicine or medical practitioner* or medical practice* or practice* or practitioner* or healthcare* or health care*)):ti,ab,kw (Word variations have been searched)

10. ((primary or community) near/2 (care* or health care* of healthcare* or medical care* or patient care*)):ti,ab,kw (Word variations have been searched)

11. (nurse* near/2 practi*):ti,ab,kw (Word variations have been searched)

12. clinician with minimal dermoscopy training:ti,ab,kw (Word variations have been searched)

13. melanoma screening clinic*:ti,ab,kw (Word variations have been searched)

14. non-expert*:ti,ab,kw (Word variations have been searched)

15. generalist clinician*:ti,ab,kw (Word variations have been searched)

16. #1 or #2 or #3 or #4 or #5 or #6 or #7 or #8 or #9 or #10 or #11 or #12 or #13 or #14 or #15

17. "dermoscopy":ti,ab,kw (Word variations have been searched)

18. dermatoscopy:ti,ab,kw (Word variations have been searched)

19. epiluminescence microscopy:ti,ab,kw (Word variations have been searched)

20. confocal microscopy:ti,ab,kw (Word variations have been searched)

21. photomicrograph:ti,ab,kw (Word variations have been searched)

22. incident light microscopy:ti,ab,kw (Word variations have been searched)

23. "surface microscopy":ti,ab,kw (Word variations have been searched)

24. "dermoscopic":ti,ab,kw (Word variations have been searched)

25. novel technique*:ti,ab,kw (Word variations have been searched)

26. siascop*:ti,ab,kw (Word variations have been searched)

27. molemate:ti,ab,kw (Word variations have been searched)

28. AI:ti,ab,kw (Word variations have been searched)

For peer review only

29. "artificial intelligence":ti,ab,kw (Word variations have been searched)

30. "computer assisted":ti,ab,kw (Word variations have been searched)

31. "computer aided":ti,ab,kw (Word variations have been searched)

32. "neural network":ti,ab,kw (Word variations have been searched)

33. image analysis:ti,ab,kw (Word variations have been searched)

34. automatic classification:ti,ab,kw (Word variations have been searched)

35. "machine learning":ti,ab,kw (Word variations have been searched)

36. computer vision:ti,ab,kw (Word variations have been searched)

37. digital analysis:ti,ab,kw (Word variations have been searched)

38. digitally analysed:ti,ab,kw (Word variations have been searched)

39. digitally analysed:ti,ab,kw (Word variations have been searched)

40. analysis near/5 digitally:ti,ab,kw (Word variations have been searched)

41. computer diagnos*:ti,ab,kw (Word variations have been searched)

42. mobile app*:ti,ab,kw (Word variations have been searched)

43. smartphone app*:ti,ab,kw (Word variations have been searched)

44. recognition skill*:ti,ab,kw (Word variations have been searched)

45. automatic detection:ti,ab,kw (Word variations have been searched)

46. collective intelligence assessment*:ti,ab,kw (Word variations have been searched)

47. analysis device*:ti,ab,kw (Word variations have been searched)

48. aid to diagnosis:ti,ab,kw (Word variations have been searched)

49. diagnostic aid*:ti,ab,kw (Word variations have been searched)

50. decision near/3 tool*:ti,ab,kw (Word variations have been searched)

51. tele-dermatos*:ti,ab,kw (Word variations have been searched)

52. tele-dermos*:ti,ab,kw (Word variations have been searched)

53. ((dermoscopy or dermatoscopy or epiluminescence microscopy or surface microscopy or dermoscopic) near/3 (image* or picture* or photo*)):ti,ab,kw (Word variations have been searched)

54. MeSH descriptor: [Dermoscopy] explode all trees

55. MeSH descriptor: [Microscopy, Confocal] explode all trees

56. MeSH descriptor: [Photomicrography] explode all trees

57. MeSH descriptor: [Artificial Intelligence] explode all trees

58. MeSH descriptor: [Diagnosis, Computer-Assisted] explode all trees

For peer review only

59. MeSH descriptor: [Image Processing, Computer-Assisted] explode all trees

60. MeSH descriptor: [Neural Networks (Computer)] explode all trees

61. MeSH descriptor: [Machine Learning] explode all trees

62. MeSH descriptor: [Mobile Applications] explode all trees

63. MeSH descriptor: [Smartphone] explode all trees

64. #17 or #18 or #19 or #20 or #21 or #22 or #23 or #24 or #25 or #26 or #27 or #28 or #29 or #30 or #31 or #32 or #33 or #34 or #35 or #36 or #37 or #38 or #39 or #40 or #41 or #42 or #43 or #44 or #45 or #46 or #47 or #48 or #49 or #50 or #51 or #52 or #53 or #54 or #55 or #56 or #57 or #58 or #59 or #60 or #61 or #62 or #63

65. "skin neoplasm*":ti,ab,kw (Word variations have been searched)

66. melanoma*:ti,ab,kw (Word variations have been searched)

67. "pigmented nevus":ti,ab,kw (Word variations have been searched)

68. skin lesion*:ti,ab,kw (Word variations have been searched)

69. naevocarcinoma or nevocarcinoma:ti,ab,kw (Word variations have been searched)

70. ((neoplasm* or cancer* or tumour*) near/3 (skin or pigment*)):ti,ab,kw (Word variations have been searched)

71. ((suspicious or melanocytic or pigmented) near/3 (lesion* or mole* or naevus or nevus or naevi or nevi or skin)):ti,ab,kw (Word variations have been searched)

72. MeSH descriptor: [Melanoma] explode all trees

73. MeSH descriptor: [Skin Neoplasms] explode all trees

74. MeSH descriptor: [Nevus, Pigmented] explode all trees

75. #65 or #66 or #67 or #68 or #69 or #70 or #71 or #72 or #73 or #74

76. #16 and #64 and #75

For peer review only

C. EMBASE

1. exp *primary health care/
2. primary health care.ti,ab.
3. exp *general practice/
4. general practice.ti,ab.
5. family practice.ti,ab.
6. ((family or general or community) adj2 (doctor* or medicine or medical practitioner* or medical practice* or practice* or practitioner* or healthcare* or health care*)).ti,ab.
7. ((primary or community) adj2 (care* or health care* or healthcare* or medical care* or patient care*)).ti,ab.
8. family physician*.ti,ab.
9. (nurse* adj2 practi*).ti,ab.
10. clinician with minimal dermoscopy training.ti,ab.
11. melanoma screening clinic*.ti,ab.
12. non-expert*.ti,ab.
13. generalist clinician*.ti,ab.
14. 1 or 2 or 3 or 4 or 5 or 6 or 7 or 8 or 9 or 10 or 11 or 12 or 13
15. exp *epiluminescence microscopy/
16. epiluminescence microscopy.ti,ab.
17. dermoscopy.ti,ab.
18. dermatoscopy.ti,ab.
19. exp *confocal microscopy/
20. confocal microscopy.ti,ab.
21. photomicrograph*.ti,ab.
22. incident light microscopy.ti,ab.
23. surface microscopy.ti,ab.
24. dermoscopic.ti,ab.
25. novel technique*.ti,ab.
26. (siascope or siascopy or siascopic).ti,ab.
27. molemate.ti,ab.
28. AI.ti,ab.
29. exp *artificial intelligence/
30. artificial intelligence.ti,ab.
31. exp computer assisted diagnosis/
32. computer assisted.ti,ab.
33. exp computer analysis/
34. computer aided.ti,ab.
35. computer analysis.ti,ab.
36. exp *artificial neural network/
37. neural network*.ti,ab.
38. exp *image analysis/
39. image analysis.ti,ab.
40. automatic classification.ti,ab.
41. exp *machine learning/
42. machine learning.ti,ab.
43. computer vision.ti,ab.
44. digital analysis.ti,ab.
45. digitally analy*.ti,ab.
46. (analysis adj5 digitally).ti,ab.
47. computer diagnos*.ti,ab.

For peer review only

48. exp *mobile application/
49. mobile app*.ti,ab.
50. exp *smartphone/
51. smartphone app*.ti,ab.
52. recognition skill*.ti,ab.
53. automatic detection.ti,ab.
54. collective intelligence assessment*.ti,ab.
55. analysis device*.ti,ab.
56. aid to diagnosis.ti,ab.
57. diagnostic aid*.ti,ab.
58. (decision adj3 tool*).ti,ab.
59. tele-dermatos*.ti,ab.
60. tele-dermos*.ti,ab.
61. ((dermoscopy or dermatoscopy or epiluminescence microscopy or surface microscopy or dermoscopic) adj3 (image* or picture* or photo*)).ti,ab.
62. 15 or 16 or 17 or 18 or 19 or 20 or 21 or 22 or 23 or 24 or 25 or 26 or 27 or 28 or 29 or 30 or 31 or 32 or 33 or 34 or 35 or 36 or 37 or 38 or 39 or 40 or 41 or 42 or 43 or 44 or 45 or 46 or 47 or 48 or 49 or 50 or 51 or 52 or 53 or 54 or 55 or 56 or 57 or 58 or 59 or 60 or 61
63. exp *melanoma/ or exp *melanoma skin cancer/
64. melanoma*.ti,ab.
65. exp *skin tumor/ or exp *skin cancer/
66. skin neoplasm*.ti,ab.
67. exp *pigmented nevus/
68. pigmented naevus.ti,ab.
69. skin lesion*.ti,ab.
70. (naevocarcinoma or nevocarcinoma).ti,ab.
71. ((neoplasm* or cancer* or tumour*) adj3 (skin or pigment*)).ti,ab.
72. ((suspicious or melanocytic or pigmented) adj3 (lesion* or mole* or naevus or nevus or naevi or nevi or skin)).ti,ab.
73. 63 or 64 or 65 or 66 or 67 or 68 or 69 or 70 or 71 or 72
74. 14 and 62 and 73

For peer review only

D. CINAHL

66 S15 AND S56 AND S65

65 S57 OR S58 OR S59 OR S60 OR S61 OR S62 OR S63 OR S64

64 (MH "Skin Neoplasms+")

63 (MH "Melanoma+")

62 ((suspicious or melanocytic or pigment*) N3 (lesion* or mole* or naevus or nevus or naevi or nevi or skin))

61 ((tumour* or neoplasm* or cancer*) N3 (skin or pigment*))

60 naevocarcinoma or nevocarcinoma

59 skin lesion*

58 skin neoplasm*

57 melanoma*

56 S16 OR S17 OR S18 OR S19 OR S20 OR S21 OR S22 OR S23 OR S24 OR S25 OR S26 OR S27 OR S28 OR S29 OR S30 OR S31 OR S32 OR S33 OR S34 OR S35 OR S36 OR S37 OR S38 OR S39 OR S40 OR S41 OR S42 OR S43 OR S44 OR S45 OR S46 OR S47 OR S48 OR S49 OR S50 OR S51 OR S52 OR S53 OR S54 OR S55

55 (MH "Smartphone+")

54 (MH "Mobile Applications")

53 (MH "Neural Networks (Computer)")

52 (MH "Image Processing, Computer Assisted+") OR (MH "Image Interpretation, Computer Assisted+") OR (MH "Diagnosis, Computer Assisted+")

51 (MH "Artificial Intelligence+")

50 ((dermoscop* or dermatoscopy or epiluminescence microscopy or surface microscopy) N3 (image* or picture* or photo*))

49 tele-dermatos*

48 tele-dermos*

47 decision N3 tool*

46 diagnos* N4 aid*

45 analysis device*

44 collective intelligence assessment*

43 automatic detection

42 recognition skill*

41 smartphone app*

40 mobile app*

39 computer diagnos*

38 analysis N5 digital*

37 digitally analysed

36 digital analysis

35 computer vision

34 machine learning

33 automatic classification

32 image analysis

31 neural network*

30 computer aided

29 computer assisted

28 artificial intelligence

27 AI

26 molemate

25 siascop*

24 novel technique*

23 dermoscopic

For peer review only

22 surface microscopy
21 incident light microscopy
20 photomicrograph
19 confocal microscopy
18 epiluminescence microscopy
17 dermatoscopy
16 dermoscopy
15 S1 OR S2 OR S3 OR S4 OR S5 OR S6 OR S7 OR S8 OR S9 OR S10 OR S11 OR S12 OR S13 OR S14
14 (MH "Physicians, Family")
13 (MH "Family Practice")
12 (MH "Primary Health Care")
11 generalist clinician*
10 non-expert*
9 melanoma screening clinic*
8 clinician with minimal dermoscopy training
7 nurse* N2 practi*
6 family physician*
5 ((primary or community) N2 (care* or health care* or healthcare* or medical care* or patient care*))
4 ((family or general or community) N2 (doctor* or medicine or medical practitioner* or medical practice* or practice* or practitioner* or healthcare* or health care*))
3 family practice
2 general practice
1 primary health care

For peer review only

E. SCOPUS

(((TITLE-ABS-KEY(primary w/1 (health w/1 care))) OR (TITLE-ABS-KEY(general w/1 practice)) OR (TITLE-ABS-KEY(family w/1 practice)) OR (TITLE-ABS-KEY(family w/1 physician*)) OR (TITLE-ABS-KEY((family W/2 doctor*) OR (family W/2 medicine) OR (family W/2 (medical w/1 practitioner*)) OR (family W/2 (medical w/1 practice*)) OR (family W/2 practice*) OR (family W/2 practitioner*) OR (family W/2 healthcare*) OR (family W/2 (health w/1 care*)))) OR (TITLE-ABS-KEY((general W/2 doctor*) OR (general W/2 medicine) OR (general W/2 (medical w/1 practitioner*)) OR (general W/2 (medical w/1 practice*)) OR (general W/2 practice*) OR (general W/2 practitioner*) OR (general W/2 healthcare*) OR (general W/2 (health w/1 care*)))) OR (TITLE-ABS-KEY((community W/2 doctor*) OR (community W/2 medicine) OR (community W/2 (medical w/1 practitioner*)) OR (community W/2 (medical w/1 practice*)) OR (community W/2 practice*) OR (community W/2 practitioner*) OR (community W/2 healthcare*) OR (community W/2 (health w/1 care*))))) OR ((TITLE-ABS-KEY((community W/2 care*) OR (community W/2 (medical w/1 care*)) OR (community W/2 (patient w/1 care*)) OR (community W/2 healthcare*) OR (community W/2 (health w/1 care*)))) OR (TITLE-ABS-KEY((primary W/2 care*) OR (primary W/2 (medical w/1 care*)) OR (primary W/2 (patient w/1 care*)) OR (primary W/2 healthcare*) OR (primary W/2 (health w/1 care*)))) OR (TITLE-ABS-KEY(nurs* W/2 practi*)) OR (TITLE-ABS-KEY(clinician w/1 with w/1 minimal w/1 dermoscopy w/1 training)) OR (TITLE-ABS-KEY(melanoma w/1 screening w/1 clinic*)) OR (TITLE-ABS-KEY(generalist w/1 clinician*))))

AND

(((TITLE-ABS-KEY(surface w/1 microscopy)) OR (TITLE-ABS-KEY(dermoscopic)) OR (TITLE-ABS-KEY(novel w/1 technique)) OR (TITLE-ABS-KEY(siascop*)) OR (TITLE-ABS-KEY(molemate)) OR (TITLE-ABS-KEY(ai)) OR (TITLE-ABS-KEY(artificial w/1 intelligence))) OR ((TITLE-ABS-KEY(computer w/1 assisted)) OR (TITLE-ABS-KEY(computer w/1 aided)) OR (TITLE-ABS-KEY(neural w/1 network)) OR (TITLE-ABS-KEY(image w/1 analysis)) OR (TITLE-ABS-KEY(automatic w/1 classification)) OR (TITLE-ABS-KEY(machine w/1 learning)) OR (TITLE-ABS-KEY(computer w/1 vision))) OR ((TITLE-ABS-KEY(digital* W/5 analys*)) OR (TITLE-ABS-KEY(computer w/1 diagnos*)) OR (TITLE-ABS-KEY(mobile w/1 app*)) OR (TITLE-ABS-KEY(smartphone)) OR (TITLE-ABS-KEY(recognition w/1 skill*)) OR (TITLE-ABS-KEY(automatic w/1 detection)) OR (TITLE-ABS-KEY(collective w/1 intelligence AND assessment*))) OR ((TITLE-ABS-KEY(analysis w/1 device*)) OR (TITLE-ABS-KEY(diagnos* W/3 aid*)) OR (TITLE-ABS-KEY(decision W/3 aid*)) OR (TITLE-ABS-KEY(decision W/3 tool*)) OR (TITLE-ABS-KEY(tele-dermos*)) OR (TITLE-ABS-KEY(tele-dermatos*)) OR (TITLE-ABS-KEY((dermos* W/3 image*) OR (dermos* W/3 photo*) OR (dermos* W/3 picture*)))) OR ((TITLE-ABS-KEY((dermatos* W/3 image*) OR (dermatos* W/3 photo*) OR (dermatos* W/3 picture*))) OR (TITLE-ABS-KEY((epiluminescence W/4 image*) OR (epiluminescence W/4 photo*) OR (epiluminescence W/4 picture*))) OR (TITLE-ABS-KEY(((surface w/1 microscopy) W/4 image*) OR ((surface w/1 microscopy) W/4 photo*) OR ((surface w/1 microscopy) W/4 picture*)))) OR ((TITLE-ABS-KEY(dermoscopy)) OR (TITLE-ABS-KEY(dermatoscopy)) OR (TITLE-ABS-KEY(epiluminescence w/1 microscopy)) OR (TITLE-ABS-KEY(confocal w/1 microscopy)) OR (TITLE-ABS-KEY(photomicrograph)) OR (TITLE-ABS-KEY(incident w/1 light w/1 microscopy))))

AND

((TITLE-ABS-KEY(melanoma)) OR (TITLE-ABS-KEY(naevocarcinoma OR nevocarcinoma)) OR (TITLE-ABS-KEY((pigment* W/3 neoplasm*) OR (pigment* W/3 cancer*) OR (pigment* W/3 tumo*) OR (pigment* W/3 lesion*))) OR (TITLE-ABS-KEY((pigment* W/3 lesion*) OR (pigment* W/3 mole*) OR (pigment* W/3 naev*) OR (pigment* W/3 nev*) OR (pigment* W/3 skin))) OR (TITLE-ABS-KEY((melanocytic W/3 lesion*) OR (melanocytic W/3 mole*) OR (melanocytic W/3 naev*) OR (melanocytic W/3 nev*) OR (melanocytic W/3 skin))) OR (TITLE-ABS-KEY((suspicious W/3 lesion*) OR (suspicious W/3 mole*) OR (suspicious W/3 naev*) OR (suspicious W/3 nev*) OR (suspicious W/3 skin))) OR (TITLE-ABS-KEY((skin W/3 neoplasm*) OR (skin W/3 cancer*) OR (skin W/3 tumo*) OR (skin W/3 lesion*))))

For peer review only

Appendix 3 – Full list of studies that underwent full text review, their assessors and outcomes

Paper	Assessor					Reason for exclusion				
	OJ	LJ	SH	FW	MvM	No dermoscopy use	Not in 1ary care	Not peer reviewed / conference abstract	No primary data	Clinical Audit
Affifi *et al*. 2017 (A)	Exclude		Exclude			■				
Affifi *et al*. 2017 (B)	Exclude		Exclude			■				
Ahmadi *et al*. 2017	Include	Include								
Alonso *et al*. 2016	Exclude		Exclude					■		
Argenziano *et al*. 2006	Include		Include							
Baldi *et al*. 2010	Exclude	Exclude							■	
Bataille *et al*. 2011	Exclude	Exclude						■		
Bergman *et al*. 2008	Exclude				Exclude				■	
Bergstrom *et al*. 2012	Exclude								■	
Berryman *et al*. 2012	Exclude		Exclude			■				
Blackledge *et al*. 2009	Exclude	Exclude				■				
Borve *et al*. 2015	Include	Include								
Bourne *et al*. 2012	Include			Include						
Bowns *et al*. 2006	Exclude			Exclude			■			
Brinkhuijsen *et al.* 2008	Exclude				Exclude				■	
Brown *et al*. 2000	Exclude		Exclude						■	
Carli *et al*. 2007	Exclude		Exclude						■	
Carrara *et al*. 2007	Exclude	Exclude				■				
Chao Li *et al*. 2013	Exclude			Exclude				■		
Chappuis *et al*. 2016	Include		Include							
Chen *et al*. 2011	Exclude	Exclude		Exclude				■		
Congalton *et al*. 2015	Exclude			Exclude			■			

Cook *et al*. 2015	Exclude	Exclude								
Coras *et al*. 2003	Exclude	Exclude								
Dahlen Gyllencreutz *et al*. 2017	Exclude			Exclude						
Del Mar *et al*. 1995	Exclude		Exclude							
Dolianitis *et al*. 2005	Exclude			Exclude						
Drugge *et al*. 2010	Exclude	Exclude								
Ferrandiz *et al*. 2017	Include			Include						
Greenberg *et al*. 2012	Exclude	Exclude								
Griffiths *et al*. 2010	Exclude			Exclude						
Grimaldi *et al*. 2009	Include			Include						
Hennessy *et al*. 2010	Exclude		Exclude							
Jain *et al*. 2012	Exclude	Exclude	Exclude							
Joliffe *et al*. 2001 (A)	Exclude			Exclude						
Joliffe *et al*. 2001 (B)	Exclude			Exclude						
Kenney *et al*. 2016	Exclude			Exclude						
Kitchener *et al*. 2009	Exclude	Exclude	Exclude							
Koelink *et al*. 2014	Include	Include								
Kroemer *et al*. 2001	Exclude			Exclude						
Laheru *et al*. 2017	Exclude			Exclude						
Livingstone *et al*. 2015	Include			Include						
Lorentzen *et al*. 1999	Exclude	Exclude		Exclude						
MacKinnon *et al*. 2016	Exclude		Exclude							
Mahendran *et al*. 2005	Exclude	Exclude								
Masood *et al*. 2014 (A)	Exclude	Exclude								
Masood *et al*. 2014 (B)	Exclude		Exclude							
Masood *et al*. 2015	Exclude		Exclude							
Massone *et al*. 2014	Exclude			Exclude						
Menzies *et al*. 2005	Include	Include								
Menzies *et al*. 2009	Include		Include							

For peer review only

Mikolajcyk *et al*. 2017	Exclude	Exclude								
Monheit *et al*. 2011	Exclude		Exclude							
Moreno-Ramirez *et al*. 2006	Include			Include						
Moreno-Ramirez *et al*. 2007	Exclude	Exclude								
Morris *et al*. 2017 (A)	Include		Include							
Morris *et al*. 2017 (B)	Include	Include								
Morton *et al*. 2011	Exclude			Exclude						
Naka *et al*. 2017	Exclude	Exclude		Exclude						
Nelson *et al*. 2016	Exclude			Exclude						
Pagnanelli *et al*. 2003	Include		Include							
Perakis *et al*. 2014	Exclude	Exclude								
Phelan *et al*. 2008	Exclude			Exclude						
Picollo *et al*. 2000	Exclude			Exclude						
Picollo *et al*. 2002	Exclude			Exclude						
Rademaker *et al*. 2010	Exclude			Exclude						
Riding *et al*. 2016	Exclude		Exclude							
Robson *et al*. 2012*	Exclude	Exclude								
Rogers *et al*. 2016	Include			Include						
Rogers *et al*. 2017	Include			Include						
Rosendahl *et al*. 2011	Include	Include								
Rosendahl *et al*. 2012 (A)	Exclude	Exclude	Exclude							
Rosendahl *et al*. 2012 (B)	Include			Include						
Secker *et al*. 2014	Include	Include								
Shapiro *et al*. 2004	Exclude		Exclude							
Sheraz *et al*. 2011	Exclude	Exclude								
Singh *et al*. 2016	Exclude	Exclude	Exclude							
Skrovseth *et al*. 2010	Exclude	Exclude								
Smith *et al*. 2000	Exclude	Exclude								
Soyer *et al*. 2004	Exclude		Exclude	Exclude						

For peer review only

Stratton *et al*. 2016	Include	Include		Include						
Swanson *et al*. 2017	Exclude	Exclude								
Tan *et al*. 2010	Exclude			Exclude						
Tan *et al*. 2015	Exclude		Exclude							
Thomas *et al*. 2017	Exclude	Exclude								
Van der Heijden *et al*. 2013	Include			Include						
Wadhawan *et al*. 2011	Exclude			Exclude						
Watts *et al*. 2017	Exclude		Exclude							
Westerhoff *et al*. 2000	Include	Include								
Winkelmann *et al*. 2015 (A)	Exclude		Exclude							
Winkelmann *et al*. 2015 (B)	Exclude		Exclude							
Wray *et al*. 2015	Exclude			Exclude						
Zalaudek *et al*. 2008	Exclude			Exclude						
Zortea *et al*. 2014	Exclude		Exclude							
Zouridakis *et al*. 2015	Exclude	Exclude	Exclude							

For peer review only

Reporting checklist for systematic review and meta-analysis.

Based on the PRISMA guidelines.

Instructions to authors

Complete this checklist by entering the page numbers from your manuscript where readers will find each of the items listed below.

Your article may not currently address all the items on the checklist. Please modify your text to include the missing information. If you are certain that an item does not apply, please write "n/a" and provide a short explanation.

Upload your completed checklist as an extra file when you submit to a journal.

In your methods section, say that you used the PRISMA reporting guidelines, and cite them as:

Moher D, Liberati A, Tetzlaff J, Altman DG, The PRISMA Group. Preferred Reporting Items for Systematic Reviews and Meta-Analyses: The PRISMA Statement

		Reporting Item	Page Number
	#1	Identify the report as a systematic review, meta-analysis, or both.	1
Structured summary	#2	Provide a structured summary including, as applicable: background; objectives; data sources; study eligibility criteria, participants, and interventions; study appraisal and synthesis methods; results; limitations; conclusions and implications of key findings; systematic review registration number	2
Rationale	#3	Describe the rationale for the review in the context of what is already known.	3
Objectives	#4	Provide an explicit statement of questions being addressed with reference to participants, interventions, comparisons, outcomes, and study design (PICOS).	3
Protocol and	#5	Indicate if a review protocol exists, if and where it can be	4

For peer review only

registration		accessed (e.g., Web address) and, if available, provide registration information including the registration number.	
Eligibility criteria	#6	Specify study characteristics (e.g., PICOS, length of follow-up) and report characteristics (e.g., years considered, language, publication status) used as criteria for eligibility, giving rational	4
Information sources	#7	Describe all information sources in the search (e.g., databases with dates of coverage, contact with study authors to identify additional studies) and date last searched.	4
Search	#8	Present full electronic search strategy for at least one database, including any limits used, such that it could be repeated.	Appendix 2
Study selection	#9	State the process for selecting studies (i.e., for screening, for determining eligibility, for inclusion in the systematic review, and, if applicable, for inclusion in the meta-analysis).	See note 1
Data collection process	#10	Describe the method of data extraction from reports (e.g., piloted forms, independently by two reviewers) and any processes for obtaining and confirming data from investigators.	See note 2
Data items	#11	List and define all variables for which data were sought (e.g., PICOS, funding sources), and any assumptions and simplifications made.	Appendix 1
Risk of bias in individual studies	#12	Describe methods used for assessing risk of bias in individual studies (including specification of whether this was done at the study or outcome level, or both), and how this information is to be used in any data synthesis.	See note 3
Summary measures	#13	State the principal summary measures (e.g., risk ratio, difference in means).	4
Planned methods of analyis	#14	Describe the methods of handling data and combining results of studies, if done, including measures of consistency (e.g., I2) for each meta-analysis.	4
Risk of bias across studies	#15	Specify any assessment of risk of bias that may affect the cumulative evidence (e.g., publication bias, selective reporting within studies).	4
Additional	#16	Describe methods of additional analyses (e.g., sensitivity or	4

For peer review only

analyses		subgroup analyses, meta-regression), if done, indicating which were pre-specified.	
Study selection	#17	Give numbers of studies screened, assessed for eligibility, and included in the review, with reasons for exclusions at each stage, ideally with a flow diagram.	See note 4
Study characteristics	#18	For each study, present characteristics for which data were extracted (e.g., study size, PICOS, follow-up period) and provide the citation.	See note 5
Risk of bias within studies	#19	Present data on risk of bias of each study and, if available, any outcome-level assessment (see Item 12).	See note 6
Results of individual studies	#20	For all outcomes considered (benefits and harms), present, for each study: (a) simple summary data for each intervention group and (b) effect estimates and confidence intervals, ideally with a forest plot.	See note 7
Synthesis of results	#21	Present the main results of the review. If meta-analyses are done, include for each, confidence intervals and measures of consistency.	pages 5-22
Risk of bias across studies	#22	Present results of any assessment of risk of bias across studies (see Item 15).	N/a
Additional analysis	#23	Give results of additional analyses, if done (e.g., sensitivity or subgroup analyses, meta-regression [see Item 16]).	N/a
Summary of Evidence	#24	Summarize the main findings, including the strength of evidence for each main outcome; consider their relevance to key groups (e.g., health care providers, users, and policy makers	22
Limitations	#25	Discuss limitations at study and outcome level (e.g., risk of bias), and at review level (e.g., incomplete retrieval of identified research, reporting bias).	23
Conclusions	#26	Provide a general interpretation of the results in the context of other evidence, and implications for future research.	23
Funding	#27	Describe sources of funding or other support (e.g., supply of data) for the systematic review; role of funders for the	24

For peer review only

systematic review.

Author notes

1. 4 and appendix 1

2. 4 and appendix 1

3. 4 & 5 & appendix 1

4. 5 and PRISMA diagram (fig.1)

5. Pages 5, 8 and 10 (tables 1, 2 & 3)

6. Page 8 & 9 (table 2)

7. Pages 10-19

The PRISMA checklist is distributed under the terms of the Creative Commons Attribution License CC-BY. This checklist was completed on 26. October 2018 using http://www.goodreports.org/, a tool made by the EQUATOR Network in collaboration with Penelope.ai

For peer review only
